# TERT Promotes Epithelial Proliferation through Transcriptional Control of a Myc- and Wnt-Related Developmental Program

**DOI:** 10.1371/journal.pgen.0040010

**Published:** 2008-01-18

**Authors:** Jinkuk Choi, Lucinda K Southworth, Kavita Y Sarin, Andrew S Venteicher, Wenxiu Ma, Woody Chang, Peggie Cheung, Sohee Jun, Maja K Artandi, Naman Shah, Stuart K Kim, Steven E Artandi

**Affiliations:** 1 Department of Medicine, Stanford School of Medicine, Stanford, California, United States of America; 2 Cancer Biology Program, Stanford School of Medicine, Stanford, California, United States of America; 3 Department of Genetics, Stanford School of Medicine, Stanford, California, United States of America; 4 Biomedical Informatics Program, Stanford School of Medicine, Stanford, California, United States of America; 5 Department of Computer Science, Stanford University, Stanford, California, United States of America; 6 Department of Developmental Biology, Stanford School of Medicine, Stanford, California, United States of America; The Jackson Laboratory, United States of America

## Abstract

Telomerase serves a critical role in stem cell function and tissue homeostasis. This role depends on its ability to synthesize telomere repeats in a manner dependent on the reverse transcriptase (RT) function of its protein component telomerase RT (TERT), as well as on a novel pathway whose mechanism is poorly understood. Here, we use a TERT mutant lacking RT function (TERT^ci^) to study the mechanism of TERT action in mammalian skin, an ideal tissue for studying progenitor cell biology. We show that TERT^ci^ retains the full activities of wild-type TERT in enhancing keratinocyte proliferation in skin and in activating resting hair follicle stem cells, which triggers initiation of a new hair follicle growth phase and promotes hair synthesis. To understand the nature of this RT-independent function for TERT, we studied the genome-wide transcriptional response to acute changes in TERT levels in mouse skin. We find that TERT facilitates activation of progenitor cells in the skin and hair follicle by triggering a rapid change in gene expression that significantly overlaps the program controlling natural hair follicle cycling in wild-type mice. Statistical comparisons to other microarray gene sets using pattern-matching algorithms revealed that the TERT transcriptional response strongly resembles those mediated by Myc and Wnt, two proteins intimately associated with stem cell function and cancer. These data show that TERT controls tissue progenitor cells via transcriptional regulation of a developmental program converging on the Myc and Wnt pathways.

## Author Summary

Stem cells and progenitor cells within a tissue are required to maintain tissue homeostasis and to repair tissues after injury by giving rise to differentiated daughter cells. Many progenitor cells express telomerase, a reverse transcriptase enzyme that adds DNA repeats to telomeres, the protective structures that cap chromosome ends. Telomere addition by telomerase is important for normal progenitor cell function and is crucial for enabling cancer cells to divide an unlimited number of times. In addition to its telomere-lengthening function, telomerase reverse transcriptase (TERT) can directly activate quiescent epidermal stem cells. However, the mechanism underlying this novel function for TERT is still not understood. In this study, we demonstrate that the catalytic activity of TERT is dispensable for its ability to activate tissue progenitor cells in vivo. Furthermore, using gene microarrays, we show that TERT controls a developmental program that overlaps the natural transcriptional program of hair follicle cycling in mouse skin. Using pattern-matching algorithms, we find that the TERT-controlled genetic program significantly resembles programs regulated by Myc and Wnt, two pathways critical for stem cell function and tumorigenesis. This paper reveals critical new insights into novel mechanisms of non-telomerase functions of TERT, identifying TERT as a developmental regulator linked to control of transcriptional responses.

## Introduction

Telomerase exhibits two activities that profoundly influence tissue progenitor cells. Through its action in synthesizing telomere repeats, telomerase is required to maintain progenitor cell viability and self-renewal. In settings of insufficient telomerase, telomere shortening blunts stem cell self-renewal, dramatically impairing tissue function [1,2]. In addition to this telomere maintenance function, TERT, the telomerase protein catalytic subunit, can directly enhance cell cycle entry of quiescent epidermal stem cells [3,4]. However, the mechanisms through which TERT activates tissue stem cells via this second, non-canonical pathway are not understood.

Telomerase, which comprises both TERT and the telomerase RNA (TERC), adds telomere repeats to chromosome ends to offset the loss of telomere sequences that occurs due to the end-replication problem, the inability of DNA polymerase to replicate fully the lagging DNA strand. In the absence of sufficient levels of telomerase, telomeres shorten progressively with cell division, ultimately leading to loss of telomere protection and a DNA damage response that induces senescence or cell death [5]. Loss of telomerase in mouse impairs the function of self-renewing tissues as these DNA damage responses at uncapped chromosome ends induce apoptosis and block proliferation [1]. The defects in tissue maintenance in telomerase knockout mice with short telomeres is likely due to impaired progenitor cell function, because telomere shortening significantly reduces stem cell self-renewal [2].

In addition to synthesizing telomeres, telomerase may serve a more direct function in supporting the active proliferation of progenitor cells. This second function has been demonstrated most clearly in epidermis, an excellent tissue for studying progenitor cell biology and cellular differentiation. Within the epidermis, each hair is maintained by a dedicated organ, the hair follicle, which cycles among three states: a resting phase (telogen), an active phase (anagen), and a short regression phase (catagen) [6]. The initiation of a new anagen cycle requires the activation of a small number of multipotent stem cells that reside in a niche termed the bulge region [7–10]. These activated stem cells give rise to the newly generated portion of the anagen follicle, including cycling progenitor cells (matrix cells) that produce the cells comprising the growing hair shaft. Conditional expression of TERT in adult skin induces a rapid developmental transition in hair follicles from telogen to anagen by causing otherwise quiescent hair follicle stem cells to proliferate. Notably, this activity of TERT in facilitating anagen does not require the telomerase RNA component (TERC) and therefore is independent of TERT's role in synthesizing telomere repeats [3]. In contrast, an independent study found that the effects of TERT on keratinocytes colony formation and skin cancer required TERC, rendering the relationship between TERT's stem cell activation and telomere elongation functions less clear [4,11].

TERT's effects on hair follicle dynamics and stem cell biology closely resemble those of developmental pathways regulating hair follicle morphogenesis and cycling [6]. For example, by stabilizing the transcriptional activator beta-catenin, secreted Wnt glycoproteins transduce signals that are required for hair follicle development and differentiation, stem cell activation and stem cell maintenance [12–15]. Similarly, Shh and the components of its signaling pathway are important for hair follicle development [16,17] and are sufficient to initiate an anagen cycle [18]. Bmps are critical for follicle development, primarily by controlling hair follicle differentiation [19].

The similarities between the effects of these developmental regulators and those of conditional TERT overexpression suggest the possibility that TERT may act through one or more of these essential pathways. To gain critical new insights into the mechanisms by which TERT activates tissue stem cells, we engineered a system in which we can conditionally express a TERT point mutant (TERT^ci^) devoid of reverse transcriptase activity in mouse skin. This approach allows analysis of TERT functions in isolation from its catalytic action on telomeres and from any unknown polymerase activities that could occur in combination with other templates. We couple this mouse genetic approach with analysis of genome-wide transcriptional responses in vivo and rigorous statistical comparisons of the TERT-response signature with a large number of microarray gene sets. We find that TERT activates epidermal progenitor cells not through its reverse transcriptase function, but by controlling a Myc- and Wnt-related transcriptional program.

## Results

### TERT^ci^ Is a Catalytically Inactive Mutant of TERT

**Figure 1:**
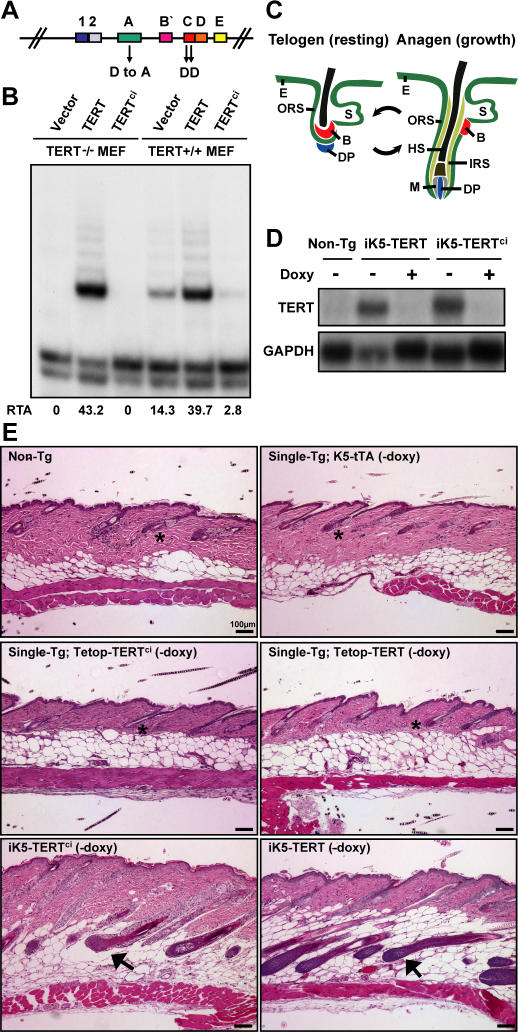
Conditional Expression of a Catalytically Inactive TERT Protein Induces the Anagen Phase of Hair Follicles (A) Diagram of TERT protein structure shows domains conserved among all RTs. Three conserved aspartates (shown) are required for RT function. D702 in motif A was mutated to alanine, to create the mouse TERT^ci^ allele. (B) Mouse TERT^ci^ fails to reconstitute telomerase activity when transduced into TERT^−/−^ MEFs and inhibits endogenous telomerase activity in TERT^+/+^ MEFs by TRAP. TRAP activities were quantified by measuring band intensities, and relative telomerase activity (RTA) is shown below each lane. (C) Hair follicles cycle between an active growth phase (anagen) and a resting phase (telogen). E, epidermis; ORS, outer root sheath; HS, hair shaft; M, matrix cells; DP, dermal papilla; IRS, inner root sheath; B, bulge stem cell niche; S, sebaceous gland. (D) Northern blot shows doxycycline-dependent suppression of TERT mRNA in dorsal skin from iK5-TERT and iK5-TERT^ci^ mice. (E) Induction of either TERT^ci^ or TERT mRNA in mouse epidermis initiates an anagen cycle between days 50–60. Doxycycline was withdrawn at day 10. Controls including non-transgenic, K5-tTA single transgenic, and tetop-TERT^ci^ and tetop-TERT single transgenic mice remained in telogen (asterisks). In contrast, iK5-TERT^ci^ and iK5-TERT mice are in anagen (black arrows). H&E, 100×.

To determine if TERT's RT activity is required to promote stem cell proliferation, we inactivated TERT RT function by introducing a point mutation in its active site. TERT's RT domain comprises seven sub-regions of homology conserved among all telomerases and RTs [20]. Within motifs A and C, three aspartate residues are absolutely conserved and essential for catalytic function [20–22]. The aspartate residue in motif A was changed to alanine by site-directed mutagenesis of cDNAs encoding either human TERT or mouse TERT, yielding the TERT^ci^ allele (for catalytically inactive) (Figure 1A). Mouse and human TERT^ci^ proteins lacked telomerase activity when expressed in TERT^−/−^ MEFs or primary human fibroblasts, respectively, whereas wild-type TERT proteins efficiently reconstituted telomerase enzyme function (Figures 1B and S1A). Consistent with this loss of RT function, human TERT^ci^ did not immortalize primary human fibroblasts, whereas wild-type TERT efficiently extended replicative lifespan and prevented replicative senescence (Figure S1B and S1C). TERT^ci^ proteins were efficiently expressed based on their ability to inhibit endogenous telomerase activity (Figures 1B and S1A) [20–22] and because TERT and TERT^ci^ protein levels were indistinguishable by Western blot (Figure S1D and S1E). Together, these data show that TERT^ci^ protein is catalytically inert and accumulates to levels similar to that of the wild-type protein.

### RT Function Is Not Required for TERT To Induce the Anagen Phase of the Hair Cycle

Based on these results showing that TERT^ci^ lacks RT function and cannot act on telomeres, we engineered tetracycline-inducible TERT^ci^ transgenic mice (see Text S1, Table S1, and Figures S2 and S3 for additional details). To achieve conditional regulation of either wild-type mouse TERT or mouse TERT^ci^ in skin, we crossed tetop-TERT^+^ or tetop-TERT^ci+^ mice to a transgenic line that expresses the tetracycline transactivator (tTA) under control of the keratin-5 (K5) promoter [23]. The K5 promoter directs expression in the basal layer of the interfollicular epidermis and in the outer root sheath of the hair follicle, including the bulge region in which hair follicle stem cells reside (Figure 1C) [10]. In this tetracycline-off configuration, double transgenic K5-tTA^+^; tetop-TERT^+^ mice and K5-tTA^+^; tetop-TERT^ci+^ mice were treated with doxycycline-drinking water throughout embryogenesis to suppress transgenic TERT expression, then switched to normal water between postnatal days 10–21 to induce TERT expression. We obtained RNA from skin biopsies from double transgenic mice (hereafter referred to as iK5-TERT and iK5-TERT^ci^) and analyzed TERT expression by Northern blot. TERT mRNA was readily detected in skin samples from iK5-TERT and iK5-TERT^ci^ mice off doxycycline, but not in skin from double transgenic mice maintained on doxycycline, from single transgenic mice or from non-transgenic controls (Figure 1D). Thus, TERT mRNA was efficiently induced in the skin of iK5-TERT and iK5-TERT^ci^ mice following withdrawal of doxycycline.

The hair follicle cycle is synchronized in mice for the first 60–70 days of life. The first post-natal anagen (growth phase) ends at approximately day 16, followed by the first telogen (resting phase). By day 28, the second anagen begins and is followed by a protracted telogen phase between days 40 and 60 [24] (Figure 1C). Conditional upregulation of TERT in skin initiates a premature anagen during this prolonged second telogen phase [3]. To determine if RT function is required for TERT to initiate a new round of the hair follicle cycle, we studied the hair follicle cycle in iK5-TERT mice and in iK5-TERT^ci^ mice during the second telogen phase. During this period (days 50–60), biopsies from iK5-TERT mice showed that 7/9 (78%) mice were in anagen, consistent with previous results [3]. Remarkably, conditional expression of TERT^ci^ induced anagen with equal efficiency compared to wild-type TERT. Histological analysis of skin biopsies showed that hair follicles were in anagen in 12/14 (86%) iK5-TERT^ci^ mice at days 50 to 60. In contrast, nearly all hair follicles from littermate control mice were in telogen during this period (25/27 control mice in telogen, *p* < 0.001) (Figure 1E and Table S2). These data show that RT activity is not required for induction of anagen when TERT is conditionally upregulated in mouse skin.

### TERT Promotes Hair Growth and Activates Hair Follicle Stem Cells Independent of RT Function

**Figure 2:**
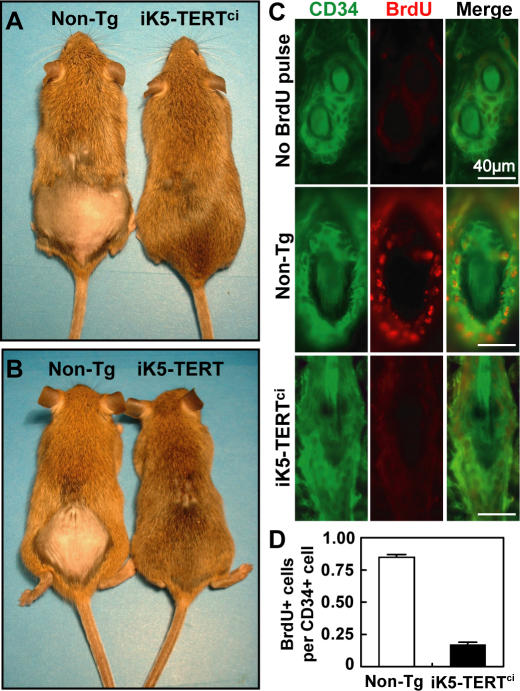
TERT^ci^ Promotes Hair Growth and Activates Hair Follicle Stem Cells (A–B) Mice were shaved at approximately day 50 and hair growth was assessed after 2 wk. Note pink skin in shaved areas of controls, consistent with a continued telogen phase even after 14 d. (C) Dorsal skin biopsies were taken after long-term BrdU chase, and BrdU label retention in the CD34^+^ bulge stem cell compartment was assessed for non-Tg mice (middle) and iK5-TERT^ci^ mice (bottom). A non-Tg mouse that did not receive BrdU serves as a negative control (top). Green, CD34; red, BrdU; 400×. (D) Quantification of the number of BrdU positive cells per CD34^+^ cell in the bulge region. Conditional expression of TERT^ci^ depleted BrdU label from label retaining cells in the hair follicle bulge region. A total of 1,075 CD34^+^ cells from 75 bulges of four non-Tg mice, and 724 CD34^+^ cells from 65 bulges of six iK5-TERT^ci^ mice were analyzed. Error bars represent standard error.

To determine if expression of wild-type TERT or TERT^ci^ in the K5 layer caused hair growth, doxycycline-drinking water was withdrawn from double transgenic mice at day 21 to allow TERT upregulation. Mice were then shaved in the second post-natal telogen at day 50 and followed for two weeks. The majority of control mice, including single transgenic mice and non-transgenic mice, showed no hair growth during this period (9/12 mice without hair growth). In marked contrast, nearly all iK5-TERT mice and iK5-TERT^ci^ mice showed efficient hair growth during this interval (6/6 iK5-TERT mice and 9/10 iK5-TERT^ci^ grew hair, *p* < 0.01 for both wild-type and mutant TERT versus controls) (Figure 2A and 2B, and Table S2). These data show that conditional upregulation of TERT in the K5 compartment of the hair follicle stimulates robust hair growth through a mechanism that does not require enzymatic function.

TERT initiates anagen and facilitates hair growth by inducing proliferation in otherwise quiescent hair follicle bulge stem cells. To determine if TERT's enzymatic activity is required for activating stem cells, we followed the proliferative status of stem cells in the hair follicle bulge region in iK5-TERT^ci^ mice and controls. Injection of bromodeoxyuridine (BrdU) at day 10 followed by a long chase period leads to BrdU retention in hair follicle stem cells as these cells withdraw from the cell cycle during postnatal development [7,10]. Label retaining cells (LRCs) in the bulge region express the stem cell marker CD34 [10,25,26]. These CD34^+^ cells in the hair follicle bulge include multipotent epidermal stem cells based on their ability to self-renew and to give rise to all epidermal lineages when engrafted in nude mice [9].

To test the ability of TERT^ci^ to activate bulge stem cells, litters of iK5-TERT^ci^ mice and appropriate control mice were injected with BrdU repeatedly at day 10, followed by removal of doxycycline. The prevalence of BrdU positive cells was assessed in the bulge region by double immunostaining for BrdU and the bulge stem cell marker CD34. LRCs were abundant in the bulge region from non-transgenic and single transgenic control mice. Eighty-five percent of CD34^+^ bulge stem cells were BrdU positive after a 40 to 50 day chase, a result in agreement with previous studies [3,27]. In contrast, only 17% of CD34^+^ cells stained positive for BrdU in iK5-TERT^ci^ mice, a reduction of 80% compared with controls (Figure 2C and 2D; *p* < 0.001 by Student's *t*-test). This marked reduction in BrdU label within the stem cell compartment caused by expression of TERT^ci^ was comparable to the effect seen with wild-type TERT [3]. Despite the loss of BrdU signal, staining for CD34 was similar in iK5-TERT^ci^ mice versus non-transgenic controls, indicating that CD34^+^ stem cells are retained with TERT expression. Together, these data show that RT activity is dispensable for TERT's effects in causing quiescent hair follicle stem cells to proliferate, in facilitating a developmental switch from telogen to anagen and in enabling hair growth.

### TERT Causes Hyperproliferation in the Basal Layer of the Interfollicular Skin

**Figure 3:**
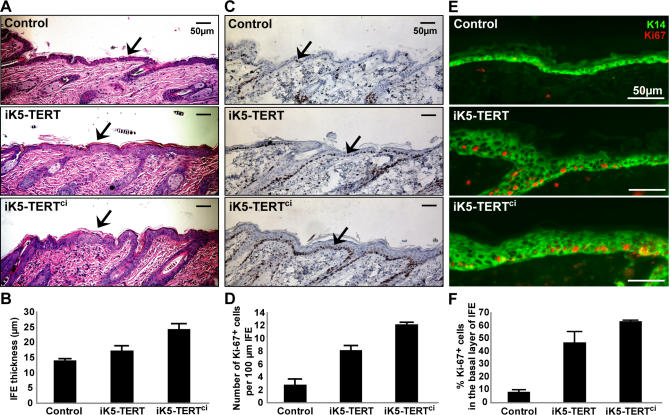
Both TERT and TERT^ci^ Enhance Proliferation of Interfollicular Epidermis in Mouse Skin (A) Expression of either TERT or TERT^ci^ increases thickness of interfollicular epidermis (IFE; black arrows). Dorsal skins were biopsied at days 55–60 and stained with H&E. Controls include non-transgenic and single-transgenic mice. (B) Quantification shows significant thickening of IFE in both iK5-TERT and iK5-TERT^ci^ (12 control, eight iK5-TERT, and 14 iK5-TERT^ci^ mice were analyzed. *p* < 0.00001 by one-way ANOVA and *p* < 0.05 by Student's *t*-test between control and iK5-TERT). (C,E) Expression of either TERT or TERT^ci^ increases proliferation in the basal layer of IFE. Abundance of Ki-67^+^ cells is increased in IFE of iK5-TERT and iK5-TERT^ci^ mice at days 55–60. Ki-67^+^ cells are stained by DAB (C) (black arrows), hematoxylin counterstain. Double immunostaining for Keratin 14 and Ki-67 (E). Green, K14; Red, Ki-67. (D,F) Quantification of Ki-67 staining shows significant increase in proliferation index. (D) Number of Ki-67^+^ cells per 100 μm. (F) Percentage of Ki-67^+^ cells within the basal layer of IFE (*p* < 0.0001 by one-way ANOVA and *p* < 0.01 by Student's *t*-test between control and iK5-TERT).

Although the skin, or interfollicular epidermis (IFE), is repaired by progeny of hair follicle bulge stem cells during wounding, renewal of the IFE under homeostatic conditions is maintained by long-lived stem cells or progenitor cells in the basal layer of the IFE [28,29]. To determine if TERT affects progenitor cells in this basal layer, we examined the IFE in detail. The IFE was significantly thickened in both iK5-TERT mice (17.43 μm, *p* < 0.05) and in iK5-TERT^ci^ mice (24.33 μm, *p* < 0.0001) versus controls (14.02 μm) (Figure 3A and 3B). To understand if the thickening of the IFE was due to increased proliferation in the basal layer, we measured the proliferation index using Ki-67 immunohistochemistry. The proliferation index was markedly elevated in both iK5-TERT mice (8.2 Ki-67^+^ cells per 100 μm, 46.5% Ki-67^+^ cells among basal cells of IFE, *p* < 0.01) and in iK5-TERT^ci^ mice (12.2 per 100 μm, 63.1% among basal IFE, *p* < 0.0005) versus controls (2.8 per 100 μm, 7.9% among basal IFE) (Figure 3C–3F). The larger effects of TERT^ci^ compared to wild-type TERT on IFE thickness and proliferation index are likely due to variables intrinsic to comparing transgenic founder lines, such as expression level and variegation differences (unpublished data). Interestingly, Keratin 14-positive layer of the IFE was markedly expanded in both iK5-TERT mice and iK5-TERT^ci^ mice, although Ki-67^+^ cells were mostly confined in the basal monolayer (Figure 3E). These data indicate that the effects of TERT overexpression on skin extend beyond activation of bulge stem cells and include stimulation of progenitors in the basal layer of IFE.

### Acute Withdrawal of TERT Causes a Rapid Change in Expression of Genes Involved in Epithelial Development, Signal Transduction, and Cytoskeleton/Adhesion

**Figure 4:**
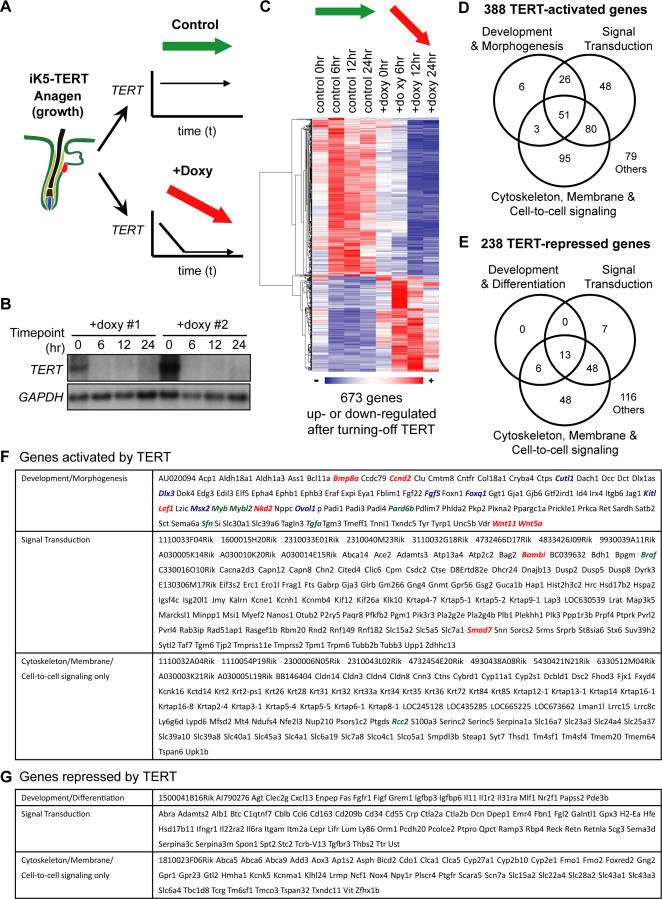
Acute Withdrawal of TERT Alters Expression of Genes Involved in Development, Signal Transduction, and Cell-to-Cell Signaling (A) In two cohorts of iK5-TERT mice used for microarrays, TERT was switched on postnatally, inducing anagen at day 60. In TERT ON, or control samples, iK5-TERT mice remained off doxycycline, and TERT expression was maintained (green arrow). In TERT OFF samples, iK5-TERT mice were injected with doxycycline at *t* = 0, acutely silencing TERT expression (red arrow). (B) Northern blots show rapid silencing of TERT mRNA after doxycycline injection in iK5-TERT mice. GAPDH serves as a loading control. (C) Clustered heat map of TERT-regulated gene expression profile (FDR < 0.05). Control = TERT ON; +Doxy = TERT OFF (D–E) Venn diagrams depicting the distribution of TERT-activated genes (D) or TERT-repressed genes (E) within each functional category. (F) List of TERT-activated genes analyzed in (D). Red, components of Bmp or Wnt pathways; green, cell-cycle–related genes; blue, genes having mouse epidermal phenotypes. Note that not all genes with known functions are color-coded. (G) List of TERT-repressed genes analyzed in (E).

Based on these data showing that TERT activates bulge stem cells, induces anagen, and stimulates proliferation in the IFE independent of catalytic function, we hypothesized that TERT acts in this context as a developmental regulator. We reasoned that we could identify putative pathways through which TERT acts by performing gene expression array experiments coupled with rigorous bioinformatic analyses. In designing these experiments, we leveraged several strengths of our system: (1) the ability to study the effects of TERT on the whole organ in vivo (2) temporal control of TERT with doxycycline enabling a study of dynamic gene expression changes over time and (3) a tetracycline-off configuration shown to result in rapid silencing in vivo [30]. To avoid the dramatic tissue changes associated with activating TERT and inducing anagen, we instead used iK5-TERT mice in which we allowed TERT to first induce anagen and hyper-proliferation of IFE. After induction of these changes, we then administered doxycycline to silence TERT expression, followed by a series of closely spaced serial biopsies. Because we were only interested in gene expression changes directly linked to TERT, we studied two cohorts of iK5-TERT mice, whose hair follicles were in TERT-induced anagen at age 60–65 days. In the TERT-off cohort, biopsies were obtained immediately before doxycycline treatment (*t* = 0), as well as 6, 12, and 24 hours after doxycycline injection to extinguish TERT expression. In parallel, age-matched iK5-TERT mice in the TERT-on cohort were injected with vehicle and biopsied at the same time points (Figure 4A). RNA was extracted from dorsal skin biopsies and used for Northern blot and for gene expression analyses using Affymetrix 430 2.0 arrays. TERT expression by Northern blot in the TERT-off cohort was efficiently suppressed by doxycycline injection within 6 hours (Figure 4B). This experimental design allows us to study the genome-wide response to acute withdrawal of TERT, while minimizing secondary gene expression changes caused by alterations in tissue architecture.

Significance analysis of microarrays (SAM) was applied to our time course microarray data to yield the genes differentially regulated in TERT-on and TERT-off samples (false discovery rate [FDR] < 0.05) [31]. Hierarchical clustering of these data revealed a total of 673 TERT-regulated genes; 418 genes were down-regulated in TERT-off samples (TERT-activated genes) and 255 genes were up-regulated in TERT-off samples (TERT-repressed genes) (Figure 4C). Unsupervised clustering of both samples and genes revealed that the *t* = 0 samples from TERT-off mice were more closely related to TERT-on mice, indicating that changes in TERT levels, rather than other variables, underlie these gene expression changes (Figure S4). Importantly, the expression of nearly all TERT-regulated genes was altered within 6–12 hours, kinetics consistent with a primary role for TERT in altering gene expression. Together, these data show that TERT down-regulation prompted a significant and acute gene expression change in mouse skin (Figure 4C and Table S3).

**Figure 5:**
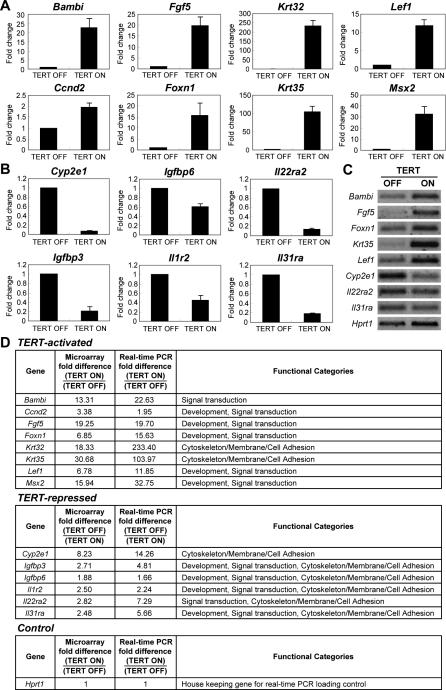
Validation of TERT-Regulated Genes by Quantitative RT-PCR (A–B) Representative TERT-activated genes (A) and TERT-repressed genes (B) associated with different functional categories were validated by real-time PCR. (TERT ON = Control 24-h timepoint; TERT OFF = 24 h after doxycycline treatment timepoint; n = 3. Error bars represent standard errors). (C) Semi-quantitative RT-PCR validation of some representative genes. (D) Detailed results of quantitative RT-PCR validation of TERT-regulated genes.

To begin to understand the patterns of gene expression changes, we first classified TERT-regulated genes based on functional annotation [32]. TERT-activated genes consisted of three major categories, genes involved in development/morphogenesis (22.2%, 86/388), signal transduction (52.8%, 205/388), and cytoskeleton/membrane/cell-to-cell signaling (59.0%, 229/388) (Figure 4D). A number of TERT-activated genes were prominent members of the Wnt, Shh and BMP pathways, signaling cascades critical for hair follicle development and cycling. These include *Bambi, Bmp8a, Ccnd2, Lef1, Nkd2, Smad7, Wnt5a,* and *Wnt11* (Figure 4F, colored in red). Other TERT-activated genes are not known to reside in these pathways, but have strong epidermal phenotypes in knockout or transgenic mice, including *Cutl1, Dlx3, Fgf5, Foxq1, Kitl, Msx2,* and *Ovol1* (Figure 4F, colored in blue). Compared to TERT-activated genes, fewer TERT-repressed genes were associated with development/differentiation (8.0%, 19/238) and signal transduction (28.6%, 68/238), but a similar number were associated with cytoskeleton/membrane/cell-to-cell signaling (48.3%, 115/238) (Figure 4E and 4G). To validate our microarray results, we performed quantitative RT-PCR on 14 target genes derived from our gene expression profiling experiments. These candidate genes were chosen to represent each of the three functional categories assigned for TERT-regulated genes. Each of the 14 TERT-activated and TERT-repressed genes studied showed strong regulation by TERT withdrawal at 24 hours by real-time RT-PCR, results in close agreement with data from our microarray analysis (Figure 5). These data show that TERT controls the expression of critical regulators involved in epithelial development, signal transduction and cytoskeleton/cell adhesion.

### Genes Regulated by TERT Are Chromosomally Clustered

**Figure 6:**
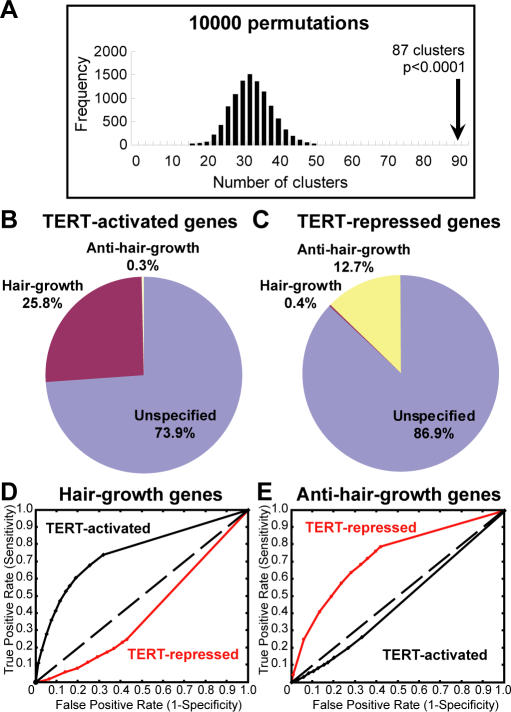
TERT Regulates a Concerted Transcriptional Program Overlapping Hair Growth and Anti–Hair Growth Genes in Normal Hair Cycling (A) Histogram showing the frequency of genes whose start sites clustered using a randomly generated gene list equal in size to the TERT-regulated gene set (10,000 permutations). TERT-regulated genes reside in 87 clusters (black arrow), whereas only 33 clustered genes are expected. (B) TERT-activated genes are highly enriched with hair growth pattern genes (100 hair growth genes; 1 anti–hair growth gene; 287 others). (C) TERT-repressed genes are highly enriched with anti–hair growth genes (30 anti–hair growth genes; 1 hair growth gene; 206 others). (D–E) ROC plots of various TERT-activated gene lists and TERT-repressed gene lists, generated by different FDR thresholds, in predicting hair growth pattern genes (D) or anti–hair growth pattern genes (E).

We noted that TERT-regulated genes were frequently members of multi-gene families whose transcriptional start sites are in close physical proximity. For example, TERT strongly activated expression of hair keratins, such as *Krt31* through *Krt36*. These genes likely arose through gene duplication and reside in the keratin locus on chromosome 11. It is becoming increasingly recognized that the genomic organization of coordinately regulated genes is non-random and that such genes are often chromosomally clustered in eukaryotes. Examples include genes regulated in a tissue-specific fashion and genes regulated as targets in specific signal transduction pathways [33,34]. Physical clustering of coordinately regulated genes may facilitate the organization of actively transcribed chromatin into specific nuclear domains [35]. To determine if TERT-regulated genes are commonly clustered on chromosomes, we compared the frequency of chromosomal clustering among TERT-regulated genes compared to equal numbers of randomly permuted genes. Among 586 TERT-regulated genes for which transcriptional start sites (TSS) could be assigned, 141 genes (24.1%) were within 100 kb of another gene, resulting in 87 chromosomal clusters (Table S4). Choosing the same number of genes within each chromosome at random and analyzing their TSS proximity for 10,000 iterations resulted in a mean of 33 chromosomal clusters, indicating that TERT-regulated genes were significantly clustered along chromosomes (Figure 6A, and Tables S4 and S5; *p* < 0.0001). Moreover, TERT-repressed genes were also chromosomally clustered (Table S5; *p* < 0.0001 for both). The dramatically enhanced clustering of TERT-regulated genes is consistent with TERT controlling a concerted transcriptional program.

### TERT Transcriptionally Controls a Physiological Program of Hair Growth and Anti–Hair Growth

If TERT does regulate a transcriptional program, we reasoned that it might be similar in part to the developmental program controlling natural hair follicle cycling. Gene expression analysis was previously performed on mouse skin during the synchronized waves of anagen and telogen to describe sets of genes that either were associated with hair growth, by peaking at anagen, or were negatively associated with hair growth, by rising at catagen or by declining in anagen [36]. To understand how hair growth or anti–hair growth genes relate to TERT-regulated genes, we systematically compared these data sets. Strikingly, hair growth genes were significantly enriched in our TERT-activated gene set (25.8%, 100/388), whereas anti-growth genes were nearly absent (0.3%, 1/388). Conversely, anti–hair growth genes were well represented in our TERT-repressed gene set (12.7%, 30/237), while hair growth genes were essentially absent (0.4%, 1/237) (Figure 6B and 6C and Table S6; *p* < 10^−27^ by Chi-square test). These findings show that TERT activates the expression of hair growth genes and inhibits the expression of anti–hair growth genes. Importantly, these gene expression changes are directly linked to changes in TERT levels and are not accounted for by alterations in the hair follicle cycle. Our gene expression analysis was intentionally performed in anagen over a brief 24-hour period, during which follicles remained in anagen based on histology.

To determine if the strong correlation between our data set and hair growth/anti-growth genes depended on statistical variables used to define TERT-regulated genes, we employed receiver operating characteristic (ROC) analysis, which allows the size of our TERT-regulated gene set to be systematically varied by altering the FDR cutoff in SAM. This approach creates unbiased lists of TERT-regulated genes of varying sizes and compares them to hair growth or anti-growth genes. Our results show that TERT-activated genes were always enriched in the hair growth gene data set, and TERT-repressed genes were always under-represented among hair growth genes, independent of statistical stringency in defining the collection of TERT-regulated genes (Figure 6D). Similarly, in the anti-growth gene list, TERT-repressed genes were always well represented, whereas TERT-activated genes were seldom seen regardless of parameters used to define the TERT-regulated gene list (Figure 6E). Together, these data show that TERT promotes epithelial proliferation through an intrinsic developmental program that coordinates the expression of hair growth and anti–hair growth genes.

### The Transcriptional Programs Controlled by TERT, Myc, and Wnt Are Highly Related

**Figure 7:**
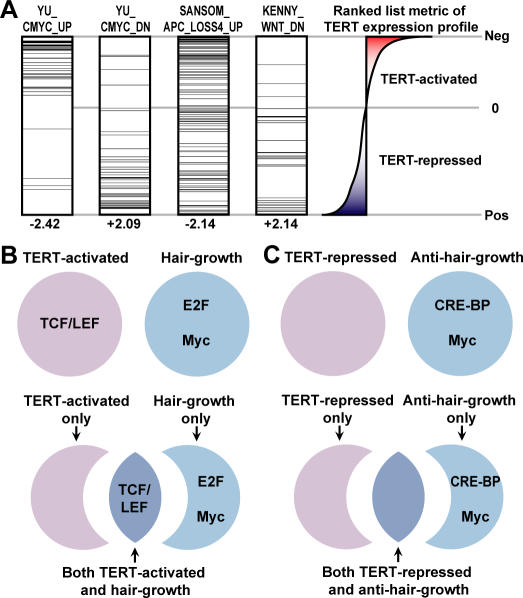
TERT-Regulated Genes Strongly Resemble Myc- and Wnt-Regulated Genes, and Are Enriched with TCF/LEF Binding Sites (A) Representative GSEA results show that Myc and Wnt gene sets are highly enriched in our TERT-regulated dataset. Within each MSigDB gene set, horizontal black bars represent genes matching the rank ordered TERT-regulated dataset. Gene set names are denoted on top of each box and normalized enrichment scores (NES) on bottom. Note that negative NES values indicate enrichment in TERT-activated genes, and positive values indicate enrichment in TERT-repressed genes. (B–C) Graphic representation of *cis*-regulatory motif enrichment in various groups of genes. TCF/LEF sites were enriched in TERT-activated genes; E2F and Myc binding sites were enriched in hair growth genes (B); and CRE-BP and Myc sites were enriched in anti–hair growth genes (C). Although TCF/LEF sites were not over-represented in the entire collection of hair growth genes, they were significantly enriched in the subset of hair growth genes activated by TERT.

Based on these results showing that TERT protein activates epidermal progenitor cells and regulates the expression of chromosomally clustered genes that strongly overlap with those controlling natural hair follicle cycling, we hypothesized that TERT protein is a component of specific developmental pathways. To identify these pathways, we used Gene Set Enrichment Analysis (GSEA), a powerful algorithm that allows a statistical comparison of our TERT-regulated gene dataset with 1134 curated gene sets derived from diverse experiments in the literature [37,38]. This approach requires that we reduce our TERT-regulated gene data set to a rank-ordered list, which is then queried by each individual gene set in the curated database. Strikingly, GSEA comparisons revealed strong connectivity between TERT and two pathways known to regulate progenitor cells, Myc and Wnt (Figure 7A and Table S7).

Six independent gene sets representing Myc-activated genes showed significant enrichment within TERT-activated genes. Similarly, four independent gene sets representing Wnt-activated genes were strongly enriched within TERT-activated genes (Table 1). As a further validation of these connections, Myc-repressed genes and Wnt-repressed genes were over-represented within TERT-repressed genes (Table 2). Importantly, Myc- or Wnt-repressed genes were never significantly enriched within TERT-activated genes, and conversely, Myc- or Wnt-activated genes were never over-represented within TERT-repressed genes, indicating that TERT, Myc and Wnt may regulate a similar pathway. Consistent with our findings that TERT enhances proliferation of epidermal progenitor cells (Figure 3C-3F), GSEA also identified significant similarity between TERT-regulated genes and cell cycle-related gene sets (Tables 1 and 2; for detailed methods, see Text S1).

**Table 1:**
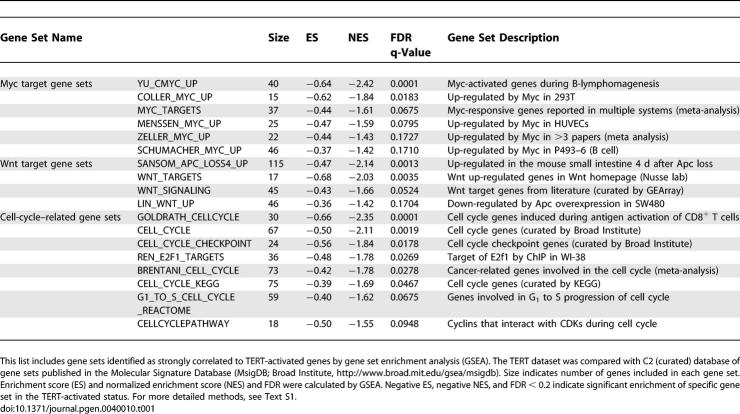
Gene Sets with Strong Correlations to TERT-Activated Genes by GSEA

**Table 2:**
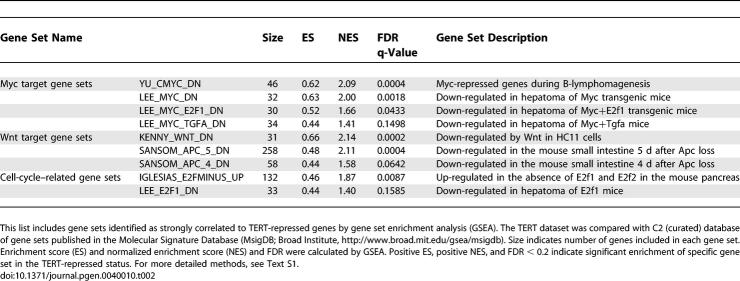
Gene Sets with Strong Correlations to TERT-Repressed Genes by GSEA

One particular strength of GSEA is its ability to allow comparisons across microarray platforms, species and cell types [39]. This advantage is well illustrated here because our TERT gene set derived from mouse skin matched Wnt pathway patterns seen with conditional deletion of the beta-catenin regulator Apc in mouse gastrointestinal tract and with overexpression of Apc in human colon cancer cells. Similarly, our data set matched Myc gene sets derived from B-cells and endothelial cells. Thus, GSEA allows the identification of fundamental patterns intrinsic to a pathway being studied. Together, these data show that TERT controls a transcriptional program that overlaps those regulated by Myc and Wnt, pathways crucial for development, stem cell regulation and cancer.

### TERT Activates Genes Harboring TCF/LEF Binding Sites

Coordinately regulated genes frequently share common *cis*-acting promoter elements, which serve as binding sites for sequence specific transcription factors in a signaling pathway. To identify promoter binding sites in common among TERT-regulated genes, we employed a GSEA algorithm that enables one to search for evolutionarily conserved transcription factor binding sites in particular sets of genes. We compared our rank ordered list of TERT-regulated genes with 783 sequence motif gene sets obtained from MSigDB, a database detailing which genes harbor conserved elements for each specific regulatory motif [40]. This approach identified several transcription factor binding sites as being significantly enriched in TERT-regulated genes. Remarkably, this evolutionarily conserved promoter binding site approach independently implicated the Myc and Wnt pathways. Myc binding sites and TCF/LEF binding sites, the elements through which Wnt signaling is mediated, were over-represented among TERT-repressed and TERT-activated genes, respectively (Table S8).

To independently validate these findings, we prospectively scanned the promoter regions of TERT-regulated genes using TRANSFAC consensus binding sequences for both Myc and TCF/LEF. We scanned the promoter regions surrounding the TSS of each gene for each transcription factor binding site motif and also incorporated information regarding the extent to which a specific promoter element is evolutionarily conserved (details in Text S1). Using this approach, we confirmed that TCF/LEF sites were highly enriched in TERT-activated genes, consistent with our preceding results (*p* < 0.05). Applying this methodology to the hair growth genes described above revealed that Myc and E2F sites were well represented in hair growth genes, while Myc and CRE-BP sites were enriched in the anti–hair growth gene data set (Figure 7B and 7C, and Table S9). Although the Wnt pathway serves a critical role in regulating the anagen phase of the hair follicle cycle, TCF/LEF sites were not statistically over-represented in the promoter regions of all hair growth genes as a group. Strikingly, TCF/LEF sites were highly enriched among the subgroup of hair growth genes regulated by TERT. Thus, TERT specifically regulates the subset of hair growth genes that contain TCF/LEF sites, providing mechanistic insight into how TERT induces anagen and facilitates epithelial proliferation. Together, these data show that the developmental program controlled by TERT strongly resembles the Myc and Wnt programs and suggest that TERT action is mediated through TCF/LEF promoter binding sites.

## Discussion

Telomerase is expressed in tissue progenitor cells and is upregulated in human cancers, where it supports cell viability and self-renewal by adding telomere repeats to chromosome ends. Our data show that TERT is a dual-function protein that acts in a telomere-independent manner to activate epidermal progenitor cells in the bulge region and in the IFE, and to promote a developmental transition from telogen to anagen resulting in hair growth. Its role in activating tissue progenitor cells does not require its catalytic function at telomeres, because TERT^ci^, a mutant lacking detectable RT function, exerts the same effects on tissue progenitor cells as does wild-type TERT. Instead, TERT acts to facilitate a rapid change in gene expression that overlaps the gene expression program of naturally cycling hair follicles. Strikingly, TERT-regulated genes share strong statistical similarity to genes regulated by Myc and Wnt. Together, our data strongly suggest that TERT acts as a developmental regulator via the Myc and Wnt signaling networks.

### TERT As a Developmental Regulator Controlling Gene Expression

Our results showing that TERT's catalytic function is not required for its ability to regulate epidermal progenitor cells indicate that TERT acts through novel mechanisms that do not require enzymatic action at telomere ends. The fact that TERT acutely causes profound changes in gene expression in mouse skin supports this idea and indicates that TERT is directly or indirectly associated with gene regulation. Interestingly, stable overexpression of TERT in cell culture, including human mammary epithelial cells, mouse ES cells, and MEFs also led to a specific transcriptional response [41–43]. Several aspects of our analyses indicate that the TERT signature defined here represents a coherent, coordinated genetic program. First, many of the genes controlled by TERT encode proteins with known regulatory functions in hair follicle development. These include components of the Wnt, Shh and BMP pathways as well as transcription factors such as the homeobox proteins Dlx3 and Msx2, among many others. Second, TERT-regulated genes show an elevated frequency of chromosomal clustering, most commonly as gene pairs, but sometimes as three genes or more with transcription start sites in close proximity. This non-random organization of genes along the chromosome is seen in worms, mice and humans, likely reflecting the fact that coordinate regulation of genes is facilitated by their physical location. Actively transcribed, clustered genes may loop into nuclear domains containing localized transcription machinery [33,35]. Thus, expression of TERT constitutes a signal that impinges on these gene clusters based on the *cis*-sequences in their promoter regions and/or their physical location in the nucleus. Third, the TERT gene signature shows remarkable overlap with the genes controlling the natural cycles of telogen and anagen. TERT-activated genes were specifically associated with growth genes, while TERT-repressed genes were invariably in the anti-growth category. Importantly, using rigorous ROC statistics, we found that this clear linkage between the TERT signature and the natural genetic program in cycling follicles is independent of the variables used to define the composition of the TERT signature. Thus, TERT controls a highly specific and organized program that coincides with many genes regulating normal hair follicle cycling.

### Convergence of TERT, Myc, and Wnt Pathways

Using GSEA, we found that the TERT signature is highly related to genetic programs controlled by Myc and by Wnt. This is particularly striking since Myc and Wnt represent two fundamental pathways regulating proliferation, differentiation and tissue progenitor cell function. Wnt is a secreted morphogen that initiates a signaling cascade leading to stabilization of beta-catenin, a protein that transactivates promoters bound by the transcription factors TCF or LEF1 [44]. The Wnt pathway is essential for hair follicle morphogenesis, for activation of hair follicle stem cells and for stem cell maintenance in the hair follicle bulge region. Interestingly, the effects of conditional beta-catenin activation in skin closely mimic those of TERT. Activation of a beta-catenin-estrogen receptor fusion protein in skin initiated a new anagen cycle [45]. Expression of a more active beta-catenin variant in skin also promoted anagen, but in addition caused the formation of new hair follicles and ultimately hair follicle tumors [46,47]. In normal cycling hairs, the beta-catenin pathway is activated as quiescent hair follicle stem cells enter cycle to become active progenitors, whereas suppression of Wnt signaling is thought to take place in the quiescent multipotent stem cells. The striking similarities between their gene expression programs and the fact that TERT expression phenocopies beta-catenin in skin suggest the possibility that TERT impinges upon the Wnt pathway (see Text S1 and Table S10 for additional details).

Myc is a potent oncogene that regulates gene expression by binding cognate DNA sequences as a heterodimer with a related protein Max [48]. Myc serves diverse functions in progenitor cells in vivo. Although Myc is required for proliferation in some cell contexts, it is essential for differentiation of hematopoietic stem cells into more committed progenitors [49]. In skin, Myc appears dispensable for epidermal stem cell function and normal epidermal differentiation [50]. However, overexpression of Myc in skin can enhance keratinocyte proliferation and skews differentiation toward a sebaceous gland fate [51,52]. Myc causes depletion of BrdU from LRCs in the IFE, but unlike conditional expression of either TERT or beta-catenin, Myc has not been reported to initiate a new anagen cycle [53]. However, Myc is already linked to the Wnt pathway by virtue of being an important transcriptional target of beta-catenin [54]. In fact, Myc mediates the hyperproliferative effects of Wnt activation, as demonstrated through conditional deletion of both Apc and Myc in the GI tract [55]. Overall, TERT overexpression recapitulates the effects of Myc in enhancing proliferation of epidermal progenitor cells in the interfollicular skin, but does not cause the sebaceous gland hyperplasia seen with Myc overexpression (see Text S1 and Figure S5 for additional details). Myc and TERT are also linked in that Myc is thought to regulate TERT gene transcription, although this connection is insufficient to explain the similarity in gene expression profiles identified here.

### *cis*-Elements Controlling TERT-Regulated Genes

The linkage among these pathways was uncovered through an independent analysis using GSEA to identify conserved elements in the promoter regions of TERT-regulated genes. E-boxes, the DNA motifs recognized by Myc/Max heterodimers, and TCF/LEF motifs, the sites through which beta-catenin acts, were enriched as conserved motifs in TERT-repressed genes and TERT-activated genes, respectively. In prospectively searching the promoter regions for these consensus sequences, we confirmed that TCF/LEF sites were significantly over-represented among TERT-regulated genes. E-boxes did not show an increased frequency in TERT promoter elements, which may represent an issue with the degeneracy of the short E-box sequence and limitations in locating relevant sites through this scanning approach. As further evidence that TERT activates genes containing TCF/LEF promoter sites, we found that in the hair growth gene data set as a whole, TCF/LEF sites were not significantly over-represented. In marked contrast, TCF/LEF sites were specifically enriched in the subset of hair growth genes controlled by TERT, providing independent evidence that TERT selectively activates genes with TCF/LEF sites.

### Telomerase Knockout Mice and Developmental Compensation

Our data suggest that TERT is an important developmental regulator in mouse skin, linking TERT to the Wnt and Myc networks, critical signaling pathways for epidermal development. However, no report of skin abnormalities has been reported so far from studies using TERT^−/−^ mice [56–58]. How then can our clear gain-of-function results be reconciled with the absence of a phenotype in first generation TERT^−/−^ mice? The simplest answer is that loss of TERT during embryonic development leads to adaptation or compensation, minimizing the deleterious effects of TERT deletion. Developmental compensation is well documented for germline knockouts of critical regulatory genes involved in cell cycle and gene regulation. In the case of the Rb family [59], D-type Cyclins [60], E-type Cyclins [61], CDK4/6 [62] and FoxO transcription factors [63,64], deletion of a single gene is compensated by remaining family members. Although functional overlap by family members is a common mechanism of compensation, it has been suggested that compensation may occur more commonly through adaptation of non-homologous components of a signaling network [65]. Thus, the Wnt and Myc signaling networks may adapt to minimize the loss of TERT during embryonic development, even though TERT is an active component of these networks in wild-type mice. With this idea in mind, alternative loss-of-function strategies may reveal a role for TERT in epidermal development or in other processes.

### Defining Non-Canonical Pathways for Telomerase Action

Our findings here showing that TERT enhances epithelial proliferation by regulating a gene expression program with strong similarity to Myc and Wnt provide critical new insight into how TERT acts in pathways distinct from its catalytic role at telomeres. These results are consistent with the findings of others showing that TERT acts in transformation and the DNA damage response independent of its ability to lengthen telomeres [66,67]. Our results showing that RT activity is not required in this new pathway implicate a novel aspect of TERT protein function whereby TERT is a component of a signaling pathway that impinges on the Wnt or Myc networks. TERT may act at any point along the Wnt signaling axis: by altering Wnt expression, by facilitating transduction of Wnt signals through Dishevelled, Gsk3, Axin or Apc; or by enhancing transcription initiation by beta-catenin at TCF/LEF promoter elements. The similarity between the genetic programs controlled by TERT and Myc may be explained if TERT regulated Myc levels, or if it affected the delicate balance of chromatin modification controlled by Myc and Max family members at E boxes. These data provide important justification for investigating the biochemistry and cell biology of TERT, areas that have remained largely refractory to analysis due to the very low abundance of TERT in cells and tissues. Continued investigation in these areas will likely provide new insights into how TERT functions at telomeres and in a non-canonical fashion as a developmental regulator in epithelial tissues.

## Materials and Methods

### Generation of transgenic mice.

Tetracycline-regulated i-TERT transgenic mice were generated as previously described [3]. To engineer a catalytically inactive TERT allele (TERT^ci^), the conserved Asp at position 702 in RT motif A was changed to Ala by PCR using the mouse TERT cDNA as a template. A 400 bp MseI-BstEII DNA fragment encoding the D702A mutation was subcloned back into the full-length mouse TERT cDNA and verified by DNA sequencing. The TERT^ci^ cDNA was then placed under control of a tetracycline-inducible promoter by subcloning a 3.5kb EcoRI fragment into the EcoRV site of pTRE2 (Clontech) by blunt-ended ligation, yielding tetop-TERT^ci^. Prokaryotic sequences were removed and the purified DNA fragment was injected into pronuclei of FVB/N fertilized zygotes. Founder mice were screened by PCR and Southern blot. Tetop-TERT^ci^ transgenic mice were intercrossed with actin-rtTA^+^ mice or keratin-5-tTA^+^ mice to generate double transgenic mice for characterization. For iK5-TERT and iK5-TERT^ci^ mice, doxycycline (5 μg/ml) was administered in drinking water throughout development until post-natal day 10–21 to continually suppress transgenic TERT expression during development and allow TERT upregulation to occur postnatally. All mice were treated in accordance with AAALAC approved guidelines at Stanford University.

### Telomeric repeat amplification protocol (TRAP) and Northern blot analysis.

For TRAP assays, protein was extracted from cells or skin tissues in CHAPS lysis buffer, and a standard TRAP reaction was performed using 50–500 ng of protein extract (TRAPeze, Chemicon). Relative telomerase activity of equally loaded samples was measured by using gel analysis tools from ImageJ 1.37v software (http://rsb.info.nih.gov/ij). For Northern blots, RNA was isolated from skin tissues or from cells using TRIZOL (Invitrogen). Five μg of total RNA was fractionated on a 0.8% denaturing agarose gel, transferred to Hybond-N membrane (Amersham), and hybridized with TERT or GAPDH ^32^P-labeled DNA probes using ULTRAhyb (Ambion).

### Histology and immunohistochemistry.

Dorsal skin biopsies were obtained from mice under anesthesia, fixed overnight in 10% formalin and embedded in paraffin. Five μm sections were stained with hematoxylin and eosin (H&E) for microscopic analysis. For immunohistochemistry, paraffin sections were treated with antigen retrieval reagent (Vector Labs), 3% H_2_O_2_ and a biotin block (Dako). Ki-67^+^ cells were detected using a mouse monoclonal anti-Ki-67 antibody (BD Pharmingen, 1:1000), a biotinylated anti-mouse IgG antibody (MOM kit, Vector Labs), and either by Cy3-streptavidin (Jackson Immunoresearch, 1:800), or by HRP-Streptavidin (Dako), DAB^+^ substrate chromogen (Dako), and hematoxylin counterstaining. Keratin 14 positive cells were detected using a rabbit anti-mouse-K14 antibody (Covance, 1:500) and FITC-anti-rabbit IgG (Jackson Immunoresearch, 1:200).

### Analysis of label retaining cells.

LRC analysis was done as previously described with minor modifications [3,9,27]. Briefly, to label hair follicle stem cells, 10-day-old mice were injected with 50 μg/g body weight of BrdU every 12 hours for four injections to mark proliferating epidermal keratinocytes. Mice were then switched from doxycycline water to normal water to allow upregulation of the TERT transgene. Skin samples were obtained from the mice after a chase period of 40–50 days. Immunofluorescence was performed on frozen sections to visualize label retaining cells and CD34^+^ hair follicle bulge stem cells, using antibodies to CD34 (BD, 1:100), BrdU (Abcam, 1:500), FITC- and Cy3-conjugated secondary antibodies (Jackson ImmunoResearch).

### Gene expression profiling.

To acutely extinguish expression of transgenic TERT, doxycycline (8 μg/g body weight) was injected intraperitoneally to iK5-TERT mice in the TERT-off cohort at *t* = 0, whereas iK5-TERT mice in the TERT-on cohort were injected with PBS at *t* = 0. Dorsal skin biopsies (3 cm^2^) were immediately soaked in 10 volume of RNAlater (Ambion) overnight at 4 °C to capture an accurate in vivo gene expression profile. The subcutaneous fat layer was surgically removed later, prior to RNA extraction. Total RNA was extracted using TRIZOL reagent (Invitrogen), and purified by RNeasy column (Qiagen). mRNA from purified total RNA was amplified, labeled and fragmented using MessageAmp II-Biotin Enhanced Kit (Ambion). Labeled aRNA was hybridized to Mouse Genome 430 2.0 arrays (Affymetrix) and scanned at the Protein and Nucleic Acid Biotechnology Facility at Stanford. Probe intensity was calculated by Affymetrix MAS software, and the resulting CEL files were analyzed by dChip software; arrays were normalized based on invariant sets, and gene expression levels were calculated using the PM/MM difference model [68]. Additional bioinformatics analyses are detailed in Text S1.

### Quantitative RT-PCR.

Total RNA was extracted from dorsal skin biopsies of TERT-ON and TERT-OFF mice 24 hrs after injection with PBS or doxycycline, respectively, using TRIZOL (Invitrogen), and subsequently purified by RNeasy column (Qiagen). Equal amount of RNA for each cohort was reverse transcribed by Superscript II Reverse Transcriptase (Invitrogen) with random primers. Primer pairs for PCR quantification were designed by PerlPrimer version 1.1.14 to span intron/exon boundaries and at least one primer to bridge an exon/exon junction. Sequences of primers are available upon request. For semi-quantitative PCR, optimal amplification cycles for individual genes were determined by testing various cycles, and Hprt1 was used as an internal control. For real-time PCR, amounts of transcripts were quantified by using an iCycler iQ real-time PCR detection system (Bio-Rad) and QuantiTect SYBR Green PCR kit (Qiagen). Differences between TERT OFF and TERT ON samples were calculated based on the 2^−ΔΔCt^ method using Hprt1 as an internal control.

## Supporting Information

Figure S1 Expression of a Catalytically Inactive TERT Protein in Cells (A) Human TERT^ci^ lacks telomerase activity by TRAP assay in BJ fibroblasts and inhibits endogenous telomerase activity in HeLa cells.
(B) Wild-type human TERT, but not TERT^ci^, immortalizes BJ primary human fibroblasts.
(C) BJ cells transduced with human TERT^ci^ or vector show senescence-associated beta-galactosidase staining at 104 days after transduction (40× magnification).
(D) Wild-type human TERT and human TERT^ci^ proteins accumulate to similar levels when transduced in HeLa cells and BJ fibroblasts by anti-TERT Western blot. Tubulin was used as a loading control.
(E) Wild-type mouse Flag-TERT and mouse Flag-TERT^ci^ proteins accumulate to similar levels in transduced MEFs by IP-Western analysis with anti-Flag antibody.
(726 KB PDF)

Figure S2 Expression of TERT^ci^ Does Not Affect Telomere Stability in MEFs (A) Structure of tetop-TERT^ci^ transgene.
(B) Northern blot shows strong induction of TERT^ci^ mRNA in i-TERT^ci^ MEFs treated with 2 μg/ml doxycycline. GAPDH was used as a loading control.
(C,D) Metaphase chromosome analysis from MEFs in (B) shows no evidence of telomere dysfunction with induction of TERT^ci^, including an absence of both signal-free ends and chromosome fusions.
(473 KB PDF)

Figure S3 Expression of TERT^ci^ Inhibits Telomerase Activity in Transgenic Mice Telomerase activity is increased in anagen skin of iK5-TERT mice, but is decreased in anagen skin of iK5-TERT^ci^ mice in the absence of doxycycline as shown by TRAP assay.
(396 KB PDF)

Figure S4 Acute Withdrawal of TERT Induces Rapid Changes in Gene Expression Unsupervised clustering of both genes and samples, shows that *t* = 0 samples from TERT-ON (control, green arrow) and TERT-OFF (+doxy, red arrow) samples cluster together, reflecting their close relatedness because TERT remains on in both samples. However, with injection of doxycycline to silence TERT expression in the TERT-OFF samples, subsequent time points diverge rapidly. Gene expression profiles from 6-, 12-, and 24-h time points from TERT-ON remain most related to the 0-h time points, whereas gene expression profiles from 6-, 12-, and 24-h time points from TERT-OFF samples cluster separately. These results are consistent with acute withdrawal of TERT driving the changes in gene expression.
(194 KB PDF)

Figure S5 Expression of TERT or TERT^ci^ Does Not Alter the Size of Sebaceous Glands Sebaceous gland (red) size is not changed in dorsal skin of male iK5-TERT or iK5-TERT^ci^ mice versus male non-transgenic mice. Blue, hematoxylin; Red, Oil Red O.
(420 KB PDF)

Table S1 Summary of Cytogenetics in i-TERT ^ci^ MEFs (27 KB DOC)

Table S2 Summary of Anagen Induction and Hair Growth in iK5-TERT and iK5-TERT^ci^ Mice (31 KB DOC)

Table S3 List of TERT-Regulated Genes with FDR < 0.05 by SAM Analysis (830 KB DOC)

Table S4 List of Chromosomal Gene Clusters among TERT-Regulated Genes (115 KB DOC)

Table S5 Summary of Chromosomal Clustering Scanning Results (29 KB DOC)

Table S6 Raw Data for Hair Growth/Anti–Hair Growth Comparison Analysis (1.4 MB DOC)

Table S7 Associations Found by GSEA between Wnt/Myc Pathway/Cell-Cycling Gene Sets and TERT Gene Signature Are Statistically Significant (52 KB DOC)

Table S8 TERT Gene Expression Signature Is Enriched with Evolutionarily Conserved TCF/LEF, Myc (E-box), E2F, and CRE-BP Motif–Containing Gene Sets (53 KB DOC)

Table S9 Detailed Results of *cis*-Regulatory Motif Enrichment Analysis (219 KB DOC)

Table S10 TERT and TERT^ci^ Do Not Induce Hair Follicle Neomorphogenesis (28 KB DOC)

Text S1 Supporting Results, Materials and Methods, and References (88 KB DOC)

### Accession Numbers

The Entrez Gene (http://www.ncbi.nlm.nih.gov/sites/entrez?db=gene) GeneIDs of genes described in this paper are: *Apc* (11789), *Bambi* (68010), *Bmp8a* (12163), *Ccnd2* (12444), *Cd34* (12490), *Cutl1* (13047), *Cyp2e1* (13106), *Dishevelled* (13542), *Dlx3* (13393), *Fgf5* (14176), *Foxn1* (15218), *Foxq1* (15220), *Gsk3* (56637), *Hprt1* (15452), *Igfbp3* (16009), *Igfbp6* (16012), *Il1r2* (16178), *Il22ra2* (237310), *Il31ra* (218624), *Ki-67* (4288), *Kitl* (17311), *Krt14* (16664), *Krt31* (16660), *Krt32* (16670), *Krt35* (53617), *Krt36* (16673), *Krt5* (110308), *Lef1* (16842), *Msx2* (17702), *Myc* (17869), *Nkd2* (72293), *Ovol1* (18426), *Smad7* (17131), *Terc* (21748), *Tert* (21752), *Wnt11* (22411), *Wnt5a* (22418).

The data discussed in this publication have been deposited in The National Center for Biotechnology Information Gene Expression Omnibus (GEO; http://www.ncbi.nlm.nih.gov/geo) and are accessible through GEO Series accession number GSE9725.
