# Supplementary material for: TERT Promotes Epithelial Proliferation through Transcriptional Control of a Myc- and Wnt-Related Developmental Program
Source: PLoS Genet. 2008 Jan 18;4(1):e10. doi: 10.1371/journal.pgen.0040010 (PMC2211538; doi:10.1371/journal.pgen.0040010)
Supplement: Supplementary file 6 [file pgen.0040010.sd001.doc]

**Supporting Results**

*Tetracycline-inducible TERT^ci^ does not impair telomere stability, but reduces telomerase activity in the mouse skin*

Based on results indicating that TERT^ci^ lacks RT activity, we generated tetracycline-inducible TERT^ci^ mice (Fig. S2A). To characterize regulation of the TERT^ci^ transgene in cell culture, tetop-TERT^ci^+ mice were intercrossed with actin-rtTA+ mice to generate actin-rtTA+ tetop-TERT^ci^+ (i-TERT^ci^) double transgenic MEFs. Double transgenic MEFs showed strict doxycycline-dependent induction of the TERT^ci^ transgene by Northern blots (Fig. S2B). To rule out an unanticipated effect on telomere stability from TERT^ci^ overexpression, metaphase chromosomes were prepared and telomere-fluorescence in situ hybridization (FISH) was performed. Chromosomal end-to-end fusions, a hallmark of dysfunctional telomeres, were not detected in any metaphases (Fig. S2C,D, Table S1). These data indicate that TERT^ci^ is regulated in a doxycycline-dependent manner and that acute overexpression of this TERT mutant does not impair telomere stability.

Endogenous telomerase activity is strongly associated with the anagen hair cycle phase, a period of intense progenitor cell activity, in both human and mouse [1,2]. To examine the effect of ectopic expression of TERT and TERT^ci^ on endogenous telomerase activity in mouse skin, we obtained protein extracts from anagen skin samples from mice at postnatal day 100-120, and measured telomerase activity by telomere repeat amplification protocol (TRAP). While upregulation of wild-type TERT increased telomerase activity in skin from iK5-TERT mice off doxycycline, expresion of TERT^ci^ in skin from iK5-TERT^ci^ mouse reduced TRAP activity (Fig. S3). These data show that TERT and TERT^ci^ are each induced after withdrawal of doxycycline, and that catalytically inert TERT^ci^ reduces telomerase activity in the skin of double transgenic mice.

*Expression of TERT or TERT^ci^ in mouse skin alters neither follicle density nor sebaceous gland size*

Enhanced Wnt/β-catenin signaling caused by overexpression of β-catenin in mouse skin leads to stem cell activation and promotion of the anagen phase of the hair follicle cycle [3]. Deletion of the amino-terminal domain that targets the protein for degradation yields a β-catenin variant with enhanced activity (∆N-β-catenin). Expression of ∆N-β-catenin in mouse skin activates stem cells, and promotes anagen, but also causes generation of new follicles, leading to an increase in follicle density [4,5]. To understand if TERT overexpression led to follicle neomorphogenesis, we compared hair follicle density by counting hair follicles per unit length of epidermis in ~d60 mouse skin samples from iK5-TERT and iK5-TERT^ci^ mice versus non-transgenic controls. We detected no difference in follicle density among these genotypes (Table S10). Thus, the effect of TERT and TERT^ci^ overexpression in inducing anagen closely resembles that of transient β-catenin overexpression [3], but does not fully copy the effects of overexpressing the very potent ∆N-β-catenin variant.

Overexpression of Myc in the basal layer of the skin causes epidermal stem cell mobilization and sebaceous gland hyperplasia in mouse skin [6,7]. We found that overexpression of TERT or TERT^ci^ in interfollicular skin enhanced keratinocyte proliferation, a phenotype similar to that seen with transgenic expression of Myc. To further compare the TERT and Myc phenotypes, we asked whether overexpression of TERT or TERT^ci^ caused expansion of cells in the sebaceous gland compartment in mouse skin. We therefore stained dorsal skin sections from male iK5-TERT, iK5-TERT^ci^ and non-transgenic control mice, at ~d60, with Oil Red O (Fig. S5). We detected no difference in sebaceous gland size among these genotypes in both anagen hair follicles and telogen hair follicles. Therefore, TERT overexpression recapitulates the effects of Myc in enhancing proliferation of epidermal progenitor cells in the interfollicular skin [2,8], but does not cause the sebaceous gland hyperplasia seen with Myc overexpression.

**Supporting Material and Methods**

***Cell culture and retroviral transduction***

Human foreskin fibroblasts (BJ cells) were cultured as previously described [9]. HeLa cells and mouse embryonic fibroblasts (MEFs) were cultured in Dulbecco’s Modified Eagle’s Medium (Invitrogen) with 10% bovine growth serum (HyClone) and 1% Penicillin/Streptomycin in a humidified chamber at 37^o^C under 5% CO_2_. A fragment of the human TERT cDNA was mutated through PCR and the mutated fragment was subcloned back into the TERT cDNA to generate the human TERT^ci^ allele (D712A). Human TERT^ci^ and wild-type TERT cDNA sequences were cloned into the pLPC retroviral plasmid (gift of S. Lowe) and verified by DNA sequencing. Mouse TERT^ci^ and wild-type TERT proteins were expressed using pBABE retrovirus. To detect mouse TERT proteins, a Flag epitope tag was introduced at the amino-terminus of the mouse TERT^ci^ and TERT sequences, which were then cloned into the pMGIB retroviral plasmid (gift of G. Nolan). Retroviral supernatants generated by transfecting one of these retroviral plasmids into 293T cells, along with plasmids encoding gag, pol and VSV-g, were then used to infect HeLA cells, BJ fibroblasts or MEFs at high efficiency, followed by drug selection.

*BJ fibroblast growth curve and senescence associated beta-galactosidase staining*

BJ cells at pd45 were retrovirally transduced using pLPC, pLPC-hTERT, or pLPC-hTERT^ci^ as described above, and were selected by 2μg/ml Puromycin for one week. Cells were counted and 7x10^5^ cells were plated to the 10 cm dish every 3.5 days. Population doublings were calculated by log_2_([number cells harvested]/[number cells plated]), and plotted against time (days). For senescence associated beta-galactosidase staining, cells were washed with PBS, fixed with fixing solution (2% formaldehyde, 0.2% glutaraldehyde in PBS) at RT for 5 min, washed with PBS again, and then stained with staining solution (5mM K_3_Fe(CN)_6_, 5mM K_4_Fe(CN)_6_, 2mM MgCl_2_, 150mM NaCl, 30mM Citric acid/phosphate buffer pH 6.0, 1mg/ml X-gal, in water) at 37ºC for overnight.

***Western blot analysis***

For Western blot analysis, human TERT protein was detected using affinity-purified antibodies, raised against an N-terminal TERT protein fragment expressed as a maltose binding protein fusion in bacteria. Specific antibodies were affinity purified on covalently cross-linked GST-TERT antigen columns. Flag-tagged mouse TERT proteins were detected by immunoprecipitation-Western blot using anti-Flag monoclonal antibodies (Sigma).

*Telomere fluorescence in situ hybridization*

Mouse embryonic fibroblasts were harvested after culture in the presence of 0.1 µg/ml colcemid (KaryoMAX, Invitrogen) for 1 hr, treated with hypotonic KCl for 15 min at 37 ^o^C, and fixed in cold methanol-acetic acid. Quantitative fluorescence in situ hybridization on metaphase chromosomes using Cy-3 labeled (CCCTAA)_3_ PNA probe was performed as previously described [10]. Digital images were captured with a SPOT RT 2.3.0 CCD camera (Diagnostic Instruments) on an Eclipse E800 microscope (Nikon) using SPOT Advanced software. Telomere intensities were analyzed using TFL-Telo V.2.1 (gift of P. Lansdorp). At least 21 metaphases (>3360 telomeres) were analyzed per MEF culture to determine the number of non-detectable telomeres (signal-free ends) and chromosomal end-to-end fusions. Signal-free ends were determined by Cy-3 photos taken with maximum gain.

*Significant analysis and hierarchical clustering of microarray results*

Genes with statistically significant differential expression levels (FDR<0.05) were detected by Significance Analysis of Microarray (SAM) software, using time-course, two class (unpaired) response type summarized by signed area with t-statistics, and 10000 permutations to obtain FDR [11]. Genes with FDR of <0.05 were again filtered by choosing genes that have less than 1.5 fold difference in gene expression levels between 0hr controls and 0hr doxy-treated samples, yielding 673 genes for subsequent analyses. Hierarchical clustering of gene expression profile was done by dChip software either with both ‘cluster samples’ and ‘cluster genes’ options (Fig. S4) or with ‘cluster genes’ option only (Fig. 4C). For hierarchical clustering and generation of heat maps, rows were standardized by subtracting mean and divided by standard deviation; distances between genes were calculated by correlation metric, and linkage was established by centroid method.

*Chromosomal clustering analysis*

Chromosomal gene clustering was examined by measuring the distance between transcriptional start sites (TSS) of neighboring genes in each gene list. Genes less than 100kb apart in their TSS were regarded as chromosomally clustered. Significance of chromosomal clustering results was assessed by calculating the probability of having the same number of clusters in 10000 randomly-generated same-sized gene lists. We also employed different distance windows, ranging from 25kb to 500kb, for chromosomal clustering analysis of all TERT-regulated genes, TERT-activated genes, and TERT-repressed genes, and consistently observed significant clustering (p<0.005) from above set of genes with distance criteria ranging from 25kb to 350kb (data not shown), further validating our findings.

*Functional genomic analyses of TERT-regulated genes*

To explore functional annotations of TERT-regulated genes, The Database for Annotation, Visualization and Integrated Discovery (DAVID 2007) was used (<http://david.abcc.ncifcrf.gov/>). List of genes were uploaded to DAVID as Affymetrix probe IDs, and the *Mus musculus* genome was used as background. To understand the response to acute TERT withdrawal, each detailed GO term was assigned to one of three categories: development, morphogenesis, and differentiation; signal transduction; or cytoskeleton, membrane and cell-to-cell signaling.

For cross-comparison of TERT-regulated genes with genes involved in hair cycling, we compared our data set to a comprehensive time-course gene expression profiling study that was previously performed on natural hair follicle cycling in mouse [12]. Lists of hair-growth pattern genes (clusters 1-8, 887 genes) and anti-hair growth pattern genes (clusters 16-24, 770 genes) were obtained from their supplementary material. Gene identifiers of hair-growth genes and anti-hair-growth genes, as well as our TERT-activated genes and TERT-repressed genes, were converted to HUGO universal gene symbols using Netaffx batch conversion tool (Affymetrix, <http://www.affymetrix.com/analysis/>) to facilitate the comparison (Table S6).

For ROC analysis, TERT-regulated gene lists of different size were generated by varying FDR thresholds. Each FDR threshold-specific TERT-regulated gene list was tested to see how well they predicted membership in the hair-growth or anti-hair-growth test set. Sensitivity (TP/TP+FN) and Specificity (TN/FP+TN) of TERT-activated genes and TERT-repressed genes in detecting hair-growth or anti-hair-growth pattern genes from the whole mouse genome were calculated and plotted as True positive rate (Sensitivity) versus False positive rate (1-Specificity) on ROC chart. For example, in ROC of ‘TERT-activated’ for ‘hair-growth genes’, TP=[# genes in both ‘TERT-activated’ and ‘hair-growth’ groups], TN=[# genes neither in ‘TERT-activated’ nor ‘hair-growth’ group], FP=[# genes in ‘TERT-activated’ but not in ‘hair-growth’ groups], FN=[# genes not in ‘TERT-activated’ but in ‘hair-growth’ group].

*GSEA analysis*

For GSEA, we needed to rank order our TERT data set, which consists of two time courses, one experimental and one control. Given the complexity of the data set, we rank ordered our genes using SAM’s time course, two class (unpaired) response type by signed area algorithm. As alternative methods of ranking the genes, we used two other methods within the GSEA program: (1) Pearson correlation matrix with continuous phenotype labels and (2) Signal2Noise matrix with categorical phenotype labels (<http://www.broad.mit.edu/gsea/>) [13]. The TERT-data set was compared with C2 (curated) and C3 (motif) database of gene sets published in the Molecular Signature Database (MsigDB, Broad Institute). Curated gene sets (C2 database) that were enriched in down-regulated (negative) phenotype at FDR of <0.2 were assigned as strongly correlated to TERT-activated genes, and those enriched in up-regulated (positive) phenotype were assigned as correlated to TERT-repressed genes. Among the strongly correlated gene sets found by GSEA, Myc and Wnt-related gene sets were discovered as significantly associated with our TERT data set, based on the fact that multiple independent gene sets with matching directions were strongly connected with TERT-regulated gene expression profile (p<0.03, Table S7). Cell cycle related gene sets were also significantly enriched in the TERT data set (p<0.001, Table S7). Importantly, numerous gene sets related to other pathways were not found to be enriched in the TERT data set (K-Ras, EGF, TGFbeta, MAPK), further evidence for the specific association of Wnt and Myc with TERT (Table S7). GSEA analysis with motif gene sets (C3 database) was performed to discover candidate over-represented motifs in TERT-regulated gene expression profile for further research [14]. We noticed enrichment of TCF/LEF, Myc, CRE-BP and E2F motifs in our TERT-regulated gene expression profile, and further investigated this by direct motif scans described in the next section. The GSEA results shown in the manuscript used the rank list generated by SAM, however, our GSEA results were reproducible regardless of which rank-ordering protocols were employed.

*cis-regulatory motif scan*

To determine if a specific transcription factor binding site (TFBS) was enriched in a set of genes, genomic DNA sequences of [-5kb,+1kb] and [-2kb,+2kb] with respect to the TSS of each gene in the list were scanned using selected TRANSFAC consensus binding sequences. In addition to this simple sequence scanning, we also restricted our analyses by filtering the results based on cross-species conservation. We defined conserved TFBS as one that resides within the top 7% most conserved genomic regions. Conservation was evaluated through a PhastCons score (for mouse mm8, Feb. 2006). PhastCons score is a conservation score generated using a phylogenetic HMM from multiple species alignments (16 genomic assemblies from Human to Fugu were aligned to mouse mm8) [15], and is browsable through the UCSC genome browser (http://genome.ucsc.edu/). We then submitted both raw scanning results and conservation-filtered results for further statistical evaluations. Results from these four different scans ([-5kb,+1kb] or [-2kb,+2kb] / Raw hits or Conservation-filtered hits) were compared with the number of TFBS found in 10000 randomly picked genes; one-tailed two proportion z-statistics test was performed to determine the significance of the result. Results with statistical significance were reported in Table S9. Overall, results were consistent regardless of the scanning and scoring methods employed, showing that our result is free from biases that can be introduced by choosing specific scoring or scanning methods.

**Supporting References**

1. Ramirez RD, Wright WE, Shay JW, Taylor RS (1997) Telomerase activity concentrates in the mitotically active segments of human hair follicles. J Invest Dermatol 108: 113-117.

2. Sarin KY, Cheung P, Gilison D, Lee E, Tennen RI, et al. (2005) Conditional telomerase induction causes proliferation of hair follicle stem cells. Nature 436: 1048-1052.

3. Van Mater D, Kolligs FT, Dlugosz AA, Fearon ER (2003) Transient activation of beta -catenin signaling in cutaneous keratinocytes is sufficient to trigger the active growth phase of the hair cycle in mice. Genes Dev 17: 1219-1224.

4. Gat U, DasGupta R, Degenstein L, Fuchs E (1998) De Novo hair follicle morphogenesis and hair tumors in mice expressing a truncated beta-catenin in skin. Cell 95: 605-614.

5. Lo Celso C, Prowse DM, Watt FM (2004) Transient activation of beta-catenin signalling in adult mouse epidermis is sufficient to induce new hair follicles but continuous activation is required to maintain hair follicle tumours. Development 131: 1787-1799.

6. Waikel RL, Kawachi Y, Waikel PA, Wang XJ, Roop DR (2001) Deregulated expression of c-Myc depletes epidermal stem cells. Nat Genet 28: 165-168.

7. Arnold I, Watt FM (2001) c-Myc activation in transgenic mouse epidermis results in mobilization of stem cells and differentiation of their progeny. Curr Biol 11: 558-568.

8. Flores I, Cayuela ML, Blasco MA (2005) Effects of telomerase and telomere length on epidermal stem cell behavior. Science 309: 1253-1256.

9. Middleman EJ, Choi J, Venteicher AS, Cheung P, Artandi SE (2006) Regulation of cellular immortalization and steady-state levels of the telomerase reverse transcriptase through its carboxy-terminal domain. Mol Cell Biol 26: 2146-2159.

10. Lansdorp PM, Verwoerd NP, van de Rijke FM, Dragowska V, Little MT, et al. (1996) Heterogeneity in telomere length of human chromosomes. Hum Mol Genet 5: 685-691.

11. Tusher VG, Tibshirani R, Chu G (2001) Significance analysis of microarrays applied to the ionizing radiation response. Proc Natl Acad Sci U S A 98: 5116-5121.

12. Lin KK, Chudova D, Hatfield GW, Smyth P, Andersen B (2004) Identification of hair cycle-associated genes from time-course gene expression profile data by using replicate variance. Proc Natl Acad Sci U S A 101: 15955-15960.

13. Subramanian A, Tamayo P, Mootha VK, Mukherjee S, Ebert BL, et al. (2005) Gene set enrichment analysis: a knowledge-based approach for interpreting genome-wide expression profiles. Proc Natl Acad Sci U S A 102: 15545-15550.

14. Xie X, Lu J, Kulbokas EJ, Golub TR, Mootha V, et al. (2005) Systematic discovery of regulatory motifs in human promoters and 3' UTRs by comparison of several mammals. Nature 434: 338-345.

15. Siepel A, Bejerano G, Pedersen JS, Hinrichs AS, Hou M, et al. (2005) Evolutionarily conserved elements in vertebrate, insect, worm, and yeast genomes. Genome Res 15: 1034-1050.
