# Supplementary material for: TERT Promotes Epithelial Proliferation through Transcriptional Control of a Myc- and Wnt-Related Developmental Program
Source: PLoS Genet. 2008 Jan 18;4(1):e10. doi: 10.1371/journal.pgen.0040010 (PMC2211538; doi:10.1371/journal.pgen.0040010)
Supplement: Supplementary file 7 [file pgen.0040010.st001.doc]

**Table S1. Summary of cytogenetics in i-TERT^ci^ MEFs**

| Genotype | No. MEFs culture | No. metaphases | Chromosomal  end-to-end fusions  per metaphase | Signal free ends per metaphase |
| --- | --- | --- | --- | --- |
| Control ^*^ | 2 | 50 | 0 | 0 |
| i-TERT^ci^ (+doxy) | 2 | 43 | 0 | 0 |

* Control consists of one Actin-rtTA (+doxy) and one Non-Tg (+doxy).
