# Supplementary material for: TERT Promotes Epithelial Proliferation through Transcriptional Control of a Myc- and Wnt-Related Developmental Program
Source: PLoS Genet. 2008 Jan 18;4(1):e10. doi: 10.1371/journal.pgen.0040010 (PMC2211538; doi:10.1371/journal.pgen.0040010)
Supplement: Supplementary file 8 [file pgen.0040010.st002.doc]

**Table S2. Summary of anagen induction and hair growth in iK5-TERT and iK5-TERT^ci^** mice

| Genotype | Doxycycline treatment | Anagen induction ^*^ | Hair growth promotion ^¶^ |
| --- | --- | --- | --- |
| Control | - | 2/27 ^†^ | 3/12 ^\|\|^ |
|  | + | 1/13 | 0/12 |
| iK5-TERT | - | 7/9 ^‡^ | 6/6 ^**^ |
|  | + | 0/6 | 0/6 |
| iK5-TERT^ci^ | - | 12/14 ^§^ | 9/10 ^††^ |
|  | + | 0/4 | 0/4 |

* Hair follicle phase was analyzed between D50~60 in dorsal skin.

† Anagen induction control consists of Non-Tg, K5-tTA, tetop-TERT and tetop-TERT^ci^ mice.

‡ Chi-square analysis iK5-TERT (-doxy) vs. control (-doxy): p<0.001

§ Chi-square analysis iK5-TERT^ci^ (-doxy) vs. control (-doxy): p<0.001

¶ Mice were shaved at D50~56 and hair growth was documented after two weeks.

|| Hair growth controls include Non-Tg, K5-tTA, tetop-TERT and tetop-TERT^ci^ mice.

** Chi-square analysis iK5-TERT (-doxy) vs. controls (-doxy): p<0.01

†† Chi-square analysis iK5-TERT^ci^ (-doxy) vs. controls (-doxy): p<0.01
