# Supplementary material for: TERT Promotes Epithelial Proliferation through Transcriptional Control of a Myc- and Wnt-Related Developmental Program
Source: PLoS Genet. 2008 Jan 18;4(1):e10. doi: 10.1371/journal.pgen.0040010 (PMC2211538; doi:10.1371/journal.pgen.0040010)
Supplement: Supplementary file 9 [file pgen.0040010.st003.doc]

**Table** S3. List of TERT-regulated genes with FDR<0.05 by SAM analysis

| **A. Down-regulated (TERT-activated) genes with FDR<0.05** | | | |
| --- | --- | --- | --- |
| **Probe Set ID** | **Gene Symbol** | **Score(d)** | **FDR (%)** |
| 1420672_at | Kcne1 | -5.5743 | 4.6487276 |
| 1455877_a_at | Nanos1 | -5.5031 | 4.6487276 |
| 1435154_at | LOC245128 | -5.3291 | 4.6487276 |
| 1436239_at | Slc5a5 | -5.0118 | 4.6487276 |
| 1434099_at | Ppargc1a | -4.919 | 4.6487276 |
| 1460336_at | Ppargc1a | -4.7804 | 4.6487276 |
| 1434502_x_at | Slc4a1 | -4.6144 | 4.6487276 |
| 1444487_at | Lrat | -4.6114 | 4.6487276 |
| 1438883_at | Fgf5 | -4.5501 | 4.6487276 |
| 1437259_at | --- | -4.5447 | 4.6487276 |
| 1421856_at | S100a3 | -4.5101 | 4.6487276 |
| 1421345_at | Lrat | -4.4496 | 4.6487276 |
| 1427211_at | Krtap8-1 | -4.4469 | 4.6487276 |
| 1419767_at | Padi3 | -4.4447 | 4.6487276 |
| 1437702_at | Tgm6 | -4.4339 | 4.6487276 |
| 1419840_at | Krt72 | -4.3493 | 4.6487276 |
| 1434100_x_at | Ppargc1a | -4.3465 | 4.6487276 |
| 1448485_at | Ggt1 | -4.327 | 4.6487276 |
| 1427154_at | Krt2 | -4.3214 | 4.6487276 |
| 1436055_at | Lrrc15 | -4.314 | 4.6487276 |
| 1420409_at | Krt35 | -4.3059 | 4.6487276 |
| 1455188_at | Ephb1 | -4.2997 | 4.6487276 |
| 1429835_at | 2310033E01Rik | -4.2911 | 4.6487276 |
| 1419323_at | Padi1 | -4.268 | 4.6487276 |
| 1458354_x_at | Krt28 | -4.2266 | 4.6487276 |
| 1425400_a_at | Cited4 | -4.183 | 4.6487276 |
| 1429896_at | 5830408B19Rik | -4.1492 | 4.6487276 |
| 1441133_at | Stx6 | -4.1487 | 4.6487276 |
| 1449559_at | Msx2 | -4.1418 | 4.6487276 |
| 1453009_at | Cpm | -4.1146 | 4.6487276 |
| 1425916_at | Capn8 | -4.1104 | 4.6487276 |
| 1436160_at | Krt26 | -4.0859 | 4.6487276 |
| 1460616_at | Slco4c1 | -4.0851 | 4.6487276 |
| 1429413_at | Cpm | -4.0832 | 4.6487276 |
| 1453511_at | 2310007B03Rik | -4.079 | 4.6487276 |
| 1450698_at | Dusp2 | -4.0527 | 4.6487276 |
| 1460185_at | Krt85 | -4.0259 | 4.6487276 |
| 1451424_at | Gabrp | -3.9862 | 4.6487276 |
| 1437870_at | Slco4c1 | -3.9739 | 4.6487276 |
| 1439100_s_at | --- | -3.9728 | 4.6487276 |
| 1429913_at | Kcnk16 | -3.9706 | 4.6487276 |
| 1457424_at | Eya1 | -3.9658 | 4.6487276 |
| 1444566_at | Dnajb13 | -3.9621 | 4.6487276 |
| 1438886_at | Heyl | -3.9544 | 4.6487276 |
| 1425138_at | Guca1b | -3.9489 | 4.6487276 |
| 1422790_at | Nppc | -3.9271 | 4.6487276 |
| 1420728_at | Krt32 | -3.9161 | 4.6487276 |
| 1436359_at | --- | -3.9135 | 4.6487276 |
| 1415978_at | Tubb3 | -3.8978 | 4.6487276 |
| 1434651_a_at | Cldn3 | -3.89 | 4.6487276 |
| 1424247_at | Erc1 | -3.8873 | 4.6487276 |
| 1416464_at | Slc4a1 | -3.8771 | 4.6487276 |
| 1419154_at | Tmprss2 | -3.8491 | 4.6487276 |
| 1453523_at | A030006P16Rik | -3.8141 | 4.6487276 |
| 1422037_at | Dlx3 | -3.814 | 4.6487276 |
| 1418966_a_at | Dcbld1 | -3.7966 | 4.6487276 |
| 1436398_at | --- | -3.7926 | 4.6487276 |
| 1446487_at | LOC435727 | -3.7819 | 4.6487276 |
| 1420686_at | Cryba4 | -3.7796 | 4.6487276 |
| 1428595_at | Slc6a19 | -3.7671 | 4.6487276 |
| 1424824_at | Slain1 | -3.7605 | 4.6487276 |
| 1429960_at | 4930438A08Rik | -3.7593 | 4.6487276 |
| 1455442_at | Slc6a19 | -3.7455 | 4.6487276 |
| 1447502_at | Cmah | -3.7425 | 4.6487276 |
| 1425611_a_at | Cutl1 | -3.7399 | 4.6487276 |
| 1419184_a_at | Fhl2 | -3.737 | 4.6487276 |
| 1434683_at | Cutl1 | -3.7256 | 4.6487276 |
| 1416239_at | Ass1 | -3.7053 | 4.6487276 |
| 1419507_at | Krtap15 | -3.704 | 4.6487276 |
| 1417160_s_at | Expi | -3.6967 | 4.6487276 |
| 1421594_a_at | Sytl2 | -3.6922 | 4.6487276 |
| 1442257_at | Cdh6 | -3.6796 | 4.6487276 |
| 1457102_at | A030001D16Rik | -3.6764 | 4.6487276 |
| 1438768_at | LOC665225 /// LOC669601 /// LOC676250 | -3.6761 | 4.6487276 |
| 1440086_at | Rnf182 | -3.6759 | 4.6487276 |
| 1416124_at | Ccnd2 | -3.6744 | 4.6487276 |
| 1448119_at | Bpgm | -3.6741 | 4.6487276 |
| 1415864_at | Bpgm | -3.6729 | 4.6487276 |
| 1452326_at | Atp2c2 | -3.6722 | 4.6487276 |
| 1457967_at | A030003K21Rik | -3.6663 | 4.6487276 |
| 1460569_x_at | Cldn3 | -3.6647 | 4.6487276 |
| 1451701_x_at | Cldn3 | -3.6589 | 4.6487276 |
| 1444491_at | --- | -3.6579 | 4.6487276 |
| 1423753_at | Bambi | -3.6558 | 4.6487276 |
| 1450774_at | Ly6g6d | -3.6516 | 4.6487276 |
| 1428573_at | Chn2 | -3.6504 | 4.6487276 |
| 1456883_at | Stox1 | -3.6482 | 4.6487276 |
| 1441991_at | BC039632 | -3.6449 | 4.6487276 |
| 1435551_at | Fhod3 | -3.6448 | 4.6487276 |
| 1418773_at | Fads3 | -3.6396 | 4.6487276 |
| 1439947_at | Cyp11a1 | -3.6386 | 4.6487276 |
| 1456147_at | St8sia6 | -3.637 | 4.6487276 |
| 1438894_at | --- | -3.6342 | 4.6487276 |
| 1439793_at | Gja3 | -3.6301 | 4.6487276 |
| 1440874_at | Slco5a1 | -3.6263 | 4.6487276 |
| 1425163_at | AI661453 | -3.624 | 4.6487276 |
| 1448566_at | Slc40a1 | -3.6227 | 4.6487276 |
| 1447018_at | --- | -3.6216 | 4.6487276 |
| 1418805_at | Sct | -3.6182 | 4.6487276 |
| 1435910_at | Fads3 | -3.6163 | 4.6487276 |
| 1447933_at | Kif26a /// LOC668303 /// LOC676226 | -3.6092 | 4.6487276 |
| 1436080_at | AW011738 | -3.6071 | 4.6487276 |
| 1450860_at | Lap3 | -3.6053 | 4.6487276 |
| 1424938_at | Steap1 | -3.6037 | 4.6487276 |
| 1456395_at | Ppargc1a | -3.5964 | 4.6487276 |
| 1440150_at | Tgm3 | -3.5861 | 4.6487276 |
| 1459967_at | --- | -3.5854 | 4.6487276 |
| 1443326_at | --- | -3.5789 | 4.6487276 |
| 1421299_a_at | Lef1 | -3.5665 | 4.6487276 |
| 1445028_at | Prkca | -3.5659 | 4.6487276 |
| 1447886_at | 0610040B09Rik | -3.5608 | 4.6487276 |
| 1433988_s_at | C230098O21Rik | -3.5603 | 4.6487276 |
| 1431165_at | A030009A09Rik | -3.5602 | 4.6487276 |
| 1427395_a_at | Aldh1a3 | -3.5506 | 4.6487276 |
| 1456440_s_at | St8sia6 | -3.5473 | 4.6487276 |
| 1422090_a_at | Pfkfb2 | -3.5461 | 4.6487276 |
| 1451532_s_at | Steap1 | -3.5459 | 4.6487276 |
| 1439109_at | Ccdc68 | -3.5455 | 4.6487276 |
| 1449387_at | Krt33a | -3.5453 | 4.6487276 |
| 1431171_at | D730001G18Rik | -3.5405 | 4.6487276 |
| 1438481_at | Prpf4 | -3.5394 | 4.6487276 |
| 1454969_at | Lypd6 | -3.5326 | 4.6487276 |
| 1420345_at | Cldn14 | -3.5322 | 4.6487276 |
| 1426633_s_at | Kctd14 | -3.5226 | 4.6487276 |
| 1420467_at | Psors1c2 | -3.5222 | 4.6487276 |
| 1436873_at | 4732454E20Rik | -3.5207 | 4.6487276 |
| 1435994_at | Kcnh1 | -3.5202 | 4.6487276 |
| 1439409_x_at | Tyrp1 | -3.5193 | 4.6487276 |
| 1437458_x_at | Clu | -3.5132 | 4.6487276 |
| 1443043_at | Otop2 | -3.5086 | 4.6487276 |
| 1424647_at | Gabrp | -3.4962 | 4.6487276 |
| 1460412_at | 1600015H20Rik | -3.4918 | 4.6487276 |
| 1456878_at | AI646023 | -3.4904 | 4.6487276 |
| 1422760_at | Padi4 /// LOC670543 | -3.4889 | 4.6487276 |
| 1449440_at | Lpin3 | -3.4806 | 4.6487276 |
| 1435110_at | Unc5b | -3.4759 | 4.6487276 |
| 1429944_at | Tchhl1 | -3.4737 | 4.6487276 |
| 1421943_at | Tgfa | -3.4728 | 4.6487276 |
| 1460296_a_at | Fgf22 | -3.4708 | 4.6487276 |
| 1454806_at | D12Ertd553e | -3.4679 | 4.6487276 |
| 1438658_a_at | Edg3 | -3.4607 | 4.6487276 |
| 1426332_a_at | Cldn3 | -3.4584 | 4.6487276 |
| 1449077_at | Eraf | -3.4545 | 4.6487276 |
| 1423174_a_at | Pard6b | -3.4539 | 4.6487276 |
| 1454709_at | Tmem64 | -3.4529 | 4.6487276 |
| 1440916_at | 2510049J12Rik | -3.4528 | 4.6487276 |
| 1454849_x_at | Clu | -3.4519 | 4.6487276 |
| 1440840_at | D630004K10Rik | -3.4503 | 4.6487276 |
| 1420695_at | Dach1 | -3.4471 | 4.6487276 |
| 1453247_at | Zfp618 | -3.4412 | 4.6487276 |
| 1439041_at | Slc39a10 | -3.4343 | 4.6487276 |
| 1442425_at | A030014E15Rik | -3.4292 | 4.6487276 |
| 1417929_at | Slc7a8 | -3.4215 | 4.6487276 |
| 1450508_at | Foxn1 | -3.4203 | 4.6487276 |
| 1425040_at | Cybrd1 | -3.4188 | 4.6487276 |
| 1448482_at | Slc39a8 | -3.4169 | 4.6487276 |
| 1437558_at | B130021B11Rik | -3.4149 | 4.6487276 |
| 1458871_at | Tmprss11e | -3.4101 | 4.6487276 |
| 1450540_x_at | Krtap5-1 | -3.4095 | 4.6487276 |
| 1448397_at | Gjb6 | -3.409 | 4.6487276 |
| 1434600_at | Tjp2 | -3.4081 | 4.6487276 |
| 1423175_s_at | Pard6b | -3.4055 | 4.6487276 |
| 1424730_a_at | Slc15a2 | -3.4046 | 4.6487276 |
| 1421589_at | Krt31 | -3.4009 | 4.6487276 |
| 1450535_at | Krtap12-1 | -3.3989 | 4.6487276 |
| 1437751_at | Ppargc1a | -3.3982 | 4.6487276 |
| 1424962_at | Tm4sf4 | -3.3979 | 4.6487276 |
| 1420452_at | Krtap5-2 | -3.3978 | 4.6487276 |
| 1426152_a_at | Kitl | -3.3873 | 4.6487276 |
| 1417837_at | Phlda2 | -3.3849 | 4.6487276 |
| 1422523_at | Si | -3.3797 | 4.6487276 |
| 1425837_a_at | Ccrn4l | -3.3786 | 4.6487276 |
| 1453192_at | 3110032G18Rik | -3.3724 | 4.6487276 |
| 1435415_x_at | Marcksl1 | -3.3656 | 4.6487276 |
| 1427719_s_at | Krt2-ps1 /// Krt82 | -3.3646 | 4.6487276 |
| 1453214_at | Lrrc15 | -3.3631 | 4.6487276 |
| 1430731_at | Krtap2-4 | -3.3617 | 4.6487276 |
| 1440487_at | Dcc | -3.3614 | 4.6487276 |
| 1433877_at | 4732473B16Rik | -3.3608 | 4.6487276 |
| 1429796_at | Kalrn | -3.357 | 4.6487276 |
| 1456210_at | 5430407P10Rik | -3.3566 | 4.6487276 |
| 1422292_at | A030005K14Rik | -3.3545 | 4.6487276 |
| 1422786_at | Slc30a1 | -3.3521 | 4.6487276 |
| 1454632_at | 6330442E10Rik | -3.3513 | 4.6487276 |
| 1419029_at | Ero1l | -3.3492 | 4.6487276 |
| 1417656_at | Mybl2 | -3.3488 | 4.6487276 |
| 1436791_at | Wnt5a | -3.3434 | 4.6487276 |
| 1430728_at | Krtap5-5 | -3.3413 | 4.6487276 |
| 1450772_at | Wnt11 | -3.34 | 4.6487276 |
| 1418129_at | Dhcr24 | -3.3392 | 4.6487276 |
| 1427751_a_at | Krt36 | -3.3385 | 4.6487276 |
| 1418626_a_at | Clu | -3.3328 | 4.6487276 |
| 1445913_at | A730090H04Rik | -3.3271 | 4.6487276 |
| 1449963_at | 2310040M23Rik | -3.3265 | 4.6487276 |
| 1423860_at | Ptgds | -3.324 | 4.6487276 |
| 1417061_at | Slc40a1 | -3.3219 | 4.6487276 |
| 1436548_at | 1810012P15Rik | -3.3216 | 4.6487276 |
| 1448612_at | Sfn | -3.3184 | 4.6487276 |
| 1451753_at | Plxna2 | -3.3157 | 4.6487276 |
| 1437403_at | E130306M17Rik | -3.3153 | 4.6487276 |
| 1439625_at | Krtap5-5 | -3.3145 | 4.6487276 |
| 1416563_at | Ctps | -3.311 | 4.6487276 |
| 1450683_at | Tagln3 | -3.3102 | 4.6487276 |
| 1438989_s_at | B130021B11Rik | -3.2994 | 4.6487276 |
| 1435206_at | Slc24a4 | -3.2985 | 4.6487276 |
| 1454991_at | Slc7a1 | -3.2981 | 4.6487276 |
| 1456593_at | Sytl2 | -3.2953 | 4.6487276 |
| 1458006_at | Cpm | -3.2922 | 4.6487276 |
| 1438261_at | Cited4 | -3.2902 | 4.6487276 |
| 1415861_at | Tyrp1 | -3.2888 | 4.6487276 |
| 1437689_x_at | Clu | -3.2878 | 4.6487276 |
| 1422733_at | Fjx1 | -3.2871 | 4.6487276 |
| 1419030_at | Ero1l | -3.2855 | 4.6487276 |
| 1449291_a_at | Dcbld1 | -3.2799 | 4.6487276 |
| 1459760_at | Ndufs4 | -3.2731 | 4.6487276 |
| 1423948_at | Bag2 | -3.2723 | 4.6487276 |
| 1422717_at | Acp1 | -3.2682 | 4.6487276 |
| 1443771_x_at | Smad7 | -3.264 | 4.6487276 |
| 1419358_at | Sorcs2 | -3.2621 | 4.6487276 |
| 1437893_at | Plb1 /// BC033606 /// LOC665270 | -3.2579 | 4.6487276 |
| 1428958_at | Paqr8 | -3.2441 | 4.6487276 |
| 1420599_at | 2300006N05Rik /// LOC670472 /// LOC670482 | -3.2436 | 4.6487276 |
| 1415801_at | Gja1 | -3.2387 | 4.6487276 |
| 1422983_at | Itgb6 | -3.2328 | 4.6487276 |
| 1435981_at | --- | -3.2315 | 4.6487276 |
| 1454866_s_at | Clic6 | -3.2311 | 4.6487276 |
| 1428384_at | D4Bwg0951e | -3.2268 | 4.6487276 |
| 1436115_at | Gm266 | -3.2243 | 4.6487276 |
| 1447669_s_at | Gng4 | -3.2213 | 4.6487276 |
| 1451513_x_at | Serpina1a /// Serpina1b | -3.2182 | 4.6487276 |
| 1427118_at | 5430421N21Rik | -3.2181 | 4.6487276 |
| 1450194_a_at | Myb | -3.2143 | 4.6487276 |
| 1444999_at | C77651 | -3.2128 | 4.6487276 |
| 1446791_at | Pi15 | -3.2128 | 4.6487276 |
| 1418028_at | Dct | -3.2065 | 4.6487276 |
| 1421680_at | A030005L19Rik | -3.2057 | 4.6487276 |
| 1425872_at | Krtap6-1 | -3.2021 | 4.6487276 |
| 1419539_at | Irx4 | -3.1969 | 4.6487276 |
| 1426911_at | Dsc2 | -3.1943 | 4.6487276 |
| 1425350_a_at | Myef2 | -3.1921 | 4.6487276 |
| 1428007_at | Krtap13-1 | -3.1867 | 4.6487276 |
| 1424680_at | BB146404 | -3.1853 | 4.6487276 |
| 1447808_s_at | Slc15a2 | -3.1851 | 4.6487276 |
| 1437306_at | C130092O11Rik | -3.1849 | 4.6487276 |
| 1451859_at | Krtap6-1 | -3.181 | 4.6487276 |
| 1424229_at | Dyrk3 | -3.1807 | 4.6487276 |
| 1449170_at | Piwil2 | -3.1759 | 4.6487276 |
| 1418989_at | Ctse | -3.1693 | 4.6487276 |
| 1421928_at | Epha4 | -3.169 | 4.6487276 |
| 1452226_at | Rcc2 | -3.1682 | 4.6487276 |
| 1449970_at | Capn12 | -3.1669 | 4.6487276 |
| 1423859_a_at | Ptgds | -3.1662 | 4.6487276 |
| 1449324_at | Ero1l | -3.1654 | 4.6487276 |
| 1418714_at | Dusp8 | -3.1623 | 4.6487276 |
| 1423721_at | Tpm1 | -3.1565 | 4.6487276 |
| 1417750_a_at | Slc25a37 | -3.1518 | 4.6487276 |
| 1450958_at | Tm4sf1 | -3.1453 | 4.6487276 |
| 1426955_at | Col18a1 | -3.1443 | 4.6487276 |
| 1428223_at | Mfsd2 | -3.1442 | 4.6487276 |
| 1448818_at | Wnt5a | -3.1432 | 4.6487276 |
| 1424614_at | Frag1 | -3.1423 | 4.6487276 |
| 1418283_at | Cldn4 | -3.1351 | 4.6487276 |
| 1424615_at | Frag1 | -3.1346 | 4.6487276 |
| 1434382_at | Serinc2 | -3.1326 | 4.6487276 |
| 1444306_at | Msi1 | -3.1319 | 4.6487276 |
| 1457843_at | Lypd6 | -3.1305 | 4.6487276 |
| 1451895_a_at | Dhcr24 | -3.1265 | 4.6487276 |
| 1428677_at | Wdr73 | -3.1257 | 4.6487276 |
| 1420415_at | AY026312 | -3.1254 | 4.6487276 |
| 1418742_at | Krt34 | -3.1246 | 4.6487276 |
| 1452517_at | Plekhh1 | -3.1243 | 4.6487276 |
| 1449560_at | 2310043L02Rik | -3.1202 | 4.6487276 |
| 1457254_x_at | 6330442E10Rik | -3.1172 | 4.6487276 |
| 1458467_at | 1110032D16Rik | -3.1137 | 4.6487276 |
| 1426649_at | Tmeff1 | -3.1134 | 4.6487276 |
| 1451279_at | Erc1 | -3.1123 | 4.6487276 |
| 1456482_at | Pik3r3 | -3.1092 | 4.6487276 |
| 1417576_a_at | Otub2 | -3.1088 | 4.6487276 |
| 1428283_at | Cyp2s1 | -3.1088 | 4.6487276 |
| 1415862_at | Tyrp1 | -3.1068 | 4.6487276 |
| 1458096_at | Nhs | -3.1054 | 4.6487276 |
| 1453297_at | 4933417O08Rik | -3.102 | 4.6487276 |
| 1449471_at | Kcnmb4 | -3.0996 | 4.6487276 |
| 1438877_at | Trpm6 | -3.0985 | 4.6487276 |
| 1442089_at | --- | -3.0949 | 4.6487276 |
| 1426910_at | --- | -3.0912 | 4.6487276 |
| 1450985_a_at | Tjp2 | -3.091 | 4.6487276 |
| 1441307_at | A030010K20Rik | -3.0901 | 4.6487276 |
| 1437840_s_at | LOC673662 | -3.0881 | 4.6487276 |
| 1437620_x_at | Aldh18a1 | -3.0864 | 4.6487276 |
| 1451774_at | LOC435285 | -3.0856 | 4.6487276 |
| 1452138_a_at | Ace2 | -3.0851 | 4.6487276 |
| 1456815_at | Foxn1 | -3.0844 | 4.6487276 |
| 1449873_at | Bmp8a | -3.0841 | 4.6487276 |
| 1428996_at | 4833426J09Rik | -3.0824 | 4.6487276 |
| 1439760_x_at | Upk1b | -3.0805 | 4.6487276 |
| 1420674_at | Pla2g2e | -3.0772 | 4.6487276 |
| 1419052_at | Ovol1 | -3.073 | 4.6487276 |
| 1422670_at | Rnd2 | -3.0695 | 4.6487276 |
| 1449625_at | --- | -3.0689 | 4.6487276 |
| 1450536_s_at | Krtap12-1 | -3.0676 | 4.6487276 |
| 1422155_at | Hist2h3c2 | -3.0661 | 4.6487276 |
| 1441055_at | Palm2 | -3.066 | 4.6487276 |
| 1453286_at | Plxna2 | -3.0628 | 4.6487276 |
| 1430703_at | --- | -3.0618 | 4.6487276 |
| 1451690_a_at | Pvrl4 | -3.0615 | 4.6487276 |
| 1426876_at | 4732466D17Rik | -3.0546 | 4.6487276 |
| 1429909_at | 4833411O04Rik | -3.053 | 4.6487276 |
| 1449683_x_at | Tubb2b | -3.0521 | 4.6487276 |
| 1456350_at | Zfp451 | -3.0511 | 4.6487276 |
| 1442341_at | Kalrn | -3.0492 | 4.6487276 |
| 1454539_at | 4933417G07Rik | -3.048 | 4.6487276 |
| 1458680_at | --- | -3.0467 | 4.6487276 |
| 1460149_at | Atp13a5 | -3.0444 | 4.6487276 |
| 1421340_at | Map3k5 /// LOC675366 | -3.0418 | 4.6487276 |
| 1432473_a_at | Abca14 | -3.0415 | 4.6487276 |
| 1459702_at | --- | -3.0396 | 4.6487276 |
| 1420640_at | Jmy | -3.0394 | 4.6487276 |
| 1418211_at | p | -3.0391 | 4.6487276 |
| 1455464_x_at | --- | -3.0375 | 4.6487276 |
| 1416662_at | Sardh | -3.0375 | 4.6487276 |
| 1421156_a_at | Dsc2 | -3.0375 | 4.6487276 |
| 1416274_at | Ctns | -3.0358 | 4.6487276 |
| 1417717_a_at | Tyr | -3.0327 | 4.6487276 |
| 1453614_a_at | Nfe2l3 | -3.0295 | 4.6487276 |
| 1417585_at | Nup210 | -3.0278 | 4.6487276 |
| 1441693_at | Adamts3 | -3.0221 | 4.6487276 |
| 1452834_at | 2600010E01Rik | -3.0215 | 4.6487276 |
| 1451551_at | Krt84 | -3.0103 | 4.6487276 |
| 1450645_at | Mt4 | -3.0102 | 4.6487276 |
| 1420944_at | Zfp185 | -3.0086 | 4.6487276 |
| 1433735_a_at | Tmem64 | -3.0074 | 4.6487276 |
| 1417101_at | Hspa2 | -3.0019 | 4.6487276 |
| 1458386_at | C030009J22Rik | -3.0016 | 4.6487276 |
| 1420943_at | Zfp185 | -2.9969 | 4.6487276 |
| 1458057_at | --- | -2.9959 | 4.6487276 |
| 1419707_at | Krtap14 | -2.9908 | 4.6487276 |
| 1451382_at | Chac1 | -2.9899 | 4.6487276 |
| 1418207_at | Fxyd4 | -2.9896 | 4.6487276 |
| 1431650_at | 1110054P19Rik | -2.9895 | 4.6487276 |
| 1421118_a_at | Gpr56 | -2.9868 | 4.6487276 |
| 1450131_a_at | Bspry | -2.9859 | 4.6487276 |
| 1447046_at | Dnajb13 | -2.9837 | 4.6487276 |
| 1425693_at | Braf | -2.983 | 4.6487276 |
| 1423376_a_at | Dok4 | -2.9826 | 4.6487276 |
| 1416997_a_at | Hap1 | -2.9787 | 4.6487276 |
| 1421494_at | 1110033F04Rik | -2.9709 | 4.6487276 |
| 1435452_at | Tmem20 | -2.967 | 4.6487276 |
| 1425103_at | Ace2 | -2.9655 | 4.6487276 |
| 1452469_a_at | Smtn | -2.9651 | 4.6487276 |
| 1419555_at | Elf5 | -2.9632 | 4.6487276 |
| 1442470_at | --- | -2.9627 | 4.6487276 |
| 1418205_at | --- | -2.9624 | 4.6487276 |
| 1442434_at | D8Ertd82e | -2.9606 | 4.6487276 |
| 1434070_at | Jag1 | -2.9592 | 4.6487276 |
| 1448562_at | Upp1 | -2.9575 | 4.6487276 |
| 1433743_at | Dach1 | -2.9541 | 4.6487276 |
| 1460236_at | Klk10 | -2.9482 | 4.6487276 |
| 1449776_at | --- | -2.9482 | 4.6487276 |
| 1442999_at | B930036G03Rik | -2.9453 | 4.6487276 |
| 1415977_at | Isyna1 | -2.9445 | 4.6487276 |
| 1447807_s_at | Plekhh1 | -2.9435 | 4.6487276 |
| 1429032_at | Micalcl | -2.9416 | 4.6487276 |
| 1426663_s_at | Slc45a3 | -2.9409 | 4.6487276 |
| 1421634_at | Capn8 | -2.9408 | 4.6487276 |
| 1450089_a_at | Srprb | -2.9397 | 4.6487276 |
| 1418176_at | Vdr | -2.9376 | 4.6487276 |
| 1434599_a_at | Tjp2 | -2.9359 | 4.6487276 |
| 1448501_at | Tspan6 | -2.9331 | 4.6487276 |
| 1439521_at | --- | -2.9327 | 4.6487276 |
| 1437899_at | Gm776 | -2.9319 | 4.6487276 |
| 1436590_at | Ppp1r3b | -2.9312 | 4.6487276 |
| 1453283_at | Pgm1 | -2.9309 | 4.6487276 |
| 1427366_at | Krtap3-1 | -2.9304 | 4.6487276 |
| 1454742_at | Rasgef1b | -2.9284 | 4.6487276 |
| 1439634_at | 4930505D03Rik | -2.9206 | 4.6487276 |
| 1448502_at | Slc16a7 | -2.9189 | 4.6487276 |
| 1440534_at | --- | -2.9185 | 4.6487276 |
| 1424616_s_at | Frag1 | -2.9178 | 4.6487276 |
| 1419556_at | Elf5 | -2.9164 | 4.6487276 |
| 1455959_s_at | --- | -2.9162 | 4.6487276 |
| 1424910_at | Kif12 | -2.9159 | 4.6487276 |
| 1420639_at | Jmy | -2.9154 | 4.6487276 |
| 1424674_at | Slc39a6 | -2.9151 | 4.6487276 |
| 1445200_at | Farp1 | -2.9143 | 4.6487276 |
| 1430509_at | --- | -2.9114 | 4.6487276 |
| 1434496_at | Plk3 | -2.9113 | 4.6487276 |
| 1449091_at | Cldn8 | -2.9081 | 4.6487276 |
| 1430448_at | 6720418B01Rik | -2.8988 | 4.6487276 |
| 1437431_at | --- | -2.8969 | 4.6487276 |
| 1429024_at | Rbm20 | -2.896 | 4.6487276 |
| 1450886_at | Gsg2 | -2.8947 | 4.6487276 |
| 1459850_x_at | Glrb | -2.8941 | 4.6487276 |
| 1417422_at | Gnmt | -2.8928 | 4.6487276 |
| 1441094_at | Adh6b | -2.8915 | 4.6487276 |
| 1451123_at | C330016O10Rik | -2.8893 | 4.6487276 |
| 1426664_x_at | Slc45a3 | -2.8887 | 4.6487276 |
| 1433571_at | Serinc5 | -2.8865 | 4.6487276 |
| 1457317_at | --- | -2.886 | 4.6487276 |
| 1419465_at | Nkd2 | -2.8849 | 4.6487276 |
| 1455239_at | 6330512M04Rik | -2.8842 | 4.6487276 |
| 1456172_at | Txndc5 | -2.884 | 4.6487276 |
| 1438354_x_at | Cnn3 | -2.88 | 4.6487276 |
| 1422979_at | Suv39h2 | -2.8774 | 4.6487276 |
| 1441927_at | Syt7 | -2.8767 | 4.6487276 |
| 1422958_at | Krtap5-4 | -2.8766 | 4.6487276 |
| 1453269_at | Unc5b | -2.8764 | 4.6487276 |
| 1447941_x_at | Braf | -2.8713 | 4.6487276 |
| 1437407_at | 9930039A11Rik | -2.8671 | 4.6487276 |
| 1418352_at | Hsd17b2 | -2.8666 | 4.6487276 |
| 1438861_at | Bnc2 | -2.8665 | 4.6487276 |
| 1450813_a_at | Tnni1 | -2.8636 | 4.6487276 |
| 1450984_at | Tjp2 | -2.8603 | 4.6487276 |
| 1443785_x_at | Pdlim7 | -2.86 | 4.6487276 |
| 1429331_at | 4632427E13Rik | -2.8588 | 4.6487276 |
| 1424296_at | Gclc | -2.8576 | 4.6487276 |
| 1456186_at | 4631405J19Rik | -2.8573 | 4.6487276 |
| 1429183_at | Pkp2 | -2.8569 | 4.6487276 |
| 1423277_at | Ptprk | -2.8552 | 4.6487276 |
| 1425237_at | Krtap16-8 | -2.8539 | 4.6487276 |
| 1419845_at | Dlx1as | -2.8489 | 4.6487276 |
| 1438707_at | Atp13a4 | -2.8485 | 4.6487276 |
| 1455298_at | Id4 | -2.8473 | 4.6487276 |
| 1442554_s_at | Kalrn | -2.8448 | 4.6487276 |
| 1418237_s_at | Col18a1 | -2.8446 | 4.6487276 |
| 1458409_at | C86595 | -2.8436 | 4.6487276 |
| 1441956_s_at | --- | -2.8412 | 4.6487276 |
| 1437887_at | E130306M17Rik | -2.8248 | 4.6487276 |
| 1441982_at | Taf7 | -2.8216 | 4.6487276 |
| 1431042_at | Paqr8 | -2.8202 | 4.6487276 |
| 1419051_at | Ovol1 | -2.8155 | 4.6487276 |
| 1448229_s_at | Ccnd2 | -2.8153 | 4.6487276 |
| 1426552_a_at | Bcl11a | -2.8144 | 4.6487276 |
| 1457637_at | Prickle1 | -2.8118 | 4.6487276 |
| 1427017_at | Satb2 | -2.8105 | 4.6487276 |
| 1422735_at | Foxq1 | -2.8077 | 4.6487276 |
| 1427549_s_at | Krtap16-10 /// Krtap16-3 /// Krtap16-6 | -2.8046 | 4.6487276 |
| 1447940_a_at | Braf | -2.8033 | 4.6487276 |
| 1436458_at | Sema6a | -2.8031 | 4.6487276 |
| 1427800_at | Krtap16-2 | -2.8012 | 4.6487276 |
| 1430669_at | Krtap4-7 | -2.8009 | 4.6487276 |
| 1417939_at | Rad51ap1 | -2.7998 | 4.6487276 |
| 1451922_at | Lman1l | -2.7996 | 4.6487276 |
| 1417520_at | Nfe2l3 | -2.7984 | 4.6487276 |
| 1429321_at | Rnf149 | -2.7949 | 4.6487276 |
| 1441338_at | 5930412G12Rik | -2.7907 | 4.6487276 |
| 1429307_s_at | Lzic | -2.7904 | 4.6487276 |
| 1428319_at | Pdlim7 | -2.7896 | 4.6487276 |
| 1450945_at | Prkca | -2.7892 | 4.6487276 |
| 1449287_at | Srms | -2.7877 | 4.6487276 |
| 1451186_at | Isg20l1 | -2.7873 | 4.6487276 |
| 1422209_s_at | Krtap9-1 /// LOC432600 /// LOC546515 /// LOC670533 | -2.783 | 4.6487276 |
| 1444468_at | Paqr8 | -2.7792 | 4.6487276 |
| 1433460_at | Ttc7b | -2.7745 | 4.6487276 |
| 1454254_s_at | 1600029D21Rik | -2.7743 | 4.6487276 |
| 1425655_at | Krtap16-1 | -2.7733 | 4.6487276 |
| 1426263_at | Igsf4c | -2.7732 | 4.6487276 |
| 1451899_a_at | Gtf2ird1 | -2.7709 | 4.6487276 |
| 1428615_at | P2ry5 | -2.7705 | 4.6487276 |
| 1455398_at | Lrrc8c | -2.7699 | 4.6487276 |
| 1426959_at | Bdh1 | -2.7674 | 4.6487276 |
| 1421355_at | Tgm3 | -2.7666 | 4.6487276 |
| 1419429_at | Cntfr | -2.7637 | 4.6487276 |
| 1434306_at | Rab3ip | -2.7635 | 4.6487276 |
| 1458641_at | Braf | -2.7629 | 4.6487276 |
| 1417803_at | 1110032A04Rik | -2.7578 | 4.6487276 |
| 1416305_at | Sh3bp4 | -2.7567 | 4.6487276 |
| 1416233_at | Eif3s2 | -2.7565 | 4.6487276 |
| 1437226_x_at | Marcksl1 | -2.7562 | 4.6487276 |
| 1418569_at | Fblim1 | -2.7558 | 4.6487276 |
| 1443230_at | 5330421F07Rik | -2.7512 | 4.9666995 |
| 1450506_a_at | Isg20l1 | -2.7506 | 4.9666995 |
| 1452789_at | Snn | -2.7501 | 4.9666995 |
| 1435363_at | Plekhg1 | -2.7477 | 4.9666995 |
| 1460042_at | Slc23a3 | -2.7464 | 4.9666995 |
| 1451550_at | Ephb3 | -2.7446 | 4.9666995 |
| 1429772_at | Plxna2 | -2.7424 | 4.9666995 |
| 1449621_s_at | Thsd1 | -2.7419 | 4.9666995 |
| 1441886_at | Ccdc79 | -2.739 | 4.9666995 |
| 1416122_at | Ccnd2 | -2.7379 | 4.9666995 |
| 1423265_at | Minpp1 | -2.7373 | 4.9666995 |
| 1446573_at | Edil3 | -2.7368 | 4.9666995 |
| 1427964_at | Cmtm8 | -2.7343 | 4.9666995 |
| 1439887_at | --- | -2.7303 | 4.9666995 |
| 1456047_at | Pla2g4b | -2.7298 | 4.9666995 |
| 1423845_at | Csdc2 | -2.7298 | 4.9666995 |
| 1426048_s_at | Hrc /// Tcfap2a | -2.7268 | 4.9666995 |
| 1417300_at | Smpdl3b | -2.7262 | 4.9666995 |
| 1437654_at | 3110001K24Rik | -2.7252 | 4.9666995 |
| 1416118_at | LOC630539 | -2.7226 | 4.9666995 |
| 1421359_at | Ret | -2.7221 | 4.9666995 |
| 1419814_s_at | S100a1 | -2.7203 | 4.9666995 |
| 1455172_at | AU020094 | -2.7179 | 4.9666995 |
| 1438558_x_at | Foxq1 | -2.7157 | 4.9666995 |
| 1420009_at | Wwc1 | -2.7157 | 4.9666995 |
| 1454992_at | Slc7a1 | -2.7145 | 4.9666995 |
| 1437199_at | Dusp5 | -2.7129 | 4.9666995 |
| 1435455_at | C79267 | -2.711 | 4.9666995 |
| 1435053_s_at | Plekhh1 | -2.7108 | 4.9666995 |
| 1440593_at | Bnc2 | -2.7102 | 4.9666995 |
| 1434275_at | --- | -2.7092 | 4.9666995 |
| 1419225_at | Cacna2d3 | -2.7087 | 4.9666995 |
| 1426887_at | Nudt11 | -2.7085 | 4.9666995 |
| 1460048_at | Zdhhc13 | -2.708 | 4.9666995 |
| 1424456_at | Pvrl2 | -2.7067 | 4.9666995 |
| 1423364_a_at | Fts | -2.7055 | 4.9666995 |

| **B. Up-regulated (TERT-repressed) genes with FDR<0.05** | | | |
| --- | --- | --- | --- |
| **Probe Set** | **Gene Symbol** | **Score(d)** | **FDR(%)** |
| 1431833_a_at | Hmgcs2 | 7.046 | 2.7305078 |
| 1437665_at | Il22ra2 | 6.2587 | 2.7305078 |
| 1423858_a_at | Hmgcs2 | 5.79624 | 3.9251049 |
| 1456295_at | B230114P17Rik | 5.67698 | 4.0193075 |
| 1417707_at | B230342M21Rik | 5.23575 | 4.0193075 |
| 1431248_at | 5031426D15Rik | 5.00323 | 4.0193075 |
| 1458263_at | Cugbp2 | 5.00084 | 4.0193075 |
| 1453817_at | Abca6 | 4.84017 | 4.0193075 |
| 1448231_at | Fkbp5 | 4.76482 | 4.0193075 |
| 1437429_at | 5930437A14Rik | 4.7603 | 4.0193075 |
| 1453310_at | Ppil6 | 4.72121 | 4.0193075 |
| 1457108_at | Il1r2 | 4.71379 | 4.0193075 |
| 1459030_at | --- | 4.59238 | 4.0193075 |
| 1450398_at | Kcnk5 | 4.5906 | 4.0193075 |
| 1460123_at | Gpr1 | 4.5279 | 4.0193075 |
| 1419532_at | Il1r2 | 4.51363 | 4.0193075 |
| 1459878_a_at | A430107O13Rik | 4.49651 | 4.0193075 |
| 1433836_a_at | 8430408G22Rik | 4.48632 | 4.0193075 |
| 1443406_at | Plscr4 | 4.47502 | 4.0193075 |
| 1421564_at | Serpina3c | 4.41466 | 4.0193075 |
| 1417590_at | Cyp27a1 | 4.361 | 4.0193075 |
| 1459737_s_at | Ttr | 4.26125 | 4.0193075 |
| 1421425_a_at | Dscr1l1 | 4.24796 | 4.0193075 |
| 1458573_at | --- | 4.22343 | 4.0193075 |
| 1422788_at | Slc43a3 | 4.20905 | 4.0193075 |
| 1455683_a_at | Tbc1d8 | 4.1709 | 4.0193075 |
| 1437584_at | Ccnd3 | 4.14055 | 4.0193075 |
| 1419618_at | Bbox1 | 4.13758 | 4.0193075 |
| 1439537_at | --- | 4.13272 | 4.0193075 |
| 1424701_at | Pcdh20 | 4.12807 | 4.0193075 |
| 1443138_at | Sult5a1 | 4.0531 | 4.0193075 |
| 1433837_at | 8430408G22Rik | 4.03331 | 4.0193075 |
| 1442063_at | 5930437A14Rik | 4.00999 | 4.0193075 |
| 1415994_at | Cyp2e1 | 3.98184 | 4.0193075 |
| 1434186_at | Gpr23 | 3.96368 | 4.0193075 |
| 1443137_at | --- | 3.941 | 4.0193075 |
| 1423396_at | Agt | 3.915 | 4.0193075 |
| 1417600_at | Slc15a2 | 3.88754 | 4.0193075 |
| 1422903_at | Ly86 | 3.84087 | 4.0193075 |
| 1427345_a_at | Sult1a1 | 3.7745 | 4.0193075 |
| 1418858_at | Aox3 | 3.77047 | 4.0193075 |
| 1439128_at | Zbtb20 | 3.76933 | 4.0193075 |
| 1429900_at | 5330406M23Rik | 3.75302 | 4.0193075 |
| 1418601_at | Aldh1a7 | 3.75286 | 4.0193075 |
| 1418157_at | Nr2f1 | 3.75172 | 4.0193075 |
| 1450317_at | Clec2g | 3.7516 | 4.0193075 |
| 1460251_at | Fas | 3.69564 | 4.0193075 |
| 1416072_at | Cd34 | 3.69278 | 4.0193075 |
| 1428242_at | Hmha1 | 3.67798 | 4.0193075 |
| 1416225_at | Adh1 | 3.6738 | 4.0193075 |
| 1451342_at | Spon1 | 3.6628 | 4.0193075 |
| 1458628_at | Limk2 | 3.65647 | 4.0193075 |
| 1426938_at | Nova1 /// LOC664883 | 3.65603 | 4.0193075 |
| 1456968_at | Actn2 | 3.63767 | 4.0193075 |
| 1420349_at | Ptgfr | 3.63655 | 4.0193075 |
| 1446670_at | Cugbp2 | 3.62279 | 4.0193075 |
| 1417933_at | Igfbp6 | 3.61462 | 4.0193075 |
| 1436592_at | --- | 3.6113 | 4.0193075 |
| 1457656_s_at | C230085N15Rik | 3.59083 | 4.0193075 |
| 1422428_at | Acsbg1 | 3.57691 | 4.0193075 |
| 1423436_at | Gsta3 | 3.57471 | 4.0193075 |
| 1434642_at | Hsd17b11 | 3.56828 | 4.0193075 |
| 1456956_at | Ikzf2 | 3.54781 | 4.0193075 |
| 1437065_at | Zbtb20 | 3.54437 | 4.0193075 |
| 1442074_at | Clec2g | 3.53669 | 4.0193075 |
| 1451563_at | Emr4 | 3.50713 | 4.0193075 |
| 1436212_at | Tmem71 | 3.50109 | 4.0193075 |
| 1420249_s_at | Ccl6 | 3.4906 | 4.0193075 |
| 1417851_at | Cxcl13 | 3.48606 | 4.0193075 |
| 1454967_at | --- | 3.48457 | 4.0193075 |
| 1450784_at | Reck | 3.48046 | 4.0193075 |
| 1449484_at | Stc2 | 3.47238 | 4.0193075 |
| 1435943_at | Dpep1 | 3.46318 | 4.0193075 |
| 1433626_at | Plscr4 | 3.44795 | 4.0193075 |
| 1419674_a_at | Dpep1 | 3.44145 | 4.0193075 |
| 1449528_at | Figf | 3.42925 | 4.0193075 |
| 1458205_at | --- | 3.42912 | 4.0193075 |
| 1422816_a_at | Mutyh | 3.40436 | 4.0193075 |
| 1437542_at | A730095J18Rik | 3.40386 | 4.0193075 |
| 1423062_at | Igfbp3 | 3.40111 | 4.0193075 |
| 1451047_at | Itm2a | 3.40012 | 4.0193075 |
| 1451204_at | Scara5 | 3.39942 | 4.0193075 |
| 1416383_a_at | Pcx | 3.39808 | 4.0193075 |
| 1432163_at | 4930567K12Rik | 3.39391 | 4.0193075 |
| 1438443_at | Zbtb20 | 3.39303 | 4.0193075 |
| 1436397_at | BC027057 | 3.39041 | 4.0193075 |
| 1440587_at | --- | 3.38081 | 4.0193075 |
| 1456508_at | A030001H23Rik | 3.38062 | 4.0193075 |
| 1457932_at | Hivep2 | 3.37888 | 4.0193075 |
| 1423608_at | Itm2a | 3.36217 | 4.0193075 |
| 1455697_at | LOC629059 | 3.35894 | 4.0193075 |
| 1454388_at | 2900046F13Rik | 3.35581 | 4.0193075 |
| 1420796_at | Ahrr | 3.34625 | 4.0193075 |
| 1452416_at | Il6ra | 3.34308 | 4.0193075 |
| 1439395_at | 9630025I21Rik | 3.34266 | 4.0193075 |
| 1435541_at | Btc | 3.34109 | 4.0193075 |
| 1441506_at | Dcn | 3.32989 | 4.0193075 |
| 1441072_at | Cugbp2 | 3.32838 | 4.0193075 |
| 1423437_at | Gsta3 | 3.32604 | 4.0193075 |
| 1439586_at | LOC548102 | 3.31959 | 4.0193075 |
| 1448471_a_at | Ctla2a | 3.3126 | 4.0193075 |
| 1450734_at | Lztr2 | 3.30988 | 4.0193075 |
| 1422134_at | Fosb | 3.30614 | 4.0193075 |
| 1426622_a_at | Qpct /// LOC639942 | 3.30401 | 4.0193075 |
| 1433466_at | AI467606 | 3.29927 | 4.0193075 |
| 1440879_at | Abca9 | 3.29861 | 4.0193075 |
| 1437556_at | Zfhx4 | 3.28672 | 4.0193075 |
| 1456130_at | LOC553091 | 3.28359 | 4.0193075 |
| 1425260_at | Alb1 | 3.27933 | 4.0193075 |
| 1429030_at | C1qtnf7 | 3.27564 | 4.0193075 |
| 1448859_at | Cxcl13 | 3.27287 | 4.0193075 |
| 1425107_a_at | Lifr | 3.27231 | 4.0193075 |
| 1458268_s_at | Igfbp3 | 3.27194 | 4.0193075 |
| 1420401_a_at | Ramp3 | 3.26907 | 4.0193075 |
| 1455418_at | --- | 3.26269 | 4.0193075 |
| 1448628_at | Scg3 | 3.24814 | 4.1095058 |
| 1453435_a_at | Fmo2 | 3.24707 | 4.1095058 |
| 1417150_at | Slc6a4 | 3.24254 | 4.1095058 |
| 1416811_s_at | Ctla2a /// Ctla2b | 3.23894 | 4.1095058 |
| 1428332_at | 1500004A08Rik | 3.23878 | 4.1095058 |
| 1420097_at | D13Ertd787e | 3.23117 | 4.1095058 |
| 1427410_at | Dleu2 | 3.23091 | 4.1095058 |
| 1418131_at | Samhd1 | 3.22997 | 4.1095058 |
| 1459391_at | Abca5 | 3.22877 | 4.1095058 |
| 1421946_at | Crp | 3.22576 | 4.1095058 |
| 1429808_at | 1110020C03Rik | 3.21843 | 4.1095058 |
| 1417676_a_at | Ptpro | 3.21831 | 4.1095058 |
| 1446693_at | --- | 3.2155 | 4.1095058 |
| 1451787_at | Cyp2b10 | 3.21422 | 4.1095058 |
| 1433694_at | Pde3b | 3.20987 | 4.1095058 |
| 1434414_at | Foxred2 | 3.20452 | 4.1095058 |
| 1451827_a_at | Nox4 | 3.20031 | 4.1095058 |
| 1421921_at | Serpina3m | 3.20004 | 4.1095058 |
| 1450702_at | Hfe | 3.19362 | 4.1095058 |
| 1451054_at | Orm1 | 3.17016 | 4.1095058 |
| 1456510_x_at | Ubie | 3.16738 | 4.1095058 |
| 1426915_at | Dapk1 | 3.16535 | 4.1095058 |
| 1422905_s_at | Fmo2 | 3.16521 | 4.1095058 |
| 1447948_at | A430107O13Rik | 3.15848 | 4.1095058 |
| 1435465_at | Kbtbd11 | 3.14422 | 4.1095058 |
| 1437956_at | BB220380 | 3.14294 | 4.1095058 |
| 1449182_at | Retn | 3.13112 | 4.1095058 |
| 1451577_at | Zbtb20 | 3.12798 | 4.1095058 |
| 1417639_at | Slc22a4 | 3.12368 | 4.1095058 |
| 1439278_at | Zbtb20 | 3.12191 | 4.1095058 |
| 1452183_a_at | Gtl2 | 3.11288 | 4.1095058 |
| 1440777_x_at | --- | 3.11155 | 4.1095058 |
| 1429402_at | Glt8d2 | 3.10972 | 4.1095058 |
| 1435389_at | Reps2 | 3.10066 | 4.5735338 |
| 1426278_at | Ifi27 | 3.09904 | 4.5735338 |
| 1417995_at | Ptpn22 | 3.09715 | 4.5735338 |
| 1437598_at | Zbtb20 | 3.09606 | 4.5735338 |
| 1429012_at | Arhgef6 | 3.0897 | 4.5735338 |
| 1447314_at | Tgfbr3 | 3.08877 | 4.5735338 |
| 1448269_a_at | Klhl13 | 3.08605 | 4.5735338 |
| 1451353_at | Tm6sf1 | 3.086 | 4.5735338 |
| 1425863_a_at | Ptpro | 3.08444 | 4.5735338 |
| 1425644_at | Lepr | 3.07135 | 4.5735338 |
| 1420877_at | 6-Sep | 3.06984 | 4.5735338 |
| 1417852_x_at | Clca1 | 3.06872 | 4.5735338 |
| 1445545_at | Tmem77 | 3.06254 | 4.5735338 |
| 1453924_a_at | Ptgfr | 3.05853 | 4.5735338 |
| 1443906_at | Cd55 | 3.05661 | 4.5735338 |
| 1436044_at | Scn7a | 3.05285 | 4.5735338 |
| 1422746_s_at | Bicd2 | 3.0322 | 4.5735338 |
| 1426758_s_at | Gtl2 | 3.02668 | 4.5735338 |
| 1451767_at | Ncf1 | 3.01884 | 4.5735338 |
| 1426231_at | Vit | 3.01018 | 4.5735338 |
| 1441779_at | 9530006C21Rik | 3.00791 | 4.5735338 |
| 1451361_a_at | Pnpla7 | 3.00592 | 4.5735338 |
| 1440728_at | Kcnma1 | 3.00525 | 4.5735338 |
| 1422257_s_at | Cyp2b10 | 3.00502 | 4.5735338 |
| 1451336_at | Lgals4 | 2.99965 | 4.5735338 |
| 1445518_at | Zfhx1b | 2.99815 | 4.5735338 |
| 1425645_s_at | Cyp2b10 | 2.99681 | 4.5735338 |
| 1451527_at | Pcolce2 | 2.99411 | 4.5735338 |
| 1439930_at | Smarca2 | 2.99406 | 4.5735338 |
| 1447790_at | Erdr1 | 2.9925 | 4.5735338 |
| 1451542_at | Ssbp2 | 2.98969 | 4.5735338 |
| 1425274_at | Asph | 2.98953 | 4.5735338 |
| 1436043_at | Scn7a | 2.98945 | 4.5735338 |
| 1422046_at | Itgam | 2.98617 | 4.5735338 |
| 1449816_at | Sult5a1 | 2.98592 | 4.5735338 |
| 1422188_s_at | Tcrg /// LOC670271 | 2.98279 | 4.5735338 |
| 1419144_at | Cd163 | 2.9821 | 4.5735338 |
| 1418752_at | Aldh3a1 | 2.97901 | 4.5735338 |
| 1440096_at | Ecm2 | 2.97261 | 4.5735338 |
| 1437070_at | Cdc14b | 2.9696 | 4.5735338 |
| 1417702_a_at | Hnmt | 2.96609 | 4.5735338 |
| 1422892_s_at | H2-Ea | 2.9632 | 4.5735338 |
| 1454984_at | AW061234 | 2.958 | 4.5735338 |
| 1422064_a_at | Zbtb20 | 2.95793 | 4.5735338 |
| 1443602_at | Ifngr1 | 2.95442 | 4.5735338 |
| 1448842_at | Cdo1 | 2.95407 | 4.5735338 |
| 1442389_at | Oas1f | 2.95289 | 4.5735338 |
| 1423238_at | Itgb1bp2 | 2.94552 | 4.5735338 |
| 1416760_at | Galntl1 | 2.94468 | 4.5735338 |
| 1424729_at | BC054059 | 2.94107 | 4.5735338 |
| 1443937_at | AI790276 | 2.93769 | 4.5735338 |
| 1422011_s_at | Xlr /// 3830403N18Rik | 2.93438 | 4.5735338 |
| 1449015_at | Retnla | 2.93346 | 4.5735338 |
| 1439143_at | A930018M24Rik | 2.93313 | 4.5735338 |
| 1428975_at | Susd3 | 2.9302 | 4.5735338 |
| 1419120_at | Lyl1 | 2.92933 | 4.5735338 |
| 1442715_at | --- | 2.92896 | 4.5735338 |
| 1425275_at | Asph | 2.92636 | 4.5735338 |
| 1452657_at | Ap1s2 | 2.92264 | 4.5735338 |
| 1428157_at | Gng2 | 2.91568 | 4.5735338 |
| 1452905_at | Gtl2 /// Lphn1 | 2.91183 | 4.5735338 |
| 1424050_s_at | Fgfr1 | 2.90993 | 4.5735338 |
| 1450639_at | Slc28a2 /// LOC381417 | 2.9093 | 4.5735338 |
| 1434151_at | Mettl7a | 2.90806 | 4.5735338 |
| 1418589_a_at | Mlf1 | 2.90417 | 4.5735338 |
| 1423298_at | Add3 | 2.90261 | 4.5735338 |
| 1418494_at | Ebf2 | 2.89998 | 4.5735338 |
| 1444045_at | --- | 2.89826 | 4.5735338 |
| 1459840_s_at | Ccdc28b | 2.89823 | 4.5735338 |
| 1428614_at | Ldhd | 2.89618 | 4.5735338 |
| 1452352_at | Ctla2b | 2.89595 | 4.5735338 |
| 1457058_at | Adamts2 | 2.89587 | 4.5735338 |
| 1420466_at | Spt2 | 2.89134 | 4.5735338 |
| 1447928_at | Car5b | 2.89118 | 4.5735338 |
| 1432606_at | 2610012C04Rik | 2.89098 | 4.5735338 |
| 1452260_at | Cidec | 2.88258 | 4.9666995 |
| 1425896_a_at | Fbn1 | 2.87739 | 4.9666995 |
| 1449982_at | Il11 | 2.87643 | 4.9666995 |
| 1455549_at | Sestd1 | 2.87543 | 4.9666995 |
| 1458469_at | Cblb | 2.87495 | 4.9666995 |
| 1438954_x_at | --- | 2.87374 | 4.9666995 |
| 1444088_at | Tcrb-V13 | 2.87296 | 4.9666995 |
| 1444772_at | Smyd3 | 2.87178 | 4.9666995 |
| 1439777_at | B230218O03 | 2.8708 | 4.9666995 |
| 1440292_at | D13Ertd666e | 2.86975 | 4.9666995 |
| 1429418_at | Cdc14b | 2.86691 | 4.9666995 |
| 1450063_at | Fmn2 | 2.86665 | 4.9666995 |
| 1438800_at | Nagk | 2.86482 | 4.9666995 |
| 1419161_a_at | Nox4 | 2.86264 | 4.9666995 |
| 1438953_at | --- | 2.86151 | 4.9666995 |
| 1426026_at | Prpf6 | 2.86094 | 4.9666995 |
| 1436201_x_at | Mbp | 2.8592 | 4.9666995 |
| 1421471_at | Npy1r | 2.85905 | 4.9666995 |
| 1437114_at | A930001N09Rik | 2.85696 | 4.9666995 |
| 1428909_at | A130040M12Rik | 2.85314 | 4.9666995 |
| 1426225_at | Rbp4 | 2.85134 | 4.9666995 |
| 1438519_at | Klhl24 | 2.85107 | 4.9666995 |
| 1454858_x_at | Mettl7a | 2.85097 | 4.9666995 |
| 1423895_a_at | Cugbp2 | 2.84873 | 4.9666995 |
| 1439449_at | Satb1 | 2.84724 | 4.9666995 |
| 1428156_at | Gng2 | 2.8461 | 4.9666995 |
| 1430928_at | 2900002J02Rik | 2.83455 | 4.9666995 |
| 1456895_at | Cd209b | 2.83376 | 4.9666995 |
| 1426155_a_at | Osr2 | 2.83322 | 4.9666995 |
| 1438109_at | Clca5 | 2.83205 | 4.9666995 |
| 1427213_at | Pfkfb1 | 2.82764 | 4.9666995 |
| 1421854_at | Fgl2 | 2.82649 | 4.9666995 |
| 1434510_at | Papss2 | 2.82438 | 4.9666995 |
| 1428895_at | Rftn2 | 2.82234 | 4.9666995 |
| 1448409_at | Lrmp | 2.82191 | 4.9666995 |
| 1454651_x_at | Mbp | 2.82191 | 4.9666995 |
| 1418398_a_at | Tspan32 | 2.8209 | 4.9666995 |
| 1423439_at | Pck1 | 2.82035 | 4.9666995 |
| 1439665_at | Gpr23 | 2.81931 | 4.9666995 |
| 1448782_at | Txndc11 | 2.81391 | 4.9666995 |
| 1449106_at | Gpx3 | 2.81306 | 4.9666995 |
| 1456156_at | Lepr | 2.81082 | 4.9666995 |
| 1426144_x_at | Trdn | 2.81049 | 4.9666995 |
| 1439218_at | Ust | 2.80825 | 4.9666995 |
| 1451535_at | Il31ra | 2.80563 | 4.9666995 |
| 1443248_at | Tmco3 | 2.80086 | 4.9666995 |
| 1425357_a_at | Grem1 | 2.79872 | 4.9666995 |
| 1423607_at | Lum | 2.79825 | 4.9666995 |
| 1443858_at | LOC552905 | 2.79646 | 4.9666995 |
| 1443471_at | Zbtb20 | 2.79623 | 4.9666995 |
| 1452946_a_at | Rftn2 | 2.79411 | 4.9666995 |
| 1450663_at | Thbs2 | 2.7938 | 4.9666995 |
| 1448649_at | Enpep | 2.79187 | 4.9666995 |
| 1447531_x_at | --- | 2.7911 | 4.9666995 |
| 1426574_a_at | Add3 | 2.79048 | 4.9666995 |
| 1458455_at | Abra | 2.78174 | 4.9666995 |
| 1435708_at | Gls | 2.7817 | 4.9666995 |
| 1453255_at | Slc43a1 | 2.78102 | 4.9666995 |
| 1425458_a_at | Grb10 | 2.78101 | 4.9666995 |
| 1426143_at | Trdn | 2.78028 | 4.9666995 |
| 1451707_s_at | Slc41a3 | 2.77338 | 4.9666995 |
| 1439380_x_at | Gtl2 | 2.77224 | 4.9666995 |
| 1417429_at | Fmo1 | 2.77185 | 4.9666995 |
| 1429459_at | Sema3d | 2.76985 | 4.9666995 |
| 1443340_at | Cbara1 | 2.76939 | 4.9666995 |
| 1430182_a_at | 1700006J14Rik | 2.76528 | 4.9666995 |
| 1425875_a_at | Lepr | 2.7649 | 4.9666995 |
| 1447903_x_at | Ap1s2 | 2.76277 | 4.9666995 |
| 1454425_at | 5830495A06Rik | 2.76259 | 4.9666995 |
| 1423297_at | Add3 | 2.76124 | 4.9666995 |
| 1437886_at | Klhl6 | 2.76053 | 4.9666995 |
| 1435396_at | Stxbp6 | 2.76009 | 4.9666995 |
| 1425120_x_at | 1810023F06Rik | 2.75999 | 4.9666995 |
| 1439348_at | S100a10 | 2.7597 | 4.9666995 |
| 1444712_at | Gm739 /// LOC672930 | 2.75748 | 4.9666995 |
| 1445781_at | Sh3d19 | 2.75519 | 4.9666995 |
| 1436054_at | 9130227C08Rik | 2.75418 | 4.9666995 |
| 1440935_at | Grb10 | 2.75312 | 4.9666995 |
| 1456720_at | 1500041B16Rik | 2.75037 | 4.9666995 |

| **C. Input parameters** | | | | | | | | | |
| --- | --- | --- | --- | --- | --- | --- | --- | --- | --- |
|  |  |  |  |  |  |  |  |  |  |
| **Data type?** | | | | | | | | Two class unpaired timecourse | |
| **Arrays centered?** | | | | | | | | FALSE |  |
| **Delta** | | | | | | | | 0.93456515 |  |
| **Minimum fold change** | | | | | | | | 0 |  |
| **Test statistic** | | | | | | | | standard |  |
| **Time summary method** | | | | | | | | signed.area |  |
| **Are data are log scale?** | | | | | | | | FALSE |  |
| **Number of permutations** | | | | | | | | 10000 |  |
| **Input percentile for exchangeability factor s0** | | | | | | | | Automatic choice | |
| **Number of neighbors for KNN** | | | | | | | | 10 |  |
| **Seed for Random number generator** | | | | | | | | 12345678 |  |
