# Supplementary material for: TERT Promotes Epithelial Proliferation through Transcriptional Control of a Myc- and Wnt-Related Developmental Program
Source: PLoS Genet. 2008 Jan 18;4(1):e10. doi: 10.1371/journal.pgen.0040010 (PMC2211538; doi:10.1371/journal.pgen.0040010)
Supplement: Supplementary file 10 [file pgen.0040010.st004.doc]

**Table S4. List of chromosomal gene clusters among TERT-regulated genes**

| **Chr.** | **# genes**  **from the list** | **Cluster** | **Names of genes in the cluster**  **(<100kb apart between transcriptional start sites)** | **Local**  **p** | **Overall p** |
| --- | --- | --- | --- | --- | --- |
| 1 | 55 | 1 | Bag2 , Zfp451 | 0.2070 | <0.0001 |
|  |  | 2-4 | A030005L19Rik , A030014E15Rik , A030003K21Rik , LOC676250 |  |  |
|  |  | 5 | Fmo1 , Fmo2 |  |  |
| 2 | 45 | 6 | B230218O03 , Cugbp2 | 0.3969 |  |
|  |  | 7 | Slc43a1 , Slc43a3 |  |  |
|  |  | 8 | 4833411O04Rik , 2600010E01Rik |  |  |
| 3 | 28 | 9 | S100a1 , S100a3 | 0.1172 |  |
|  |  | 10 | Tchhl1 , S100a10 |  |  |
|  |  | 11 | Adh1 , Adh6b |  |  |
| 4 | 42 | 12 | Prpf4 , Bspry | 0.0008 |  |
|  |  | 13 | Tyrp1 , D4Bwg0951e |  |  |
|  |  | 14 | Ctps , Cited4 |  |  |
|  |  | 15 | Heyl , Bmp8a |  |  |
|  |  | 16-17 | Marcksl1 , Eif3s2 , Ccdc28b |  |  |
|  |  | 18-20 | Rcc2 , LOC670543 , Padi3 , Padi1 |  |  |
| 5 | 30 | 21 | Cldn4 , Cldn3 | 0.6983 |  |
| 7 | 44 | 22 | Igsf4c , 2310033E01Rik | 0.0108 |  |
|  |  | 23 | Cyp2s1 , Cyp2b10 |  |  |
|  |  | 24 | Lgals4 , Capn12 |  |  |
|  |  | 25-29 | Dusp8 , Krtap5-5 , Krtap5-2 , Krtap5-1 , Krtap5-4 , 6330512M04Rik |  |  |
| 8 | 26 | 30 | Sult5a1 , Dpep1 | 0.7277 |  |
| 10 | 39 | 31 | 5830408B19Rik , E130306M17Rik | 0.0683 |  |
|  |  | 32 | Ifngr1 , Il22ra2 |  |  |
|  |  | 33 | 4732454E20Rik , BB146404 |  |  |
|  |  | 34 | AI646023 , Ggt1 |  |  |
|  |  | 35 | Krtap12-1 , A030009A09Rik |  |  |
|  |  | 36 | Dcn , Lum |  |  |
| 11 | 56 | 37 | 1500004A08Rik , Limk2 | <0.0001 |  |
|  |  | 38 | Gsg2 , Ctns |  |  |
|  |  | 39 | Dlx3 , A730090H04Rik |  |  |
|  |  | 40 | Krt26 , Krt28 |  |  |
|  |  | 41-47 | Krtap3-1 , 2310043L02Rik , 2310040M23Rik , Krtap2-4 , A030010K20Rik , 1110033F04Rik , Krtap4-7 , 1110054P19Rik |  |  |
|  |  | 48-49 | LOC670482 , LOC435285 , LOC670533 |  |  |
|  |  | 50-55 | A030006P16Rik , Krt33a , Krt34 , Krt31 , Krt32 , Krt35 , Krt36 |  |  |
|  |  | 56-57 | Abca9 , Abca6 , Abca5 |  |  |
| 12 | 20 | 58 | Slc24a4 , C030009J22Rik | 0.0260 |  |
|  |  | 59-60 | Otub2 , Ifi27 , 1810023F06Rik |  |  |
| 13 | 33 | 61 | Susd3 , Bicd2 | 0.2228 |  |
|  |  | 62 | Ctla2b , Ctla2a |  |  |
|  |  | 63 | Cdc14b , D13Ertd666e |  |  |
| 14 | 26 | 64 | Kcnk5 , Kcnk16 | 0.1714 |  |
|  |  | 65 | C86595 , Slc7a8 |  |  |
|  |  | 66 | Gja3 , Gjb6 |  |  |
| 15 | 21 | 67 | Lifr , AW061234 | 0.0002 |  |
|  |  | 68 | Mettl7a , Ubie |  |  |
|  |  | 69-71 | Krt82 , Krt85 , 5430421N21Rik , Krt84 |  |  |
|  |  | 72 | Krt72 , Krt2 |  |  |
| 16 | 35 | 73 | Snn , Txndc11 | 0.0001 |  |
|  |  | 74 | Kalrn , Stxbp6 |  |  |
|  |  | 75-83 | Krtap13-1 , Krtap14 , Krtap15 , Krtap16-2 , Krtap16-1 , AY026312 , Krtap16-6 , 1110032D16Rik , Krtap6-1 , Krtap16-8 |  |  |
| 17 | 15 | 84-85 | Guca1b , AI661453 , Ccnd3 | 0.0188 |  |
|  |  | 86 | C230085N15Rik , Satb1 |  |  |
| X | 16 | 87 | Ap1s2 , Car5b | 0.3365 |  |

* p values are calculated by 10000 random iterations.

** Local p: p value by each chromosome.
