# Supplementary material for: TERT Promotes Epithelial Proliferation through Transcriptional Control of a Myc- and Wnt-Related Developmental Program
Source: PLoS Genet. 2008 Jan 18;4(1):e10. doi: 10.1371/journal.pgen.0040010 (PMC2211538; doi:10.1371/journal.pgen.0040010)
Supplement: Supplementary file 11 [file pgen.0040010.st005.doc]

**Table S5. Summary of c**hromosomal clustering scanning results

| Name | No total | No analyzed | Clustered? | P value |
| --- | --- | --- | --- | --- |
| TERT-regulated genes in the mouse skin | 626 | 586 | yes | <0.0001 |
| TERT-activated genes in the mouse skin | 388 | 366 | yes | <0.0001 |
| TERT-repressed genes in the mouse skin | 238 | 220 | yes | <0.0001 |

** p value from 10000 random permutations
