# Supplementary material for: TERT Promotes Epithelial Proliferation through Transcriptional Control of a Myc- and Wnt-Related Developmental Program
Source: PLoS Genet. 2008 Jan 18;4(1):e10. doi: 10.1371/journal.pgen.0040010 (PMC2211538; doi:10.1371/journal.pgen.0040010)
Supplement: Supplementary file 12 [file pgen.0040010.st006.doc]

**Table S6. Raw data for Hair-growth/anti-hair-growth comparison analysis**

A. Tables for Chi-square statistics

| **Expected** | **Unspecified** | **Growth** | **Anti-growth** | **Total** |
| --- | --- | --- | --- | --- |
| Up | 225.45 | 6.18 | 5.37 | 237 |
| Down | 369.09 | 10.12 | 8.79 | 388 |
| **Actual** | **Unspecified** | **Growth** | **Anti-growth** | **Total** |
| Up-regulated genes | 206 | 1 | 30 | 237 |
| Down-regulated genes | 287 | 100 | 1 | 388 |

| **Reference** | **number** | **% in genome** |
| --- | --- | --- |
| Growth | 887 | 2.608823529 |
| Anti-growth | 770 | 2.264705882 |
| Unspecified | 32343 | 95.12647059 |

B. Lists of genes

| **TERT-activated (388)** | **TERT-repressed (237)** | **Growth (887)** | **Anti-growth (770)** | **TERT-activated overlap  Growth (100)** | **TERT-activated overlap Anti-growth (1)** | **TERT-repressed overlap Growth (1)** | **TERT-repressed overlap Anti-growth (30)** |
| --- | --- | --- | --- | --- | --- | --- | --- |
| 0610040B09Rik | 1110020C03Rik | 0610007P14Rik | 0610007P06Rik | 1600029D21Rik | Ctse | Prpf6 | AW061234 |
| 1110032A04Rik | 1500004A08Rik | 0610016J10Rik | 0610010K06Rik | 5430421N21Rik |  |  | Aldh1a7 |
| 1110032D16Rik | 1500041B16Rik | 1100001H23Rik | 0610025P10Rik | 6330442E10Rik |  |  | Cd34 |
| 1110033F04Rik | 1700006J14Rik | 1110008F13Rik | 1100001G20Rik | Acp1 |  |  | Cd55 |
| 1110054P19Rik | 1810023F06Rik | 1110008P14Rik | 1110001A16Rik | Aldh1a3 |  |  | Cdo1 |
| 1600015H20Rik | 2610012C04Rik | 1110014J01Rik | 1110002B05Rik | Ass1 |  |  | Clca1 |
| 1600029D21Rik | 2900002J02Rik | 1110033J19Rik | 1190002H23Rik | Bag2 |  |  | Ctla2a |
| 1810012P15Rik | 2900046F13Rik | 1110049F12Rik | 1190017O12Rik | Bmp8a |  |  | Cyp2e1 |
| 2300006N05Rik | 4930567K12Rik | 1200011I18Rik | 1200015A19Rik | Bpgm |  |  | Dpep1 |
| 2310007B03Rik | 5031426D15Rik | 1200011O22Rik | 1810007M14Rik | Ccnd2 |  |  | Enpep |
| 2310033E01Rik | 5330406M23Rik | 1500001M20Rik | 1810015C04Rik | Ccrn4l |  |  | Fbn1 |
| 2310040M23Rik | 5830495A06Rik | 1500005K14Rik | 2310001A20Rik | Cdh6 |  |  | Fgfr1 |
| 2310043L02Rik | 5930437A14Rik | 1600029D21Rik | 2310061I04Rik | Cldn4 |  |  | Figf |
| 2510049J12Rik | 8430408G22Rik | 1700037H04Rik | 2410002F23Rik | Clu |  |  | Fkbp5 |
| 2600010E01Rik | 9130227C08Rik | 1810073G14Rik | 2510039O18Rik | Cmtm8 |  |  | Fmo1 |
| 3110001K24Rik | 9530006C21Rik | 2210409B22Rik | 2610005L07Rik | Cnn3 |  |  | Gtl2 |
| 3110032G18Rik | 9630025I21Rik | 2310008M10Rik | 2700060E02Rik | Col18a1 |  |  | Hivep2 |
| 4631405J19Rik | A030001H23Rik | 2310028O11Rik | 2810485I05Rik | Ctps |  |  | Hmha1 |
| 4632427E13Rik | A130040M12Rik | 2310040C09Rik | 2900062L11Rik | Ctse |  |  | Hsd17b11 |
| 4732454E20Rik | A430107O13Rik | 2310047M15Rik | 2900092E17Rik | Cutl1 |  |  | Igfbp3 |
| 4732466D17Rik | A730095J18Rik | 2310056P07Rik | 2900097C17Rik | Dct |  |  | Itm2a |
| 4732473B16Rik | A930001N09Rik | 2410015N17Rik | 3230401D17Rik | Dlx3 |  |  | Lum |
| 4833411O04Rik | A930018M24Rik | 2600005C20Rik | 3732412D22Rik | Dsc2 |  |  | Mlf1 |
| 4833426J09Rik | Abca5 | 2610002J02Rik | 4631408O11Rik | Dusp2 |  |  | Ptgfr |
| 4930438A08Rik | Abca6 | 2610019E17Rik | 4930432O21Rik | Elf5 |  |  | Reck |
| 4930505D03Rik | Abca9 | 2610042O14Rik | 4933434E20Rik | Epha4 |  |  | Slc6a4 |
| 4933417G07Rik | Abra | 2610524H06Rik | 5230400G24Rik | Ephb3 |  |  | Smarca2 |
| 4933417O08Rik | Acsbg1 | 2700007P21Rik | 5730403B10Rik | Ero1l |  |  | Ssbp2 |
| 5330421F07Rik | Actn2 | 2700094K13Rik | 5730420B22Rik | Fhl2 |  |  | Stc2 |
| 5430407P10Rik | Adamts2 | 2810026P18Rik | 5730469M10Rik | Fhod3 |  |  | Thbs2 |
| 5430421N21Rik | Add3 | 2810037C14Rik | 5730494N06Rik | Foxn1 |  |  |  |
| 5830408B19Rik | Adh1 | 2810417H13Rik | 6330409N04Rik | Frag1 |  |  |  |
| 5930412G12Rik | Agt | 3110001A13Rik | 9130229H14Rik | Gja1 |  |  |  |
| 6330442E10Rik | Ahrr | 3732413I11Rik | A030007L17Rik | Gjb6 |  |  |  |
| 6330512M04Rik | AI467606 | 4833408C14Rik | A030009H04Rik | Gnmt |  |  |  |
| 6720418B01Rik | AI790276 | 5430432M24Rik | Aatk | Gpr56 |  |  |  |
| 9930039A11Rik | Alb1 | 6330442E10Rik | Abcb1b | Hspa2 |  |  |  |
| A030001D16Rik | Aldh1a7 | 6330577E15Rik | Abcb7 | Id4 |  |  |  |
| A030003K21Rik | Aldh3a1 | AA409316 | Abcc9 | Krt31 |  |  |  |
| A030005K14Rik | Aox3 | AA536749 | Abcg2 | Krt32 |  |  |  |
| A030005L19Rik | Ap1s2 | AI413582 | Abl1 | Krt34 |  |  |  |
| A030006P16Rik | Arhgef6 | AL033314 | Ablim1 | Krt35 |  |  |  |
| A030009A09Rik | Asph | AU024582 | Acaa1b | Krt72 |  |  |  |
| A030010K20Rik | AW061234 | Abce1 | Acadl | Krt84 |  |  |  |
| A030014E15Rik | B230114P17Rik | Abhd5 | Acsl1 | Krt85 |  |  |  |
| A730090H04Rik | B230218O03 | Acad9 | Acsl4 | Krtap12-1 |  |  |  |
| Abca14 | B230342M21Rik | Acot7 | Actc1 | Krtap14 |  |  |  |
| Ace2 | BB220380 | Acot8 | Actn1 | Krtap3-1 |  |  |  |
| Acp1 | Bbox1 | Acp1 | Adh7 | Krtap5-1 |  |  |  |
| Adamts3 | BC027057 | Acpp | Adipoq | Krtap5-4 |  |  |  |
| Adh6b | BC054059 | Actn1 | Adrb3 | Krtap5-5 |  |  |  |
| AI646023 | Bicd2 | Agpat5 | Adss | Krtap6-1 |  |  |  |
| AI661453 | Btc | Ahcy | Agtrap | Krtap8-1 |  |  |  |
| Aldh18a1 | C1qtnf7 | Ahcyl1 | Ahr | Krtap9-1 |  |  |  |
| Aldh1a3 | C230085N15Rik | Ahk | Akap8 | LOC630539 |  |  |  |
| Ass1 | Car5b | Aim1 | Aldh1a7 | Lef1 |  |  |  |
| Atp13a4 | Cbara1 | Ak2 | Aldh9a1 | Ly6g6d |  |  |  |
| Atp13a5 | Cblb | Alas2 | Alk | Marcksl1 |  |  |  |
| Atp2c2 | Ccdc28b | Alcam | Alox12b | Minpp1 |  |  |  |
| AU020094 | Ccl6 | Aldh1a3 | Amot | Msx2 |  |  |  |
| AW011738 | Ccnd3 | Aldh3a2 | Angptl2 | Mt4 |  |  |  |
| AY026312 | Cd163 | Alox8 | Ank1 | Myef2 |  |  |  |
| B130021B11Rik | Cd209b | Apc1 | Ank3 | Nfe2l3 |  |  |  |
| B930036G03Rik | Cd34 | Ankrd13a | Ankrd1 | Padi1 |  |  |  |
| Bag2 | Cd55 | Ankrd49 | Antxr2 | Padi3 |  |  |  |
| Bambi | Cdc14b | Anp32b | Anxa11 | Padi4 |  |  |  |
| BB146404 | Cdo1 | Anp32e | Anxa3 | Pdlim7 |  |  |  |
| BC039632 | Cidec | Anpep | Anxa4 | Pgm1 |  |  |  |
| Bcl11a | Clca1 | Anxa1 | Anxa5 | Prkca |  |  |  |
| Bdh1 | Clca5 | Anxa2 | Anxa6 | Ptgds |  |  |  |
| Bmp8a | Clec2g | Ap2m1 | Aoc3 | Pvrl2 |  |  |  |
| Bnc2 | Crp | Apex1 | Ap1m1 | Rab3ip |  |  |  |
| Bpgm | Ctla2a | Aplp2 | Ap2a2 | Rcc2 |  |  |  |
| Braf | Ctla2b | Apoa1bp | Ap3b1 | Rnf149 |  |  |  |
| Bspry | Cugbp2 | Appbp1 | Ap3m1 | S100a3 |  |  |  |
| C030009J22Rik | Cxcl13 | Arf1 | Ap3s1 | Sct |  |  |  |
| C130092O11Rik | Cyp27a1 | Arf5 | Apbb1ip | Sfn |  |  |  |
| C230098O21Rik | Cyp2b10 | Arid2 | Apobec2 | Si |  |  |  |
| C330016O10Rik | Cyp2e1 | Arih2 | Arhgdia | Slain1 |  |  |  |
| C77651 | D13Ertd666e | Arl5a | Atp11a | Slc23a3 |  |  |  |
| C79267 | D13Ertd787e | Armet | Atp13a1 | Slc30a1 |  |  |  |
| C86595 | Dapk1 | Arpc1a | Atp1a2 | Slc39a6 |  |  |  |
| Cacna2d3 | Dcn | Arpc4 | Atp9a | Slc39a8 |  |  |  |
| Capn12 | Dleu2 | 4933428G09Rik | AW061234 | Slc4a1 |  |  |  |
| Capn8 | Dpep1 | 5430421N21Rik | AW112037 | Slc7a8 |  |  |  |
| Ccdc68 | Dscr1l1 | Ascc1 | Azi2 | Smad7 |  |  |  |
| Ccdc79 | Ebf2 | As1 | B3galnt1 | Srms |  |  |  |
| Ccnd2 | Ecm2 | Atp6v0a2 | Bag1 | Tgfa |  |  |  |
| Ccrn4l | Emr4 | Atp6v0d1 | Basp1 | Tgm3 |  |  |  |
| Cdh6 | Enpep | Atp6v1a | Bat5 | Tpm1 |  |  |  |
| Chac1 | Erdr1 | Asns | BC005624 | Tspan6 |  |  |  |
| Chn2 | Fas | Ass1 | BC008155 | Ttc7b |  |  |  |
| Cited4 | Fbn1 | Atox1 | BC010304 | Tubb3 |  |  |  |
| Cldn14 | Fgfr1 | Atp13a2 | BC018101 | Txndc5 |  |  |  |
| Cldn3 | Fgl2 | Atp13a3 | BC023829 | Tyrp1 |  |  |  |
| Cldn4 | Figf | Atp1a1 | BC032204 | Upp1 |  |  |  |
| Cldn8 | Fkbp5 | Atp5c1 | BC037034 | Vdr |  |  |  |
| Clic6 | Fmn2 | Atp5l | BC052328 | Wnt11 |  |  |  |
| Clu | Fmo1 | Atp6v1b2 | Bcap29 | Wnt5a |  |  |  |
| Cmah | Fmo2 | Atp7a | Bgn | Wwc1 |  |  |  |
| Cmtm8 | Fosb | Aurka | Bin1 |  |  |  |  |
| Cnn3 | Foxred2 | Aurkaip1 | Bmp1 |  |  |  |  |
| Cntfr | Galntl1 | Azin1 | Bri3 |  |  |  |  |
| Col18a1 | Gls | BC002199 | Brp44 |  |  |  |  |
| Cpm | Glt8d2 | BC003940 | Btbd14a |  |  |  |  |
| Cryba4 | Gm739 | BC011209 | C1qa |  |  |  |  |
| Csdc2 | Gng2 | BC018101 | C1qb |  |  |  |  |
| Ctns | Gpr1 | BC018242 | C1qc |  |  |  |  |
| Ctps | Gpr23 | BC023882 | C1qtnf3 |  |  |  |  |
| Ctse | Gpx3 | BC048355 | C77080 |  |  |  |  |
| Cutl1 | Grb10 | Bad | Cachd1 |  |  |  |  |
| Cybrd1 | Grem1 | Bag2 | Cacnb3 |  |  |  |  |
| Cyp11a1 | Gsta3 | Bak1 | Cacng1 |  |  |  |  |
| Cyp2s1 | Gtl2 | Bglap-rs1 | Cacybp |  |  |  |  |
| D12Ertd553e | H2-Ea | Birc5 | Calm4 |  |  |  |  |
| D4Bwg0951e | Hfe | Blmh | Calml4 |  |  |  |  |
| D630004K10Rik | Hivep2 | Bloc1s1 | Caml |  |  |  |  |
| D730001G18Rik | Hmgcs2 | Bmp2 | Capn3 |  |  |  |  |
| D8Ertd82e | Hmha1 | Bmp7 | Capn6 |  |  |  |  |
| Dach1 | Hnmt | Bmp8a | Car4 |  |  |  |  |
| Dcbld1 | Hsd17b11 | Bnc1 | Carhsp1 |  |  |  |  |
| Dcc | Ifi27 | Bnip3l | Casp14 |  |  |  |  |
| Dct | Ifngr1 | Bop1 | Casp8 |  |  |  |  |
| Dhcr24 | Igfbp3 | Bpgm | Cav1 |  |  |  |  |
| Dlx1as | Igfbp6 | Btg3 | Cav3 |  |  |  |  |
| Dlx3 | Ikzf2 | Bub1 | Cbx6 |  |  |  |  |
| Dnajb13 | Il11 | Bub3 | Ccdc90b |  |  |  |  |
| Dok4 | Il1r2 | Bzw2 | Ccl11 |  |  |  |  |
| Dsc2 | Il22ra2 | C1qdc2 | Ccl21b |  |  |  |  |
| Dusp2 | Il31ra | C5ar1 | Ccl9 |  |  |  |  |
| Dusp5 | Il6ra | Calm3 | Ccng1 |  |  |  |  |
| Dusp8 | Itgam | Capn5 | Ccnl2 |  |  |  |  |
| Dyrk3 | Itgb1bp2 | Capza1 | Ccr2 |  |  |  |  |
| E130306M17Rik | Itm2a | Car12 | Cd1d1 |  |  |  |  |
| Edg3 | Kbtbd11 | Car2 | Cd276 |  |  |  |  |
| Edil3 | Kcnk5 | Car6 | Cd34 |  |  |  |  |
| Eif3s2 | Kcnma1 | Ccar1 | Cd36 |  |  |  |  |
| Elf5 | Klhl13 | Ccdc95 | Cd47 |  |  |  |  |
| Epha4 | Klhl24 | Cckbr | Cd55 |  |  |  |  |
| Ephb1 | Klhl6 | Cc2 | Cd68 |  |  |  |  |
| Ephb3 | Ldhd | Ccnb2 | Cd81 |  |  |  |  |
| Eraf | Lepr | Ccnd1 | Cd93 |  |  |  |  |
| Erc1 | Lgals4 | Ccnd2 | Cdc42se1 |  |  |  |  |
| Ero1l | Lifr | Ccrn4l | Cdh13 |  |  |  |  |
| Expi | Limk2 | Cct3 | Cdh5 |  |  |  |  |
| Eya1 | LOC548102 | Cct5 | Cdkn1b |  |  |  |  |
| Fads3 | LOC552905 | Cd24a | Cdkn1c |  |  |  |  |
| Farp1 | LOC553091 | Cd44 | Cdo1 |  |  |  |  |
| Fblim1 | LOC629059 | Cdc20 | Cdr2l |  |  |  |  |
| Fgf22 | Lrmp | Cdc25c | Cebpd |  |  |  |  |
| Fgf5 | Lum | Cdc2a | Cfh |  |  |  |  |
| Fhl2 | Ly86 | Cdc6 | Cfl2 |  |  |  |  |
| Fhod3 | Lyl1 | Cdca8 | Cfp |  |  |  |  |
| Fjx1 | Lztr2 | Cdh3 | Chd1 |  |  |  |  |
| Foxn1 | Mbp | Cdh6 | Chpt1 |  |  |  |  |
| Foxq1 | Mettl7a | Cdk2 | Cirbp |  |  |  |  |
| Frag1 | Mlf1 | Cdk4 | Ckmt2 |  |  |  |  |
| Fts | Mutyh | Cdkn1a | Clca1 |  |  |  |  |
| Fxyd4 | Nagk | Cdsn | Cldn5 |  |  |  |  |
| Gabrp | Ncf1 | Cdt1 | Cln3 |  |  |  |  |
| Gclc | Nova1 | Cdv3 | Cma1 |  |  |  |  |
| Ggt1 | Nox4 | Cebpb | Cmtm6 |  |  |  |  |
| Gja1 | Npy1r | Cebpz | Cmya1 |  |  |  |  |
| Gja3 | Nr2f1 | Cfl1 | Cnot8 |  |  |  |  |
| Gjb6 | Oas1f | Chek2 | Col14a1 |  |  |  |  |
| Glrb | Orm1 | Cirh1a | Col17a1 |  |  |  |  |
| Gm266 | Osr2 | Ckap4 | Col3a1 |  |  |  |  |
| Gm776 | Papss2 | Cks1b | Col4a1 |  |  |  |  |
| Gng4 | Pcdh20 | Cks2 | Col5a1 |  |  |  |  |
| Gnmt | Pck1 | Cldn1 | Col5a2 |  |  |  |  |
| Gpr56 | Pcolce2 | Cldn4 | Col6a1 |  |  |  |  |
| Gsg2 | Pcx | Clic4 | Col6a2 |  |  |  |  |
| Gtf2ird1 | Pde3b | Clk3 | Col6a3 |  |  |  |  |
| Guca1b | Pfkfb1 | Clpp | Col7a1 |  |  |  |  |
| Hap1 | Plscr4 | Clu | Commd1 |  |  |  |  |
| Heyl | Pnpla7 | Cmpk | Copa |  |  |  |  |
| Hist2h3c2 | Ppil6 | Cmtm8 | Copb1 |  |  |  |  |
| Hrc , Tcfap2a | Prpf6 | Cnbp | Copg |  |  |  |  |
| Hsd17b2 | Ptgfr | Cnn3 | Coro2a |  |  |  |  |
| Hspa2 | Ptpn22 | Cnot7 | Cotl1 |  |  |  |  |
| Id4 | Ptpro | Col15a1 | Cox7a1 |  |  |  |  |
| Igsf4c | Qpct | Col18a1 | Cox7b |  |  |  |  |
| Irx4 | Ramp3 | Col3a1 | Cox8b |  |  |  |  |
| Isg20l1 | Rbp4 | Col4a2 | Cpa3 |  |  |  |  |
| Isyna1 | Reck | Col8a1 | Cpe |  |  |  |  |
| Itgb6 | Reps2 | Commd4 | Cradd |  |  |  |  |
| Jag1 | Retn | Cope | Creb3l1 |  |  |  |  |
| Jmy | Retnla | Cops4 | Crip1 |  |  |  |  |
| Kalrn | Rftn2 | Cops8 | Crtap |  |  |  |  |
| Kcne1 | S100a10 | Coro1c | Cryab |  |  |  |  |
| Kcnh1 | Samhd1 | Cox5b | Csda |  |  |  |  |
| Kcnk16 | Satb1 | Cox7a2 | Csf1 |  |  |  |  |
| Kcnmb4 | Scara5 | Cox7c | Csf1r |  |  |  |  |
| Kctd14 | Scg3 | Cpeb2 | Csf2rb2 |  |  |  |  |
| Kif12 | Scn7a | Cps1 | Csnk1g2 |  |  |  |  |
| Kif26a | Sema3d | Cpt1a | Cspg2 |  |  |  |  |
| Kitl | Serpina3c | Crct1 | Csrp2 |  |  |  |  |
| Klk10 | Serpina3m | Crebl1 | Csrp3 |  |  |  |  |
| Krt2 | Sestd1 | Crisp1 | Ctbp2 |  |  |  |  |
| Krt26 | Sh3d19 | Crlf3 | Ctla2a |  |  |  |  |
| Krt28 | Slc15a2 | Cry1 | Ctse |  |  |  |  |
| Krt2-ps1 | Slc22a4 | Crym | Ctsk |  |  |  |  |
| Krt31 | Slc28a2 | Csnk1a1 | Ctss |  |  |  |  |
| Krt32 | Slc41a3 | Csnk1d | Cxcr7 |  |  |  |  |
| Krt33a | Slc43a1 | Csrp1 | Cyb5 |  |  |  |  |
| Krt34 | Slc43a3 | Cstb | Cyb5r1 |  |  |  |  |
| Krt35 | Slc6a4 | Cstf3 | Cyba |  |  |  |  |
| Krt36 | Smarca2 | Ctbp2 | Cycs |  |  |  |  |
| Krt72 | Smyd3 | Ctcf | Cyp2e1 |  |  |  |  |
| Krt84 | Spon1 | Ctnnb1 | Cyp3a13 |  |  |  |  |
| Krt85 | Spt2 | Ctps | Cyp4b1 |  |  |  |  |
| Krtap12-1 | Ssbp2 | Ctsb | D8Ertd354e |  |  |  |  |
| Krtap13-1 | Stc2 | Ctsc | D9Ertd306e |  |  |  |  |
| Krtap14 | Stxbp6 | Ctsd | Dag1 |  |  |  |  |
| Krtap15 | Sult1a1 | Ctse | Dbp |  |  |  |  |
| Krtap16-10 | Sult5a1 | Ctsl | Ddah2 |  |  |  |  |
| Krtap16-2 | Susd3 | Cul1 | Ddx17 |  |  |  |  |
| Krtap16-8 | Tbc1d8 | Cutl1 | Ddx5 |  |  |  |  |
| Krtap2-4 | Tcrb-V13 | Cxcl14 | Deb1 |  |  |  |  |
| Krtap3-1 | Tcrg | Cxxc5 | Diap2 |  |  |  |  |
| Krtap4-7 | Tgfbr3 | Cyfip2 | Dkk2 |  |  |  |  |
| Krtap5-1 | Thbs2 | Cyhr1 | Dkk3 |  |  |  |  |
| Krtap5-2 | Tm6sf1 | Cyp17a1 | Dld |  |  |  |  |
| Krtap5-4 | Tmco3 | Cyp1b1 | Dlk1 |  |  |  |  |
| Krtap5-5 | Tmem71 | D030022P06Rik | Dja2 |  |  |  |  |
| Krtap6-1 | Tmem77 | D0H4S114 | Djc1 |  |  |  |  |
| Krtap8-1 | Trdn | D0HXS9928E | Djc19 |  |  |  |  |
| Krtap9-1 | Tspan32 | D11Ertd497e | Djc9 |  |  |  |  |
| Lap3 | Ttr | D15Mgi27 | Dnm1 |  |  |  |  |
| Lef1 | Txndc11 | D2Wsu81e | Dnm1l |  |  |  |  |
| Lman1l | Ubie | D5Ertd593e | Dock2 |  |  |  |  |
| LOC245128 | Ust | Dag1 | Dok1 |  |  |  |  |
| LOC435285 | Vit | Dbi | Dpep1 |  |  |  |  |
| LOC435727 | Xlr | Dck | Dpm1 |  |  |  |  |
| LOC630539 | Zbtb20 | Dct | Dpt |  |  |  |  |
| LOC665225 | Zfhx1b | Dctn2 | Dusp1 |  |  |  |  |
| LOC673662 | Zfhx4 | Dctn3 | Dusp12 |  |  |  |  |
| Lpin3 |  | Ddx1 | Dynlrb1 |  |  |  |  |
| Lrat |  | Ddx18 | Eed |  |  |  |  |
| Lrrc15 |  | Ddx3x | Ehhadh |  |  |  |  |
| Lrrc8c |  | Dek | Eid1 |  |  |  |  |
| Ly6g6d |  | Derl1 | Eif2ak2 |  |  |  |  |
| Lypd6 |  | Dhrs1 | Eif3s6 |  |  |  |  |
| Lzic |  | Dhrsx | Eif4ebp1 |  |  |  |  |
| Map3k5 |  | Dlx2 | Eln |  |  |  |  |
| Marcksl1 |  | Dlx3 | Emcn |  |  |  |  |
| Mfsd2 |  | Dmpk | Eh |  |  |  |  |
| Micalcl |  | Djb6 | Enpep |  |  |  |  |
| Minpp1 |  | Djc2 | Enpp2 |  |  |  |  |
| Msi1 |  | Dse1l2 | Eprs |  |  |  |  |
| Msx2 |  | Dnm2 | Eral1 |  |  |  |  |
| Mt4 |  | Dnmt1 | Etfa |  |  |  |  |
| Mybl2 |  | Dohh | Ets1 |  |  |  |  |
| Myef2 |  | Drg1 | Evl |  |  |  |  |
| Nanos1 |  | Dsc1 | Ext1 |  |  |  |  |
| Ndufs4 |  | Dsc2 | F2r |  |  |  |  |
| Nfe2l3 |  | Dscr3 | Fah |  |  |  |  |
| Nhs |  | Dsp | Fasn |  |  |  |  |
| Nkd2 |  | Dusp11 | Fbln1 |  |  |  |  |
| Nppc |  | Dusp19 | Fbn1 |  |  |  |  |
| Nudt11 |  | Dusp2 | Fcgr1 |  |  |  |  |
| Nup210 |  | Dusp6 | Fc |  |  |  |  |
| Otop2 |  | E230022H04Rik | Fgf13 |  |  |  |  |
| Otub2 |  | E4f1 | Fgfr1 |  |  |  |  |
| Ovol1 |  | EG238836 | Fhl1 |  |  |  |  |
| p |  | Edn1 | Figf |  |  |  |  |
| P2ry5 |  | Efhd1 | Fkbp10 |  |  |  |  |
| Padi1 |  | Efnb1 | Fkbp5 |  |  |  |  |
| Padi3 |  | Efnb3 | Flg |  |  |  |  |
| Padi4 |  | Eftud2 | Flt3 |  |  |  |  |
| Palm2 |  | Egfr | Fmo1 |  |  |  |  |
| Paqr8 |  | Egr2 | Fmr1 |  |  |  |  |
| Pard6b |  | Ehmt2 | Fnbp4 |  |  |  |  |
| Pdlim7 |  | Eif1a | Folr2 |  |  |  |  |
| Pfkfb2 |  | Eif1ay | Fos |  |  |  |  |
| Pgm1 |  | Eif2b1 | Fpgs |  |  |  |  |
| Phlda2 |  | Eif2b4 | Frrs1 |  |  |  |  |
| Pi15 |  | Eif2b5 | Fst |  |  |  |  |
| Pik3r3 |  | Eif2c2 | Fstl1 |  |  |  |  |
| Piwil2 |  | Eif2s1 | Fxr1h |  |  |  |  |
| Pkp2 |  | Eif3s10 | Fxyd5 |  |  |  |  |
| Pla2g2e |  | Eif3s4 | G3bp2 |  |  |  |  |
| Pla2g4b |  | Eif3s6ip | Gabpa |  |  |  |  |
| Plb1 |  | Eif3s7 | Gamt |  |  |  |  |
| Plekhg1 |  | Eif3s8 | Gab |  |  |  |  |
| Plekhh1 |  | Eif3s9 | Gas1 |  |  |  |  |
| Plk3 |  | Eif4a3 | Gas7 |  |  |  |  |
| Plxna2 |  | Eif4ebp2 | Gatm |  |  |  |  |
| Ppargc1a |  | Eif5 | Gbe1 |  |  |  |  |
| Ppp1r3b |  | Elf5 | Gbf1 |  |  |  |  |
| Prickle1 |  | Elof1 | Gdf8 |  |  |  |  |
| Prkca |  | Emid2 | Gem |  |  |  |  |
| Prpf4 |  | Emp1 | Ghitm |  |  |  |  |
| Psors1c2 |  | Emp2 | Ghr |  |  |  |  |
| Ptgds |  | Entpd4 | Gldc |  |  |  |  |
| Ptprk |  | Epha4 | Glg1 |  |  |  |  |
| Pvrl2 |  | Ephb3 | Glud1 |  |  |  |  |
| Pvrl4 |  | Eppb9 | Gi1 |  |  |  |  |
| Rab3ip |  | Ercc2 | Gi2 |  |  |  |  |
| Rad51ap1 |  | Erh | Gs |  |  |  |  |
| Rasgef1b |  | Ero1l | Gng12 |  |  |  |  |
| Rbm20 |  | Erp29 | Gnpat |  |  |  |  |
| Rcc2 |  | Esf1 | Gnpda1 |  |  |  |  |
| Ret |  | Etf1 | Golga4 |  |  |  |  |
| Rnd2 |  | Etv4 | Gpc3 |  |  |  |  |
| Rnf149 |  | Exod1 | Gpi1 |  |  |  |  |
| Rnf182 |  | Exosc4 | Gpld1 |  |  |  |  |
| S100a1 |  | Exosc5 | Gpr133 |  |  |  |  |
| S100a3 |  | Exosc7 | Grb2 |  |  |  |  |
| Sardh |  | Ezh2 | Grhpr |  |  |  |  |
| Satb2 |  | F2rl1 | Grik5 |  |  |  |  |
| Sct |  | Fabp5 | Grn |  |  |  |  |
| Sema6a |  | Faf1 | Grsf1 |  |  |  |  |
| Serinc2 |  | Farsla | Gsto1 |  |  |  |  |
| Serinc5 |  | Fbl | Gtf2a2 |  |  |  |  |
| Serpina1a |  | Fbp1 | Gtl2 |  |  |  |  |
| Sfn |  | Fbxo38 | Gusb |  |  |  |  |
| Sh3bp4 |  | Fbxw2 | Gyg |  |  |  |  |
| Si |  | Fdft1 | H19 |  |  |  |  |
| Slain1 |  | Fen1 | H1fx |  |  |  |  |
| Slc15a2 |  | Fgfbp1 | H2-Ke6 |  |  |  |  |
| Slc16a7 |  | Fhl2 | Hal |  |  |  |  |
| Slc23a3 |  | Fhod3 | Hbld1 |  |  |  |  |
| Slc24a4 |  | Fignl1 | Hck |  |  |  |  |
| Slc25a37 |  | Fkbp11 | Hdac2 |  |  |  |  |
| Slc30a1 |  | Fkbp8 | Herpud1 |  |  |  |  |
| Slc39a10 |  | Flnb | Hiatl1 |  |  |  |  |
| Slc39a6 |  | Fmn1 | Hivep2 |  |  |  |  |
| Slc39a8 |  | Foxn1 | Hmha1 |  |  |  |  |
| Slc40a1 |  | Foxp1 | Hnrpa3 |  |  |  |  |
| Slc45a3 |  | Frag1 | Hnrpdl |  |  |  |  |
| Slc4a1 |  | Fscn1 | Hnrpu |  |  |  |  |
| Slc5a5 |  | Fzd1 | Hoxa5 |  |  |  |  |
| Slc6a19 |  | Fzd2 | Hoxc6 |  |  |  |  |
| Slc7a1 |  | Fzd3 | Hsd17b11 |  |  |  |  |
| Slc7a8 |  | G6pd2 | Hsd3b7 |  |  |  |  |
| Slco4c1 |  | G6pdx | Hsf1 |  |  |  |  |
| Slco5a1 |  | Gadd45a | Hspe1 |  |  |  |  |
| Smad7 |  | Gars | Hspg2 |  |  |  |  |
| Smpdl3b |  | Gatad2a | Htra1 |  |  |  |  |
| Smtn |  | Gcat | Idh3g |  |  |  |  |
| Snn |  | Gclm | Ifi202b |  |  |  |  |
| Sorcs2 |  | Ghitm | Ifi203 |  |  |  |  |
| Srms |  | Gipc1 | Ifi204 |  |  |  |  |
| Srprb |  | Gja1 | Ifrd1 |  |  |  |  |
| St8sia6 |  | Gjb2 | Ift81 |  |  |  |  |
| Steap1 |  | Gjb3 | Igbp1 |  |  |  |  |
| Stox1 |  | Gjb4 | Igf1 |  |  |  |  |
| Stx6 |  | Gjb6 | Igf1r |  |  |  |  |
| Suv39h2 |  | Gli1 | Igfbp3 |  |  |  |  |
| Syt7 |  | Gnb2 | Igfbp4 |  |  |  |  |
| Sytl2 |  | Gnb2l1 | Igfbp5 |  |  |  |  |
| Taf7 |  | Gng5 | Igsf10 |  |  |  |  |
| Tagln3 |  | Gnmt | Iigp2 |  |  |  |  |
| Tchhl1 |  | Gosr2 | Ik |  |  |  |  |
| Tgfa |  | Gpc1 | Il13ra1 |  |  |  |  |
| Tgm3 |  | Gpr56 | Il13ra2 |  |  |  |  |
| Tgm6 |  | Gpx2 | Il17ra |  |  |  |  |
| Thsd1 |  | Gramd3 | Il6st |  |  |  |  |
| Tjp2 |  | Gspt1 | Ilf3 |  |  |  |  |
| Tm4sf1 |  | Gtf2b | Ilk |  |  |  |  |
| Tm4sf4 |  | Gtf2e2 | Ing1 |  |  |  |  |
| Tmeff1 |  | Gtf2h4 | Iqgap1 |  |  |  |  |
| Tmem20 |  | Gtf3a | Islr |  |  |  |  |
| Tmem64 |  | Gtpbp1 | Itga6 |  |  |  |  |
| Tmprss11e |  | Gtpbp4 | Itgb5 |  |  |  |  |
| Tmprss2 |  | H1f0 | Itm2a |  |  |  |  |
| Tnni1 |  | H2afv | Ivns1abp |  |  |  |  |
| Tpm1 |  | H2afx | Jak1 |  |  |  |  |
| Trpm6 |  | H2afy | Kcmf1 |  |  |  |  |
| Tspan6 |  | H2afz | Kctd12 |  |  |  |  |
| Ttc7b |  | Hars | Kdelr2 |  |  |  |  |
| Tubb2b |  | Hbb-b1 | Kdelr3 |  |  |  |  |
| Tubb3 |  | Hdac1 | Klf6 |  |  |  |  |
| Txndc5 |  | Hes6 | Klf9 |  |  |  |  |
| Tyrp1 |  | Hif1a | Klhdc2 |  |  |  |  |
| Unc5b |  | Hist3h2a | Klk8 |  |  |  |  |
| Upk1b |  | Hk1 | Kp1 |  |  |  |  |
| Upp1 |  | Hmgb1 | Kp6 |  |  |  |  |
| Vdr |  | Hmgb3 | Krt1 |  |  |  |  |
| Wdr73 |  | Hmgcr | Krtcap2 |  |  |  |  |
| Wnt11 |  | Hmox1 | Ktn1 |  |  |  |  |
| Wnt5a |  | Hprt1 | Lama4 |  |  |  |  |
| Wwc1 |  | Hps1 | Lamb2 |  |  |  |  |
| Zdhhc13 |  | Hras1 | Lamb3 |  |  |  |  |
| Zfp185 |  | Hsp90aa1 | Laptm5 |  |  |  |  |
| Zfp451 |  | Hspa2 | Lcp2 |  |  |  |  |
| Zfp618 |  | Hspa5 | Leprel2 |  |  |  |  |
|  |  | Ibrdc3 | Lgals1 |  |  |  |  |
|  |  | Id1 | Lgals9 |  |  |  |  |
|  |  | Id4 | Lgmn |  |  |  |  |
|  |  | Idh3a | Lig3 |  |  |  |  |
|  |  | Idi1 | Lmo2 |  |  |  |  |
|  |  | Hnrpa1 | LOC14433 |  |  |  |  |
|  |  | Hnrpa2b1 | LOC622469 |  |  |  |  |
|  |  | Hnrpl | LOC669888 |  |  |  |  |
|  |  | Hod | LOC672547 |  |  |  |  |
|  |  | Ier2 | Lox |  |  |  |  |
|  |  | Ier3 | Lphn1 |  |  |  |  |
|  |  | Igf2r | Lpin1 |  |  |  |  |
|  |  | Il1rn | Lpl |  |  |  |  |
|  |  | Ilvbl | Lrp1 |  |  |  |  |
|  |  | Ipo11 | Lsm14a |  |  |  |  |
|  |  | Iqgap3 | Lsp1 |  |  |  |  |
|  |  | Isy1 | Lta4h |  |  |  |  |
|  |  | Itgb4bp | Ltb4dh |  |  |  |  |
|  |  | Ivl | Ltbp2 |  |  |  |  |
|  |  | Jarid1b | Ltc4s |  |  |  |  |
|  |  | Jarid2 | Lum |  |  |  |  |
|  |  | Junb | Lxn |  |  |  |  |
|  |  | Jup | Ly6d |  |  |  |  |
|  |  | Kcnk2 | Lypla1 |  |  |  |  |
|  |  | Kif11 | M6pr |  |  |  |  |
|  |  | Kif22 | Mafg |  |  |  |  |
|  |  | Kif2c | Maged1 |  |  |  |  |
|  |  | Kif4 | Maged2 |  |  |  |  |
|  |  | Kit | Man1a |  |  |  |  |
|  |  | Klk6 | Map2k1 |  |  |  |  |
|  |  | Kp2 | Map3k3 |  |  |  |  |
|  |  | Krr1 | Mapkapk5 |  |  |  |  |
|  |  | Krt16 | Marveld1 |  |  |  |  |
|  |  | Krt17 | Mat2b |  |  |  |  |
|  |  | Krt25 | Matr3 |  |  |  |  |
|  |  | Krt27 | Mbc2 |  |  |  |  |
|  |  | Krt31 | Mbnl1 |  |  |  |  |
|  |  | Krt32 | Mcam |  |  |  |  |
|  |  | Krt33b | Mcl1 |  |  |  |  |
|  |  | Krt34 | Mcpt6 |  |  |  |  |
|  |  | Krt35 | Mdm2 |  |  |  |  |
|  |  | Krt6a | Mef2a |  |  |  |  |
|  |  | Krt71 | Mef2c |  |  |  |  |
|  |  | Krt72 | Mef2d |  |  |  |  |
|  |  | Krt81 | Meox2 |  |  |  |  |
|  |  | Krt84 | Mfap2 |  |  |  |  |
|  |  | Krt85 | Mfap4 |  |  |  |  |
|  |  | Krtap12-1 | Mfap5 |  |  |  |  |
|  |  | Krtap14 | Mgat1 |  |  |  |  |
|  |  | Kif20a | MGC107415 |  |  |  |  |
|  |  | Krtap3-1 | Mgea6 |  |  |  |  |
|  |  | Krtap3-3 | Mgll |  |  |  |  |
|  |  | Krtap5-1 | Mlf1 |  |  |  |  |
|  |  | Krtap5-4 | Mll1 |  |  |  |  |
|  |  | Krtap5-5 | Mmp12 |  |  |  |  |
|  |  | Krtap6-1 | Mpdz |  |  |  |  |
|  |  | Krtap6-2 | Mrpl44 |  |  |  |  |
|  |  | Krtap6-3 | Mrpl48 |  |  |  |  |
|  |  | Krtap8-1 | Msn |  |  |  |  |
|  |  | Krtap8-2 | Mtap4 |  |  |  |  |
|  |  | Krtap9-1 | Mtif2 |  |  |  |  |
|  |  | LOC630539 | Mtm1 |  |  |  |  |
|  |  | LOC668343 | Mybph |  |  |  |  |
|  |  | LOC669007 | Myd88 |  |  |  |  |
|  |  | LOC670044 | Myh9 |  |  |  |  |
|  |  | LOC671237 | Myl4 |  |  |  |  |
|  |  | LOC671904 | Mylk |  |  |  |  |
|  |  | LOC672274 | Mylpf |  |  |  |  |
|  |  | LOC675709 | Myom1 |  |  |  |  |
|  |  | LOC675857 | Myom2 |  |  |  |  |
|  |  | Lama5 | b1 |  |  |  |  |
|  |  | Lamb1-1 | ga |  |  |  |  |
|  |  | Lasp1 | gk |  |  |  |  |
|  |  | Lbr | ns |  |  |  |  |
|  |  | Lef1 | rs |  |  |  |  |
|  |  | Lgals7 | Nbn |  |  |  |  |
|  |  | Lias | Ncoa4 |  |  |  |  |
|  |  | Lin9 | Ncor2 |  |  |  |  |
|  |  | Llgl1 | Ndn |  |  |  |  |
|  |  | Llgl2 | Ndrg2 |  |  |  |  |
|  |  | Lmo4 | Ndufab1 |  |  |  |  |
|  |  | Lmo7 | Ndufs1 |  |  |  |  |
|  |  | Lnx1 | Nedd1 |  |  |  |  |
|  |  | Lor | Nedd4 |  |  |  |  |
|  |  | Lrrc59 | Nfatc1 |  |  |  |  |
|  |  | Lsmd1 | Nfatc2ip |  |  |  |  |
|  |  | Ltv1 | Nfic |  |  |  |  |
|  |  | Ly6g6d | Nfkbia |  |  |  |  |
|  |  | Lypla2 | Nfya |  |  |  |  |
|  |  | Mad2l1 | Nkiras1 |  |  |  |  |
|  |  | Mad2l1bp | Nnt |  |  |  |  |
|  |  | Maea | Nov |  |  |  |  |
|  |  | Mafb | Npnt |  |  |  |  |
|  |  | Map3k6 | Nptn |  |  |  |  |
|  |  | Map4k1 | Nr1d1 |  |  |  |  |
|  |  | Mapk6 | Nr1d2 |  |  |  |  |
|  |  | Marcks | Nrp1 |  |  |  |  |
|  |  | Marcksl1 | Nucb1 |  |  |  |  |
|  |  | Mc1r | Nudt4 |  |  |  |  |
|  |  | Mcm4 | Nxf1 |  |  |  |  |
|  |  | Mcm5 | Oaz2 |  |  |  |  |
|  |  | Mcm7 | Ogn |  |  |  |  |
|  |  | Mettl1 | Ogt |  |  |  |  |
|  |  | Mfge8 | Osbpl1a |  |  |  |  |
|  |  | Midn | Osmr |  |  |  |  |
|  |  | Minpp1 | P2rx4 |  |  |  |  |
|  |  | Mki67 | Pabpc4 |  |  |  |  |
|  |  | Mkrn1 | Paip2 |  |  |  |  |
|  |  | Mrpl18 | Parg |  |  |  |  |
|  |  | Mrpl20 | Pcbp1 |  |  |  |  |
|  |  | Mrpl37 | Pcf11 |  |  |  |  |
|  |  | Mrpl39 | Pcgf2 |  |  |  |  |
|  |  | Mrpl40 | Pcm1 |  |  |  |  |
|  |  | Mrpl50 | Pcmt1 |  |  |  |  |
|  |  | Mrps10 | Pcsk5 |  |  |  |  |
|  |  | Mrps18b | Pdcd6ip |  |  |  |  |
|  |  | Mrps33 | Pdgfra |  |  |  |  |
|  |  | Msx1 | Pdha1 |  |  |  |  |
|  |  | Msx2 | Pdlim3 |  |  |  |  |
|  |  | Mt2 | Pdpk1 |  |  |  |  |
|  |  | Mt4 | Pdxk |  |  |  |  |
|  |  | Mtm1 | Peg10 |  |  |  |  |
|  |  | Mvd | Peg3 |  |  |  |  |
|  |  | Mxd1 | Penk1 |  |  |  |  |
|  |  | Myb | Pepd |  |  |  |  |
|  |  | Mycl1 | Per2 |  |  |  |  |
|  |  | Mycn | Pgcp |  |  |  |  |
|  |  | Myd116 | Pgk1 |  |  |  |  |
|  |  | Myef2 | Pgrmc1 |  |  |  |  |
|  |  | Myeov2 | Pgrmc2 |  |  |  |  |
|  |  | Myh14 | Phka1 |  |  |  |  |
|  |  | Myh9 | Phldb2 |  |  |  |  |
|  |  | Myo10 | Phr1 |  |  |  |  |
|  |  | Myo1b | Phtf2 |  |  |  |  |
|  |  | Myo5b | Pias1 |  |  |  |  |
|  |  | Myo6 | Pias3 |  |  |  |  |
|  |  | b2 | Pim3 |  |  |  |  |
|  |  | rg1 | Pira1 |  |  |  |  |
|  |  | Ncl | Pkd1 |  |  |  |  |
|  |  | Ndufa8 | Pkd2 |  |  |  |  |
|  |  | Ndufb2 | Pkia |  |  |  |  |
|  |  | Nek2 | Pkm2 |  |  |  |  |
|  |  | Nek6 | Pla2r1 |  |  |  |  |
|  |  | Nfe2l3 | Plac8 |  |  |  |  |
|  |  | Nfil3 | Plac9 |  |  |  |  |
|  |  | Nid2 | Pld1 |  |  |  |  |
|  |  | Nmd3 | Pld4 |  |  |  |  |
|  |  | Nme2 | Plekhc1 |  |  |  |  |
|  |  | Nme4 | Plod3 |  |  |  |  |
|  |  | Nnp1 | Pls3 |  |  |  |  |
|  |  | Nol1 | Pnpt1 |  |  |  |  |
|  |  | Nol5 | Popdc3 |  |  |  |  |
|  |  | Nol5a | Postn |  |  |  |  |
|  |  | Nola1 | Pou2f1 |  |  |  |  |
|  |  | Nola2 | Pou2f3 |  |  |  |  |
|  |  | Nola3 | Ppap2b |  |  |  |  |
|  |  | Nono | Ppid |  |  |  |  |
|  |  | Notch1 | Ppp3ca |  |  |  |  |
|  |  | Npm1 | Ppp3cb |  |  |  |  |
|  |  | Npm3 | Ppp4r2 |  |  |  |  |
|  |  | Npy | Prei4 |  |  |  |  |
|  |  | Nr2f6 | Prepl |  |  |  |  |
|  |  | Nras | Prkcd |  |  |  |  |
|  |  | Nsdhl | Prlr |  |  |  |  |
|  |  | Nsun2 | Prosc |  |  |  |  |
|  |  | Nt5dc2 | Prpf38b |  |  |  |  |
|  |  | Nudt1 | Prps1 |  |  |  |  |
|  |  | Nudt21 | Prr6 |  |  |  |  |
|  |  | Numb | Prss23 |  |  |  |  |
|  |  | Nup35 | Pscd3 |  |  |  |  |
|  |  | Nup85 | Psme2 |  |  |  |  |
|  |  | Nxt1 | Ptcd3 |  |  |  |  |
|  |  | ORF61 | Ptch1 |  |  |  |  |
|  |  | Odz2 | Ptgfr |  |  |  |  |
|  |  | Orc6l | Ptgs1 |  |  |  |  |
|  |  | Otud5 | Pthr1 |  |  |  |  |
|  |  | Pa2g4 | Pts |  |  |  |  |
|  |  | Pabpc1 | Pttg1ip |  |  |  |  |
|  |  | Pabpn1 | Pygb |  |  |  |  |
|  |  | Padi1 | Qk |  |  |  |  |
|  |  | Padi3 | R3hdm1 |  |  |  |  |
|  |  | Pb1 | Rab10 |  |  |  |  |
|  |  | Pcnp | Rab11fip5 |  |  |  |  |
|  |  | Pdap1 | Rab12 |  |  |  |  |
|  |  | Pdcd2l | Rab14 |  |  |  |  |
|  |  | Pdia4 | Rab2 |  |  |  |  |
|  |  | Pdia6 | Rab28 |  |  |  |  |
|  |  | Pdk3 | Rab33b |  |  |  |  |
|  |  | Pdlim7 | Rab3d |  |  |  |  |
|  |  | Pdzd11 | Rab3il1 |  |  |  |  |
|  |  | Pebp1 | Rab5a |  |  |  |  |
|  |  | Pfdn5 | Rabgap1l |  |  |  |  |
|  |  | Pfn2 | Rad17 |  |  |  |  |
|  |  | Pgam1 | Rag1ap1 |  |  |  |  |
|  |  | Pgm1 | Ralbp1 |  |  |  |  |
|  |  | Pigq | Rap1gds1 |  |  |  |  |
|  |  | Pim1 | Rasa1 |  |  |  |  |
|  |  | Pip5k2c | Rasd1 |  |  |  |  |
|  |  | Pkp1 | Rassf2 |  |  |  |  |
|  |  | Pkp3 | Rbm39 |  |  |  |  |
|  |  | Pld3 | Rbms2 |  |  |  |  |
|  |  | Plk1 | Rbmx |  |  |  |  |
|  |  | Plk2 | Rbpms |  |  |  |  |
|  |  | Plp2 | Rdx |  |  |  |  |
|  |  | Plx2 | Reck |  |  |  |  |
|  |  | Pms2 | Reep1 |  |  |  |  |
|  |  | Pola2 | Rela |  |  |  |  |
|  |  | Pold1 | Rev3l |  |  |  |  |
|  |  | Pold3 | Rfk |  |  |  |  |
|  |  | Polr2l | Rfwd2 |  |  |  |  |
|  |  | Pp11r | Rhoq |  |  |  |  |
|  |  | Ppa2 | Rnf11 |  |  |  |  |
|  |  | Ppib | Rnf138 |  |  |  |  |
|  |  | Padi4 | RP23-336J1.4 |  |  |  |  |
|  |  | Ppif | Rpl24 |  |  |  |  |
|  |  | Ppil2 | Rpo1-2 |  |  |  |  |
|  |  | Ppl | Rpp14 |  |  |  |  |
|  |  | Ppm1b | Rprc1 |  |  |  |  |
|  |  | Ppp2r1b | Rras |  |  |  |  |
|  |  | Ppp4c | Rtn4 |  |  |  |  |
|  |  | Prc1 | S100a14 |  |  |  |  |
|  |  | Prdm1 | S100a4 |  |  |  |  |
|  |  | Prdx2 | Sar1b |  |  |  |  |
|  |  | Prkab1 | Sbf2 |  |  |  |  |
|  |  | Prkca | Scamp2 |  |  |  |  |
|  |  | Proc | Scp2 |  |  |  |  |
|  |  | Procr | Sdha |  |  |  |  |
|  |  | Prpf31 | Sec11a |  |  |  |  |
|  |  | Prpf6 | Sec23a |  |  |  |  |
|  |  | Psat1 | Sec24c |  |  |  |  |
|  |  | Psma1 | Selpl |  |  |  |  |
|  |  | Psma2 | Serf2 |  |  |  |  |
|  |  | Psmb3 | Serinc3 |  |  |  |  |
|  |  | Psmb4 | Serpi12 |  |  |  |  |
|  |  | Psmb7 | Serpinb6c |  |  |  |  |
|  |  | Psmc2 | Serpinf1 |  |  |  |  |
|  |  | Psmc3 | Serpinh1 |  |  |  |  |
|  |  | Psmc6 | Sf3b1 |  |  |  |  |
|  |  | Psmd12 | Sfrs11 |  |  |  |  |
|  |  | Psmd3 | Sft2d1 |  |  |  |  |
|  |  | Psmd8 | Sgcb |  |  |  |  |
|  |  | Ptch2 | Sgcg |  |  |  |  |
|  |  | Ptgds | Sgpp1 |  |  |  |  |
|  |  | Pthlh | Sh3bgr |  |  |  |  |
|  |  | Ptpn9 | Sh3glb1 |  |  |  |  |
|  |  | Ptprf | Slc12a2 |  |  |  |  |
|  |  | Pvrl2 | Slc1a5 |  |  |  |  |
|  |  | Pwp1 | Slc22a17 |  |  |  |  |
|  |  | Qars | Slc25a11 |  |  |  |  |
|  |  | Qscn6 | Slc25a36 |  |  |  |  |
|  |  | R3hcc1 | Slc38a4 |  |  |  |  |
|  |  | Rab3ip | Slc6a4 |  |  |  |  |
|  |  | Rab8a | Slc8a1 |  |  |  |  |
|  |  | Racgap1 | Slfn4 |  |  |  |  |
|  |  | Rala | Slk |  |  |  |  |
|  |  | Ralb | Slmap |  |  |  |  |
|  |  | Raly | Smarca2 |  |  |  |  |
|  |  | Ran | Smarca5 |  |  |  |  |
|  |  | Ranbp5 | Smoc2 |  |  |  |  |
|  |  | Rangap1 | Sms |  |  |  |  |
|  |  | Rars | Smyd1 |  |  |  |  |
|  |  | Rasl11b | Snx10 |  |  |  |  |
|  |  | Rbbp6 | Snx14 |  |  |  |  |
|  |  | Rbl1 | Socs2 |  |  |  |  |
|  |  | Rbms1 | Sod3 |  |  |  |  |
|  |  | Rbmx | Sorbs1 |  |  |  |  |
|  |  | Rbmxrt | Sos2 |  |  |  |  |
|  |  | Rbp1 | Sparcl1 |  |  |  |  |
|  |  | Rcc1 | Spg21 |  |  |  |  |
|  |  | Rcc2 | Spnb2 |  |  |  |  |
|  |  | Rdbp | Srgap2 |  |  |  |  |
|  |  | Reps1 | Ssbp2 |  |  |  |  |
|  |  | Rfc2 | Sspn |  |  |  |  |
|  |  | Rfc4 | St3gal1 |  |  |  |  |
|  |  | Rfk | St3gal6 |  |  |  |  |
|  |  | Rgl1 | St8sia4 |  |  |  |  |
|  |  | Rhoa | Stag2 |  |  |  |  |
|  |  | Rhod | Stat3 |  |  |  |  |
|  |  | Rhog | Stat5a |  |  |  |  |
|  |  | Rhpn2 | Statip1 |  |  |  |  |
|  |  | Rse1 | Stc2 |  |  |  |  |
|  |  | Rseh2a | Stk10 |  |  |  |  |
|  |  | Rseh2c | Strn3 |  |  |  |  |
|  |  | Rset2 | Stt3b |  |  |  |  |
|  |  | Rnf10 | Stxbp1 |  |  |  |  |
|  |  | Rnf103 | Stxbp3a |  |  |  |  |
|  |  | Rnf14 | Styx |  |  |  |  |
|  |  | Rnf149 | Suclg1 |  |  |  |  |
|  |  | Rnf4 | Supt4h2 |  |  |  |  |
|  |  | Rnh1 | Surf1 |  |  |  |  |
|  |  | Rpap1 | Svil |  |  |  |  |
|  |  | Rpl10a | Syncrip |  |  |  |  |
|  |  | Rpl13 | Sypl |  |  |  |  |
|  |  | Rpl13a | Sypl2 |  |  |  |  |
|  |  | Rpl14 | Tank |  |  |  |  |
|  |  | Rpl21 | Tbc1d14 |  |  |  |  |
|  |  | Rpl22l1 | Tbx15 |  |  |  |  |
|  |  | Rpl7a | Tceb1 |  |  |  |  |
|  |  | Rpl8 | Tcf12 |  |  |  |  |
|  |  | Rpo1-1 | Tcf3 |  |  |  |  |
|  |  | Rps16 | Tek |  |  |  |  |
|  |  | Rps27a | Tgfb3 |  |  |  |  |
|  |  | Rpsa | Tgm2 |  |  |  |  |
|  |  | Rrp9 | Thbd |  |  |  |  |
|  |  | Rsl1d1 | Thbs2 |  |  |  |  |
|  |  | Ruvbl1 | Thbs3 |  |  |  |  |
|  |  | Ruvbl2 | Thy1 |  |  |  |  |
|  |  | S100a3 | Timm17a |  |  |  |  |
|  |  | S100a6 | Tlk2 |  |  |  |  |
|  |  | S100a8 | Tm9sf3 |  |  |  |  |
|  |  | Sae1 | Tmeff2 |  |  |  |  |
|  |  | Sars | Tmem111 |  |  |  |  |
|  |  | Sars2 | Tmem126a |  |  |  |  |
|  |  | Scarb2 | Tmem150 |  |  |  |  |
|  |  | Scd2 | Tmem167 |  |  |  |  |
|  |  | Sct | Tmem176b |  |  |  |  |
|  |  | Sdhb | Tmem38b |  |  |  |  |
|  |  | Sec13 | Tmem43 |  |  |  |  |
|  |  | Sec61a1 | Tmem97 |  |  |  |  |
|  |  | Sec61b | Tnfaip8 |  |  |  |  |
|  |  | Sema4d | Tnfrsf1b |  |  |  |  |
|  |  | Senp3 | Tnfsf12 |  |  |  |  |
|  |  | Serpi1e | Tnks2 |  |  |  |  |
|  |  | Serpinb5 | Tnpo3 |  |  |  |  |
|  |  | Set | Tnxb |  |  |  |  |
|  |  | Sf3a1 | Tollip |  |  |  |  |
|  |  | Sf3a3 | Tomm70a |  |  |  |  |
|  |  | Sfn | Tpd52l2 |  |  |  |  |
|  |  | Sfpq | Trim21 |  |  |  |  |
|  |  | Sfrs2 | Trpc4ap |  |  |  |  |
|  |  | Sfrs9 | Tspan12 |  |  |  |  |
|  |  | Sgpl1 | Tspan9 |  |  |  |  |
|  |  | Sgta | Tspo |  |  |  |  |
|  |  | Sh3gl1 | Tufm |  |  |  |  |
|  |  | Shkbp1 | Tuft1 |  |  |  |  |
|  |  | Shmt1 | Tug1 |  |  |  |  |
|  |  | Shoc2 | Txndc4 |  |  |  |  |
|  |  | Si | Txnip |  |  |  |  |
|  |  | Sin3b | Tyrobp |  |  |  |  |
|  |  | Siva1 | Ubadc1 |  |  |  |  |
|  |  | Slain1 | Ubap2l |  |  |  |  |
|  |  | Slc10a6 | Ube1c |  |  |  |  |
|  |  | Slc23a3 | Ube2d3 |  |  |  |  |
|  |  | Slc25a15 | Ube2e1 |  |  |  |  |
|  |  | Slc25a5 | Ube2j2 |  |  |  |  |
|  |  | Slc30a1 | Ube2w |  |  |  |  |
|  |  | Slc31a1 | Ubl3 |  |  |  |  |
|  |  | Slc34a2 | Ubxd2 |  |  |  |  |
|  |  | Slc39a6 | Ugcg |  |  |  |  |
|  |  | Slc39a8 | Ugp2 |  |  |  |  |
|  |  | Slc3a2 | Unc84a |  |  |  |  |
|  |  | Slc41a1 | Uqcrb |  |  |  |  |
|  |  | Slc4a1 | Usp14 |  |  |  |  |
|  |  | Slc7a5 | Usp4 |  |  |  |  |
|  |  | Slc7a8 | Utrn |  |  |  |  |
|  |  | Sltm | Vamp2 |  |  |  |  |
|  |  | Smad2 | Vamp4 |  |  |  |  |
|  |  | Smad7 | Vcam1 |  |  |  |  |
|  |  | Smarca4 | Vdac3 |  |  |  |  |
|  |  | Smarcc1 | Vdp |  |  |  |  |
|  |  | Smc3 | Vps4b |  |  |  |  |
|  |  | Smc4 | Wdr13 |  |  |  |  |
|  |  | Smc6 | Wdr81 |  |  |  |  |
|  |  | Smn1 | Wdr82 |  |  |  |  |
|  |  | Smpd1 | Wnk1 |  |  |  |  |
|  |  | Snip1 | Wrnip1 |  |  |  |  |
|  |  | Snrpd3 | Xlkd1 |  |  |  |  |
|  |  | Sntb1 | Ywhaq |  |  |  |  |
|  |  | Snw1 | Zbtb16 |  |  |  |  |
|  |  | Snx12 | Zc3h11a |  |  |  |  |
|  |  | Snx3 | Zdhhc5 |  |  |  |  |
|  |  | Snx4 | Zfhx1a |  |  |  |  |
|  |  | Snx5 | Zfp106 |  |  |  |  |
|  |  | Snx9 | Zfp125 |  |  |  |  |
|  |  | Sox4 | Zfp275 |  |  |  |  |
|  |  | Spata13 | Zfp51 |  |  |  |  |
|  |  | Spint1 | Zfp644 |  |  |  |  |
|  |  | Sppl3 | Zfp650 |  |  |  |  |
|  |  | Sprr1a | Zfp706 |  |  |  |  |
|  |  | Sprr1b | Zic1 |  |  |  |  |
|  |  | Sprr2a | Znrd1 |  |  |  |  |
|  |  | Sprr2e | Zranb2 |  |  |  |  |
|  |  | Sprr2h |  |  |  |  |  |
|  |  | Sprr2i |  |  |  |  |  |
|  |  | Sptlc2 |  |  |  |  |  |
|  |  | Sqle |  |  |  |  |  |
|  |  | Srms |  |  |  |  |  |
|  |  | Srp54 |  |  |  |  |  |
|  |  | Srp68 |  |  |  |  |  |
|  |  | Ssr2 |  |  |  |  |  |
|  |  | Ssrp1 |  |  |  |  |  |
|  |  | Ssu72 |  |  |  |  |  |
|  |  | Stard10 |  |  |  |  |  |
|  |  | Stmn1 |  |  |  |  |  |
|  |  | Strbp |  |  |  |  |  |
|  |  | Strn |  |  |  |  |  |
|  |  | Stxbp2 |  |  |  |  |  |
|  |  | Sult2b1 |  |  |  |  |  |
|  |  | Sumo3 |  |  |  |  |  |
|  |  | Suz12 |  |  |  |  |  |
|  |  | Tacstd2 |  |  |  |  |  |
|  |  | Taf9 |  |  |  |  |  |
|  |  | Tagln2 |  |  |  |  |  |
|  |  | Taldo1 |  |  |  |  |  |
|  |  | Tax1bp3 |  |  |  |  |  |
|  |  | Tbc1d10a |  |  |  |  |  |
|  |  | Tcea1 |  |  |  |  |  |
|  |  | Tceb2 |  |  |  |  |  |
|  |  | Tceb3 |  |  |  |  |  |
|  |  | Tcf19 |  |  |  |  |  |
|  |  | Tcf7 |  |  |  |  |  |
|  |  | Tchh |  |  |  |  |  |
|  |  | Tdg |  |  |  |  |  |
|  |  | Tec |  |  |  |  |  |
|  |  | Tfrc |  |  |  |  |  |
|  |  | Tgfa |  |  |  |  |  |
|  |  | Tgif2 |  |  |  |  |  |
|  |  | Tgm3 |  |  |  |  |  |
|  |  | Thoc3 |  |  |  |  |  |
|  |  | Thrap3 |  |  |  |  |  |
|  |  | Thrap4 |  |  |  |  |  |
|  |  | Thumpd3 |  |  |  |  |  |
|  |  | Tia1 |  |  |  |  |  |
|  |  | Timm13 |  |  |  |  |  |
|  |  | Tipin |  |  |  |  |  |
|  |  | Tjp1 |  |  |  |  |  |
|  |  | Tk1 |  |  |  |  |  |
|  |  | Tle3 |  |  |  |  |  |
|  |  | Tmed2 |  |  |  |  |  |
|  |  | Tmem40 |  |  |  |  |  |
|  |  | Tmem41b |  |  |  |  |  |
|  |  | Tmem62 |  |  |  |  |  |
|  |  | Tmpo |  |  |  |  |  |
|  |  | Tmsb10 |  |  |  |  |  |
|  |  | Tnc |  |  |  |  |  |
|  |  | Tnk2 |  |  |  |  |  |
|  |  | Tom1 |  |  |  |  |  |
|  |  | Top1 |  |  |  |  |  |
|  |  | Top2a |  |  |  |  |  |
|  |  | Topbp1 |  |  |  |  |  |
|  |  | Tpd52l1 |  |  |  |  |  |
|  |  | Tpm1 |  |  |  |  |  |
|  |  | Traf4 |  |  |  |  |  |
|  |  | Trak2 |  |  |  |  |  |
|  |  | Trim27 |  |  |  |  |  |
|  |  | Trim28 |  |  |  |  |  |
|  |  | Trip12 |  |  |  |  |  |
|  |  | Trp53 |  |  |  |  |  |
|  |  | Trpm1 |  |  |  |  |  |
|  |  | Tspan6 |  |  |  |  |  |
|  |  | Tspan7 |  |  |  |  |  |
|  |  | Tssc4 |  |  |  |  |  |
|  |  | Ttc7b |  |  |  |  |  |
|  |  | Tuba1 |  |  |  |  |  |
|  |  | Tuba6 |  |  |  |  |  |
|  |  | Tubb2a |  |  |  |  |  |
|  |  | Tubb3 |  |  |  |  |  |
|  |  | Tubb5 |  |  |  |  |  |
|  |  | Tubb6 |  |  |  |  |  |
|  |  | Tulp3 |  |  |  |  |  |
|  |  | Txn1 |  |  |  |  |  |
|  |  | Txndc5 |  |  |  |  |  |
|  |  | Txnl2 |  |  |  |  |  |
|  |  | Txnrd1 |  |  |  |  |  |
|  |  | Tyr |  |  |  |  |  |
|  |  | Tyrp1 |  |  |  |  |  |
|  |  | Ube1x |  |  |  |  |  |
|  |  | Ube2o |  |  |  |  |  |
|  |  | Ube2z |  |  |  |  |  |
|  |  | Uck2 |  |  |  |  |  |
|  |  | Uhrf1 |  |  |  |  |  |
|  |  | Upp1 |  |  |  |  |  |
|  |  | Uqcrc1 |  |  |  |  |  |
|  |  | Uqcrq |  |  |  |  |  |
|  |  | Usp3 |  |  |  |  |  |
|  |  | Usp38 |  |  |  |  |  |
|  |  | Usp5 |  |  |  |  |  |
|  |  | Uxs1 |  |  |  |  |  |
|  |  | Vamp8 |  |  |  |  |  |
|  |  | Vdr |  |  |  |  |  |
|  |  | Vnn1 |  |  |  |  |  |
|  |  | Vps24 |  |  |  |  |  |
|  |  | Wdr26 |  |  |  |  |  |
|  |  | Wdr43 |  |  |  |  |  |
|  |  | Wdr45l |  |  |  |  |  |
|  |  | Wipi2 |  |  |  |  |  |
|  |  | Wiz |  |  |  |  |  |
|  |  | Wnt11 |  |  |  |  |  |
|  |  | Wnt5a |  |  |  |  |  |
|  |  | Wwc1 |  |  |  |  |  |
|  |  | Wwtr1 |  |  |  |  |  |
|  |  | Ykt6 |  |  |  |  |  |
|  |  | Ywhaz |  |  |  |  |  |
|  |  | ZBTB45 |  |  |  |  |  |
|  |  | Zc3h14 |  |  |  |  |  |
|  |  | Zc3h3 |  |  |  |  |  |
|  |  | Zdhhc6 |  |  |  |  |  |
|  |  | Zfand6 |  |  |  |  |  |
|  |  | Zfp692 |  |  |  |  |  |
