# Supplementary material for: TERT Promotes Epithelial Proliferation through Transcriptional Control of a Myc- and Wnt-Related Developmental Program
Source: PLoS Genet. 2008 Jan 18;4(1):e10. doi: 10.1371/journal.pgen.0040010 (PMC2211538; doi:10.1371/journal.pgen.0040010)
Supplement: Supplementary file 13 [file pgen.0040010.st007.doc]

**Table S7. Associations found by GSEA, between Wnt-/Myc-pathway/cell-cycling** gene sets and TERT gene signature, are statistically significant

| **Signaling pathway** | **Direction** | **#**  **Gene sets belong to**  **the pathway** | **#**  **Directionally matching**  **gene sets belong to the pathway (FDR<0.2) ^*^** | **%**  **Directionally matching**  **gene sets belong to**  **the pathway** | **#**  **All**  **gene sets in analysis** | **#**  **All**  **directionally matching**  **gene sets in analysis**  **(FDR<0.2) ^*^** | **%**  **Random chance**  **to see directionally matching**  **gene sets** | **p value^†^** | **Significant?** |
| --- | --- | --- | --- | --- | --- | --- | --- | --- | --- |
| **Myc** | Up | 10 | 6 | 60.0 % | 1134 | 88^‡^ | 7.8 % | 0.00004 | yes |
|  | Down | 4 | 4 | 100.0 % |  | 348^§^ | 30.7 % | 0.00905 | yes |
| **Wnt** | Up | 8 | 4 | 50.0 % | 1134 | 88^‡^ | 7.8 % | 0.00215 | yes |
|  | Down | 3 | 3 | 100.0 % |  | 348^§^ | 30.7 % | 0.02924 | yes |
| **Cell cycle** | Either | 11 | 10 | 90.9 % | 1134 | 436^¶^ | 38.4 % | 0.00054 | yes |
| **K-Ras** | Up | 1 | 1 | 100.0 % | 1134 | 88^‡^ | 7.8 % | 0.07841 | no |
|  | Down | 2 | 0 | 0 % |  | 348^§^ | 30.7 % | 0.99999 | no |
| **EGF** | Either | 3 | 0 | 0 % | 1134 | 436^¶^ | 38.4 % | 1.00000 | no |
| **TGFbeta** | Either | 5 | 0 | 0 % | 1134 | 436^¶^ | 38.4 % | 1.00000 | no |
| **MAPK** | Either | 6 | 0 | 0 % | 1134 | 436^¶^ | 38.4 % | 1.00000 | no |

* Number of directionally matching gene sets that are significantly associated with TERT signature.
- Up: FDR<0.2 with TERT-activated
- Down: FDR<0.2 with TERT-repressed
- Both: FDR<0.2 with either TERT-activated or TERT-repressed

† p values are calculated by one-tailed Fisher’s exact test

‡ All gene sets in analysis that are associated with TERT-activated by FDR<0.2.

: *Control for random association with TERT-activated.*

§ All gene sets in analysis that are associated with TERT-repressed by FDR<0.2.

: *Control for random association with TERT-repressed.*

¶ All gene sets in analysis that are associated with either TERT-activated or TERT-repressed by FDR<0.2.

: *Control for random association with either TERT-activated or TERT-repressed..*
