# Supplementary material for: TERT Promotes Epithelial Proliferation through Transcriptional Control of a Myc- and Wnt-Related Developmental Program
Source: PLoS Genet. 2008 Jan 18;4(1):e10. doi: 10.1371/journal.pgen.0040010 (PMC2211538; doi:10.1371/journal.pgen.0040010)
Supplement: Supplementary file 14 [file pgen.0040010.st008.doc]

**Table S8. TERT gene expression signature is enriched with evolutionarily conserved TCF/LEF, Myc (E-box), E2F and CRE-BP motif containing gene sets.**

A. Representative gene sets with strong correlation to TERT-activated genes

| **GENE SET NAME** | **SIZE** | **ES** | **NES** | **FDR**  **q-val** | **Conserved motif sequence in [-2kb,2kb] region around TSS** |
| --- | --- | --- | --- | --- | --- |
| **TCF/LEF motif containing gene sets** |  |  |  |  |  |
| V$TCF4_Q5 | 132 | -0.28 | -1.29 | 0.331 | SCTTTGAW |
| V$LEF1_Q6 | 154 | -0.27 | -1.29 | 0.365 | SWWCAAAGGG |
| **E2F motif containing gene sets** |  |  |  |  |  |
| V$E2F_01 | 46 | -0.46 | -1.79 | 0.083 | TWSGCGCGAAAAYKR |
| V$E2F1_Q4 | 155 | -0.25 | -1.24 | 0.358 | NTTSGCGG |
| **CRE-BP motif containing gene set** |  |  |  |  |  |
| V$CREBP1_Q2 | 139 | -0.26 | -1.22 | 0.352 | VGTGACGTMACN |

B. Representative gene sets with strong correlation to TERT-repressed genes

| **GENE SET NAME** | **SIZE** | **ES** | **NES** | **FDR**  **q-val** | **Conserved motif sequence in [-2kb,2kb] region around TSS** |
| --- | --- | --- | --- | --- | --- |
| **Myc (E-box) motif containing gene sets** | | | | | |
| V$NMYC_01 | 162 | 0.27 | 1.13 | 0.434 | NNCCACGTGNNN |
| V$MYCMAX_03 | 154 | 0.26 | 1.08 | 0.499 | NNNNNNNCACGTGNNNNNNN |
| **E2F motif containing gene set** |  |  |  |  |  |
| V$E2F1_Q3_01 | 1621 | 0.29 | 1.23 | 0.299 | TTGGCGCGRAANNGNM |
| **CRE-BP motif containing gene set** |  |  |  |  |  |
| V$CREBP1_01 | 111 | 0.31 | 1.23 | 0.302 | TTACGTAA |
