# Supplementary material for: TERT Promotes Epithelial Proliferation through Transcriptional Control of a Myc- and Wnt-Related Developmental Program
Source: PLoS Genet. 2008 Jan 18;4(1):e10. doi: 10.1371/journal.pgen.0040010 (PMC2211538; doi:10.1371/journal.pgen.0040010)
Supplement: Supplementary file 15 [file pgen.0040010.st009.doc]

**Table S9. Detailed results of *cis*-regulatory motif enrichment analysis**

A. [-2kb,+2kb] *cis*-regulatory motif scan *with* PhastCons conservation score threshold

| **[-2kb,2kb] with conservation threshold** | | **TERT- activated** | **Growth** | **Growth overlaps TERT- activated** | **Growth minus TERT- activated** | **TERT- activated minus Growth** | **Random 10000** |
| --- | --- | --- | --- | --- | --- | --- | --- |
| **Number genes analyzed** | | **326** | **842** | **96** | **728** | **228** | **10000** |
| **E2F/myc V$E2F1_Q4 TTTSGCGSG** | Genes | 1 | 22 | 0 | 22 | 0 | 83 |
|  | Prob. | 0.00306748 | 0.02612827 | 0 | 0.03021978 | 0 | 0.0083 |
|  | p value | 0.94929606 | 0.0006876 | 1 | 3.13E-04 | 1 |  |
| **E2F4/DP1 V$E2F4DP1_01 TTTSGCGC** | Genes | 2 | 23 | 0 | 23 | 2 | 135 |
|  | Prob. | 0.00613497 | 0.02731591 | 0 | 0.03159341 | 0.00877193 | 0.0135 |
|  | p value | 0.95005856 | 0.00799466 | 1 | 0.00299986 | 0.77415431 |  |
| **E2F V$E2F_Q6_01 NKCGCGCSAAAN** | Genes | 0 | 10 | 0 | 10 | 0 | 26 |
|  | Prob. | 0 | 0.01187648 | 0 | 0.01373626 | 0 | 0.0026 |
|  | p value | 0.99999984 | 0.00690837 | 0.99999984 | 0.00517797 | 0.99999984 |  |
| **TCF/LEF1 V$LEF1_Q6 SWWCAAAGGG** | Genes | 13 | 19 | 8 | 11 | 4 | 145 |
|  | Prob. | 0.0398773 | 0.02256532 | 0.08333333 | 0.01510989 | 0.01754386 | 0.0145 |
|  | p value | 0.0099676 | 0.06244803 | 0.00738484 | 0.44811993 | 0.36436255 |  |
| **V$MYCMAX_02 NANCACGTGNNW** | Genes | 8 | 23 | 3 | 20 | 5 | 165 |
|  | Prob. | 0.02453988 | 0.02731591 | 0.03125 | 0.02747253 | 0.02192982 | 0.0165 |
|  | p value | 0.17669127 | 0.03020816 | 0.20369927 | 0.03815901 | 0.28942818 |  |
| **V$MYCMAX_B GCCAYGYGSN** | Genes | 14 | 44 | 4 | 39 | 10 | 355 |
|  | Prob. | 0.04294479 | 0.05225653 | 0.04166667 | 0.05357143 | 0.04385965 | 0.0355 |
|  | p value | 0.25648757 | 0.01683841 | 0.3816579 | 0.01725324 | 0.27068561 |  |
| **[-2kb,2kb] with conservation threshold** | | **TERT- repressed** | **Anti- growth** | **Anti-growth overlaps TERT- repressed** | **Anti-growth minus TERT- repressed** | **TERT- repressed minus Anti-growth** | **Random 10000** |
| **Number genes analyzed** | | **199** | **727** | **27** | **682** | **172** | **10000** |
| **CRE-BP V$CREBP1_Q2 VGTGACGTMACN** | Genes | 1 | 8 | 0 | 8 | 1 | 41 |
|  | Prob. | 0.00502513 | 0.01100413 | 0 | 0.01173021 | 0.00581395 | 0.0041 |
|  | p value | 0.42736659 | 0.03915423 | 1 | 0.0337094 | 0.38442452 |  |
| **V$MYCMAX_02 NANCACGTGNNW** | Genes | 4 | 21 | 0 | 20 | 4 | 165 |
|  | Prob. | 0.0201005 | 0.02888583 | 0 | 0.02932551 | 0.02325581 | 0.0165 |
|  | p value | 0.35980693 | 0.02539199 | 1 | 0.02572473 | 0.27951023 |  |
| **V$MYC_Q2 CACGTGS** | Genes | 17 | 81 | 5 | 74 | 12 | 803 |
|  | Prob. | 0.08542714 | 0.11141678 | 0.18518519 | 0.1085044 | 0.06976744 | 0.0803 |
|  | p value | 0.39883705 | 0.00470229 | 0.0804438 | 0.01047478 | 0.70436303 |  |
| **V$NMYC_01 NNCCACGTGNNN** | Genes | 13 | 58 | 3 | 55 | 10 | 622 |
|  | Prob. | 0.06532663 | 0.07977992 | 0.11111111 | 0.08064516 | 0.05813953 | 0.0622 |
|  | p value | 0.42982368 | 0.04447411 | 0.20952945 | 0.04240515 | 0.58920925 |  |

* p values from one-tailed two proportion Z-statistics

** red: p<0.05

B. [-5kb,+1kb] *cis*-regulatory motif scan *with* PhastCons conservation score threshold

| **[-5kb,1kb]**  **with conservation threshold** | | **TERT- activated** | **Growth** | **Growth overlaps TERT- activated** | **Growth minus TERT- activated** | **TERT- activated minus Growth** | **Random 10000** |
| --- | --- | --- | --- | --- | --- | --- | --- |
| **Number genes analyzed** | | **326** | **842** | **96** | **728** | **228** | **10000** |
| **E2F/myc V$E2F1_Q4 TTTSGCGSG** | Genes | 1 | 22 | 0 | 22 | 0 | 87 |
|  | Prob. | 0.00306748 | 0.02612827 | 0 | 0.03021978 | 0 | 0.0087 |
|  | p value | 0.96078768 | 0.0008859 | 1 | 3.95E-04 | 1 |  |
| **E2F4/DP1 V$E2F4DP1_01 TTTSGCGC** | Genes | 2 | 24 | 0 | 24 | 2 | 132 |
|  | Prob. | 0.00613497 | 0.02850356 | 0 | 0.03296703 | 0.00877193 | 0.0132 |
|  | p value | 0.94289504 | 0.00443218 | 1 | 0.00162199 | 0.7596279 |  |
| **E2F V$E2F_Q6_01 NKCGCGCSAAAN** | Genes | 0 | 10 | 0 | 10 | 0 | 27 |
|  | Prob. | 0 | 0.01187648 | 0 | 0.01373626 | 0 | 0.0027 |
|  | p value | 0.9999999 | 0.00745413 | 0.9999999 | 0.00554228 | 0.9999999 |  |
| **TCF/LEF1 V$LEF1_Q6 SWWCAAAGGG** | Genes | 13 | 17 | 7 | 10 | 5 | 172 |
|  | Prob. | 0.0398773 | 0.02019002 | 0.07291667 | 0.01373626 | 0.02192982 | 0.0172 |
|  | p value | 0.01887117 | 0.27565272 | 0.01799062 | 0.7789868 | 0.31443118 |  |
| **V$MYCMAX_B GCCAYGYGSN** | Genes | 14 | 47 | 4 | 42 | 10 | 363 |
|  | Prob. | 0.04294479 | 0.05581948 | 0.04166667 | 0.05769231 | 0.04385965 | 0.0363 |
|  | p value | 0.27969607 | 0.00817483 | 0.39664499 | 0.00777081 | 0.29041088 |  |
| **V$MYC_Q2 CACGTGS** | Genes | 30 | 92 | 10 | 79 | 21 | 822 |
|  | Prob. | 0.09202454 | 0.10926366 | 0.10416667 | 0.10851648 | 0.09210526 | 0.0822 |
|  | p value | 0.27264625 | 0.00736528 | 0.24138704 | 0.01318421 | 0.30433289 |  |
| **V$NMYC_01 NNCCACGTGNNN** | Genes | 22 | 71 | 8 | 60 | 15 | 625 |
|  | Prob. | 0.06748466 | 0.08432304 | 0.08333333 | 0.08241758 | 0.06578947 | 0.0625 |
|  | p value | 0.36187771 | 0.01357288 | 0.23091262 | 0.02863023 | 0.42144069 |  |
| **[-5kb,1kb]**  **with conservation threshold** | | **TERT- repressed** | **Anti- growth** | **Anti-growth overlaps TERT- repressed** | **Anti-growth minus TERT- repressed** | **TERT- repressed minus Anti-growth** | **Random 10000** |
| **Number genes analyzed** | | **199** | **727** | **27** | **682** | **172** | **10000** |
| **CRE-BP V$CREBP1_Q2 VGTGACGTMACN** | Genes | 1 | 8 | 0 | 8 | 1 | 42 |
|  | Prob. | 0.00502513 | 0.01100413 | 0 | 0.01173021 | 0.00581395 | 0.0042 |
|  | p value | 0.43515651 | 0.04141235 | 1 | 0.03558517 | 0.39100752 |  |

* p values from one-tailed two proportion Z-statistics

** red: p<0.05

C. [-2kb,+2kb] *cis*-regulatory motif scan *without* conservation threshold

| **[-2kb,2kb]** | | **TERT- activated** | **Growth** | **Growth overlaps TERT- activated** | **Growth minus TERT- activated** | **TERT- activated minus Growth** | **Random 10000** |
| --- | --- | --- | --- | --- | --- | --- | --- |
| **Number genes analyzed** | | **326** | **842** | **96** | **728** | **228** | **10000** |
| **E2F/myc V$E2F1_Q4 TTTSGCGSG** | Genes | 9 | 45 | 3 | 42 | 5 | 316 |
|  | Prob. | 0.02760736 | 0.05344418 | 0.03125 | 0.05769231 | 0.02192982 | 0.0316 |
|  | p value | 0.6671396 | 2.99E-03 | 0.50782452 | 1.54E-03 | 0.83674765 |  |
| **E2F4/DP1 V$E2F4DP1_01 TTTSGCGC** | Genes | 16 | 59 | 4 | 55 | 12 | 497 |
|  | Prob. | 0.04907975 | 0.07007126 | 0.04166667 | 0.07554945 | 0.05263158 | 0.0497 |
|  | p value | 0.52033868 | 0.01228495 | 0.65235118 | 0.00499083 | 0.42225375 |  |
| **E2F V$E2F_Q6_01 NKCGCGCSAAAN** | Genes | 0 | 13 | 0 | 13 | 0 | 65 |
|  | Prob. | 0 | 0.01543943 | 0 | 0.01785714 | 0 | 0.0065 |
|  | p value | 1 | 0.01935433 | 1 | 0.01120103 | 1 |  |
| **TCF/LEF1 V$LEF1TCF1_Q4 MCTTTGWWSNY** | Genes | 100 | 234 | 35 | 193 | 65 | 2687 |
|  | Prob. | 0.30674847 | 0.27790974 | 0.36458333 | 0.26510989 | 0.28508772 | 0.2687 |
|  | p value | 0.0710803 | 0.28318939 | 0.02594942 | 0.58387558 | 0.29384502 |  |
| **V$MYCMAX_B GCCAYGYGSN** | Genes | 49 | 158 | 15 | 142 | 34 | 1479 |
|  | Prob. | 0.15030675 | 0.18764846 | 0.15625 | 0.19505495 | 0.14912281 | 0.1479 |
|  | p value | 0.4523659 | 0.00214239 | 0.41126349 | 0.00090107 | 0.47956017 |  |
| **[-2kb,2kb]** | | **TERT- repressed** | **Anti- growth** | **Anti-growth overlaps TERT- repressed** | **Anti-growth minus TERT- repressed** | **TERT- repressed minus Anti-growth** | **Random 10000** |
| **Number genes analyzed** | | **199** | **727** | **27** | **682** | **172** | **10000** |
| **V$MYCMAX_B GCCAYGYGSN** | Genes | 28 | 130 | 6 | 123 | 22 | 1479 |
|  | Prob. | 0.14070352 | 0.17881706 | 0.22222222 | 0.18035191 | 0.12790698 | 0.1479 |
|  | p value | 0.61370087 | 0.017405 | 0.17670156 | 0.01606368 | 0.78158631 |  |

* p values from one-tailed two proportion Z-statistics

** red: p<0.05, orange: 0.05<p<0.1

D. [-5kb,+1kb] *cis*-regulatory motif scan *without* conservation threshold

| **[-5kb,1kb]** | | **TERT- activated** | **Growth** | **Growth overlaps TERT- activated** | **Growth minus TERT- activated** | **TERT- activated minus Growth** | **Random 10000** |
| --- | --- | --- | --- | --- | --- | --- | --- |
| **Number genes analyzed** | | **326** | **842** | **96** | **728** | **228** | **10000** |
| **E2F/myc V$E2F1_Q4 TTTSGCGSG** | Genes | 9 | 44 | 3 | 41 | 5 | 331 |
|  | Prob. | 0.02760736 | 0.05225653 | 0.03125 | 0.05631868 | 0.02192982 | 0.0331 |
|  | p value | 0.72369384 | 7.50E-03 | 0.54127782 | 3.91E-03 | 0.87129995 |  |
| **E2F4/DP1 V$E2F4DP1_01 TTTSGCGC** | Genes | 19 | 66 | 5 | 61 | 14 | 538 |
|  | Prob. | 0.05828221 | 0.0783848 | 0.05208333 | 0.08379121 | 0.06140351 | 0.0538 |
|  | p value | 0.36680288 | 0.00495737 | 0.53002256 | 0.00216882 | 0.31792986 |  |
| **E2F V$E2F_Q6_01 NKCGCGCSAAAN** | Genes | 0 | 13 | 0 | 13 | 0 | 68 |
|  | Prob. | 0 | 0.01543943 | 0 | 0.01785714 | 0 | 0.0068 |
|  | p value | 1 | 0.02294981 | 1 | 0.01314729 | 1 |  |
| **TCF/LEF1 V$LEF1TCF1_Q4 MCTTTGWWSNY** | Genes | 125 | 293 | 46 | 241 | 79 | 3410 |
|  | Prob. | 0.38343558 | 0.347981 | 0.47916667 | 0.33104396 | 0.34649123 | 0.341 |
|  | p value | 0.06033747 | 0.34142559 | 0.00348558 | 0.70913217 | 0.43159755 |  |
| **V$MYCMAX_B GCCAYGYGSN** | Genes | 60 | 176 | 19 | 155 | 42 | 1758 |
|  | Prob. | 0.18404908 | 0.20902613 | 0.19791667 | 0.21291209 | 0.18421053 | 0.1758 |
|  | p value | 0.35255396 | 0.01106284 | 0.29407657 | 0.00883307 | 0.37294715 |  |

* p values from one-tailed two proportion Z-statistics

** red: p<0.05, orange: 0.05<p<0.1
