# Supplementary material for: TERT Promotes Epithelial Proliferation through Transcriptional Control of a Myc- and Wnt-Related Developmental Program
Source: PLoS Genet. 2008 Jan 18;4(1):e10. doi: 10.1371/journal.pgen.0040010 (PMC2211538; doi:10.1371/journal.pgen.0040010)
Supplement: Supplementary file 16 [file pgen.0040010.st010.doc]

**Table S10. TERT and TERT^ci^ do not induce hair follicle neomorphogenesis.**

| Genotype | Number of hair follicles per microscopic field^*^ | Number of mice analyzed |
| --- | --- | --- |
| Control^†^ | 5.00 ± 0.71 | 17 |
| iK5-TERT | 5.17 ± 0.75 | 6 |
| iK5-TERT^ci^ | 4.92 ± 0.51 | 12 |

* Hair follicle density was analyzed from day 50~60 dorsal skin sections at 20X magnification.

† Control consists of Non-Tg, K5-tTA, tetop-TERT and tetop-TERT^ci^ mice.

‡ One-way ANOVA p=0.749. Not significant.
